# Deep-learning-enabled antibiotic discovery through molecular de-extinction

**DOI:** 10.1038/s41551-024-01201-x

**Published:** 2024-06-11

**Authors:** Fangping Wan, Marcelo D. T. Torres, Jacqueline Peng, Cesar de la Fuente-Nunez

**Affiliations:** 1 Machine Biology Group, Departments of Psychiatry and Microbiology, Institute for Biomedical Informatics, Institute for Translational Medicine and Therapeutics, Perelman School of Medicine, University of Pennsylvania, Philadelphia, PA USA; 2 Departments of Bioengineering and Chemical and Biomolecular Engineering, School of Engineering and Applied Science, University of Pennsylvania, Philadelphia, PA USA; 3 Department of Chemistry, School of Arts and Sciences, University of Pennsylvania, Philadelphia, PA USA; 4 Penn Institute for Computational Science, University of Pennsylvania, Philadelphia, PA USA; 5 Graduate Group in Genomics and Computational Biology, University of Pennsylvania, Philadelphia, PA USA

**Keywords:** Antimicrobials, Drug discovery

## Abstract

Molecular de-extinction aims at resurrecting molecules to solve antibiotic resistance and other present-day biological and biomedical problems. Here we show that deep learning can be used to mine the proteomes of all available extinct organisms for the discovery of antibiotic peptides. We trained ensembles of deep-learning models consisting of a peptide-sequence encoder coupled with neural networks for the prediction of antimicrobial activity and used it to mine 10,311,899 peptides. The models predicted 37,176 sequences with broad-spectrum antimicrobial activity, 11,035 of which were not found in extant organisms. We synthesized 69 peptides and experimentally confirmed their activity against bacterial pathogens. Most peptides killed bacteria by depolarizing their cytoplasmic membrane, contrary to known antimicrobial peptides, which tend to target the outer membrane. Notably, lead compounds (including mammuthusin-2 from the woolly mammoth, elephasin-2 from the straight-tusked elephant, hydrodamin-1 from the ancient sea cow, mylodonin-2 from the giant sloth and megalocerin-1 from the extinct giant elk) showed anti-infective activity in mice with skin abscess or thigh infections. Molecular de-extinction aided by deep learning may accelerate the discovery of therapeutic molecules.

## Abstract (web summary)

Deep learning can be used to mine the proteomes of all available extinct organisms for the discovery of compounds with antibiotic properties.

## Main

With antimicrobial-resistant infections causing approximately 1.27 million deaths annually worldwide and projections indicating a potential 10 million annual fatalities by 2050 (ref. ^1^) in the absence of effective new drugs, urgent measures are required to combat antibiotic resistance. Furthermore, according to the World Health Organization, by 2030, around 24 million individuals could face extreme poverty due to the high cost of treating these infections^1^.

Molecules serve as records of evolutionary history^2^ and may provide blueprints for therapeutic design. We recently introduced the term molecular de-extinction^3^, referring to the resurrection of extinct molecules of life to tackle contemporary challenges such as antibiotic resistance. By uncovering a new sequence space of previously unexplored molecules, molecular de-extinction offers a promising approach to expand our vision of life’s molecular diversity while helping unveil molecules that may play a role in host immunity throughout evolution. Molecular de-extinction has already yielded preclinical antibiotic candidates such as neanderthalin-1 (A0A343EQH4-LAM11)^3^.

Approaches of computational and artificial intelligence have recently been developed for antibiotic discovery^4–7^. For example, machine-learning (ML) models have been used to generate antibiotics^8,9^ and to predict antimicrobial activity^10–12^, haemolysis^13^ and antimicrobial resistance^14,15^. Recently, computational methods have been developed to discover new antibiotics through proteome mining^3,16^. We previously mined the human proteome as a source of antibiotics and identified encrypted peptides (EPs), fragments within proteins that possess antimicrobial properties^16^. We hypothesized that EPs exist not only in modern humans but also throughout evolution. Thus, subsequently, through paleoproteome mining and ML, we identified similar molecules in ancient humans^3^. Altogether, these recent computational efforts have greatly accelerated our ability to discover new preclinical antibiotic candidates^3,5,16^.

**Fig. 1:**
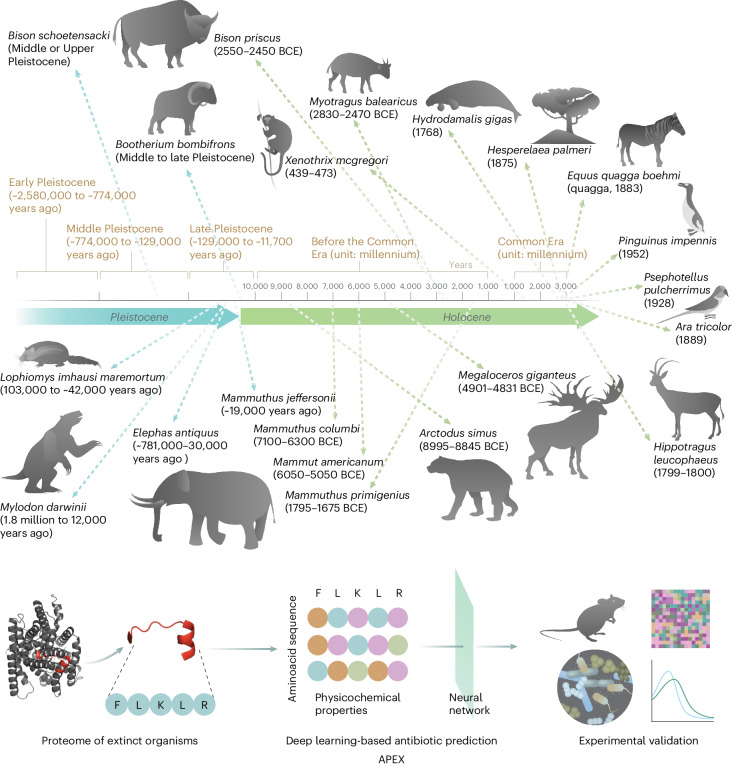
Molecular de-extinction of antibiotics from ancient proteomes using deep learning. All available proteomes of extinct organisms were mined by APEX, our deep learning algorithm. Amino acid sequences ranging from 8 to 50 amino acid residues within proteins from extinct organisms were inputted into multitask deep learning models that trained on both public and in-house peptide data to evaluate the potential antimicrobial activity. The highest ranked peptides based on predicted antimicrobial activities were then selected and thoroughly characterized against clinically relevant pathogens both in vitro and in animal models. The mechanism of action, physicochemical features and synergistic interactions of these peptides were also assayed. The dates report the approximate extinction date or period for the organisms studied. The protein and peptide structures shown in the figure were created with PyMOL Molecular Graphics System, version 2.1 Schrödinger, LLC.

In this Article, we introduce antibiotic peptide de-extinction (APEX; Fig. 1), a new multitask deep learning approach used to systematically mine all available proteomes of extinct organisms (that is, the ‘extinctome’) for antibiotic discovery.

## Results and discussion

### Prediction of antimicrobial activity from peptide sequence using deep learning

Recent computational advances have enabled the exploration of proteomes for antibiotic discovery^3,16^. To accelerate these efforts, we have developed APEX, a deep learning model that uses a multitask learning architecture to predict the antimicrobial activity of peptides (Supplementary Fig. 1). APEX was trained on peptide data from both our in-house dataset and the publicly available Database of Antimicrobial Activity and Structure of Peptides (DBAASP)^17^. APEX utilizes an encoder neural network, combining recurrent and attention neural networks (Supplementary Fig. 1), to extract hidden features from peptide sequences. The encoder neural network was then coupled with multiple downstream neural networks to predict antimicrobial activity according to the peptide source (that is, from in-house or public datasets). Specifically, in our approach, the extracted hidden features were fed into two separate, fully connected neural networks (FCNNs): one neural network was trained on our in-house peptide dataset and used to predict antimicrobial activity against specific bacterial strains (that is, a regression problem); the other neural network was trained on publicly available antimicrobial peptides (AMPs) and inactive peptides (referred to here as non-AMPs) derived from the DBAASP dataset^17^ to perform a binary classification (that is, a classification problem). We defined as non-AMPs those peptides that were not active at the range of concentrations selected as threshold, that is, minimum inhibitory concentration (MIC) > 30 μmol l^−1^ (‘Publicly available AMP sequences’ in Methods). Any publicly available sequences that overlapped with our in-house dataset were removed from the model training to prevent label information leakage. As the encoder neural network was trained on both the in-house and the public datasets, the incorporation of the latter FCNN served as a data augmentation strategy to improve prediction performance.

To train APEX, we utilized a combination of 988 in-house peptides and 5,093 and 5,500 publicly available AMPs and non-AMPs, respectively, obtained from DBAASP^17^. Our in-house dataset included 14,738 antimicrobial activity data values obtained from 34 bacterial strains. To assess APEX’s antimicrobial prediction performance, we randomly split our in-house dataset into a cross-validation (CV) set and an independent set, consisting of 790 and 198 peptides, respectively. Fivefold CV was first used to tune the hyperparameters on the CV set, while the independent set was used to evaluate the final prediction performance of ML models trained on the CV set with determined hyperparameters.

To compare the performance of our deep learning approach with simple ML predictors, we implemented several baseline ML models, including elastic net, linear support vector regression, extra-trees regressor, random forest and gradient boosting decision tree, and trained and evaluated them on the same datasets. The hyperparameter ranges searched for each ML model are provided in Supplementary Tables 1–4. On the CV set, our APEX model with the best hyperparameter combination outperformed all baseline ML models in terms of predicted activity for most bacteria, focusing particularly on 11 bacterial pathogens known as the ESKAPEE (*Enterococcus faecium*, *Staphylococcus aureus*, *Klebsiella pneumoniae*, *Acinetobacter* *baumannii*, *Pseudomonas aeruginosa*, *Enterobacter* spp. and *Escherichia coli*) pathogens. These pathogens are classified by the World Health Organization as the most dangerous threats to our society^18^.

Specifically, APEX outperformed all baseline ML models on most pathogen-specific MIC predictions in terms of *R*^2^, Pearson and Spearman correlations (that is, single APEX in Supplementary Figs. 2–7 and Supplementary Tables 5–7). The average *R*^2^, Pearson and Spearman correlations of the baseline ML methods were, at best, 0.378, 0.584 and 0.523, respectively. Compared to the baseline, the single best APEX obtained similar *R*^2^ = 0.369 and better Pearson correlation = 0.621 and Spearman correlation = 0.556 (Supplementary Tables 5–7).

To improve the prediction performance, we adopted an ensemble learning approach by selecting the top eight APEX models (with different neural network architectures and training strategies; for details, see ‘Hyperparameter tuning, model evaluation and ensemble learning’ in Methods; Supplementary Figs. 8–10) ranked by the average *R*^2^ on the CV set and obtained the final predictions by averaging the predictions from these models. The ensemble learning approach (that is, ensemble APEX v1) increased the prediction performance to 0.473, 0.669 and 0.594 in terms of *R*^2^, Pearson correlation and Spearman correlation, respectively (Supplementary Figs. 2–7 and Supplementary Tables 5–7).

APEX involved the following multitask training steps: (1) we used a single FCNN to simultaneously predict peptide antimicrobial activity for the 34 strains tested; (2) we augmented the training data by incorporating another FCNN to predict whether peptides from public databases (either AMPs or non-AMPs) were antimicrobial; and (3) we imposed a multitask training constraint on the learnable weights of the last layer in the species-specific antimicrobial prediction FCNN. Briefly, this last constraint encouraged the model to give similar prediction results for similar bacteria (defined by having a shorter phylogenetic distance from each other).

To evaluate the effectiveness of adding publicly available AMPs/non-AMPs data into our training as well as that of using the multitask training constraint, we conducted an ablation study by dropping these two parts during training and evaluated the corresponding prediction performance on the CV set. We observed that dropping the publicly available AMPs/non-AMPs data from the training set substantially decreased prediction performance (Supplementary Figs. 11–13 and Supplementary Tables 8–10). Adding the multitask training constraint led to either increased or decreased prediction performance depending on the target bacterial strain (Supplementary Figs. 11–13 and Supplementary Tables 8–10). Thus, we treated the presence or absence of the multitask training constraint as an additional hyperparameter, and we allowed subsequent hyperparameter tuning to decide whether to use the constraint or not. It is noteworthy that, among the top eight selected APEX models, six used the constraint during model training (Supplementary Table 11).

**Fig. 2:**
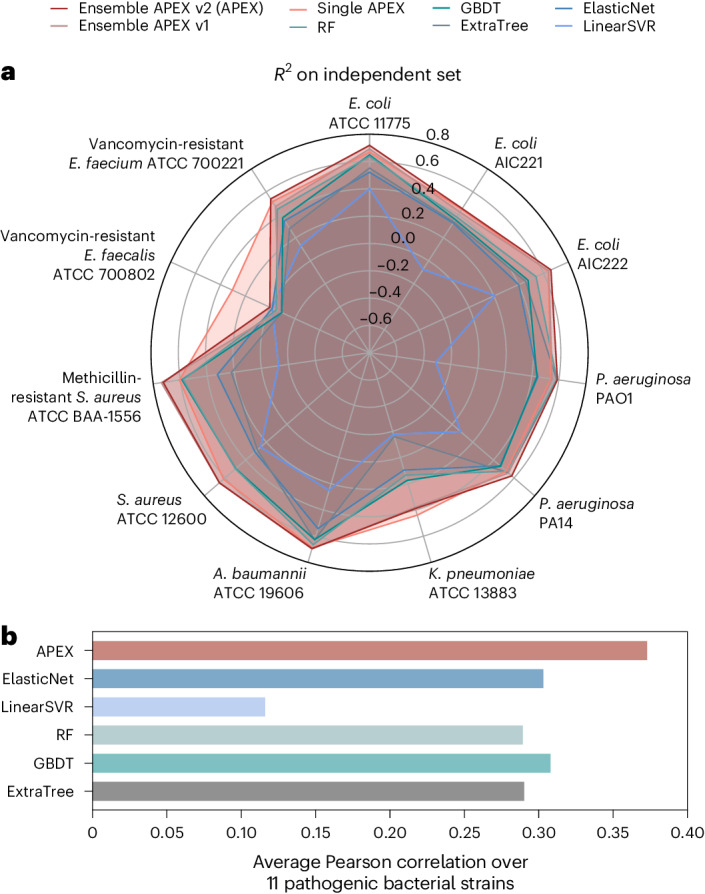
APEX prediction performance and comparison with other models. **a**, Radar chart showing *R*^2^ correlation in terms of species-specific antimicrobial activity prediction on an independent dataset (a held-out subset from our in-house peptide dataset) for various ML models. The radius reflects the *R*^2^ value for each of the models. APEX variants outperformed the baseline ML methods for most of the pathogens analysed. RF, random forest; GBDT, gradient boosting decision tree; ExtraTree, extra-tree regressor; ElasticNet, elastic net; LinearSVR, linear support vector regression. **b**, Mean of species-wise Pearson correlation of log_2_-transformed MICs between values obtained experimentally and predicted by various ML models. Evaluated dataset: 69 peptides were synthesized and tested.

Because model selection was based on the CV set, evaluating the prediction performance on the CV set alone may overestimate the generalization ability of APEX and the baseline models. Therefore, we trained the ML models with the determined hyperparameters on the whole CV set and evaluated their prediction performance on the independent set. Similar to the results obtained on the CV set, ensemble APEX v1 achieved an *R*^2^ value of 0.520, a Pearson correlation of 0.706 and a Spearman correlation of 0.582 on average (Fig. 2a, Supplementary Figs. 14–18 and Supplementary Tables 12–14), outperforming all baseline ML methods. In practice, to make prediction results more robust, a single ML model may be trained with different random seeds. We averaged the prediction results from all model copies to counter the potential stochastic behaviour caused by the choice of random seeds. For each APEX model selected, we trained five copies with different random seeds and created a second ensemble learning version (ensemble APEX v2) with 40 APEX models (that is, 8 APEX models × 5 copies). This ensemble learning approach increased the prediction performance to 0.546, 0.728 and 0.607 in terms of *R*^2^, Pearson correlation and Spearman correlation, respectively (Fig. 2a, Supplementary Figs. 14–21 and Supplementary Tables 12–14).

We then tested APEX’s predictive power compared to that of a scoring function used previously to discover antibiotics in the modern human proteome^16^. Given a peptide sequence, the scoring function^19^ uses hydrophobicity and net charge to compute a predictive score of antimicrobial potential. As the 56 human peptide antibiotics validated experimentally for antimicrobial activity in our previous work^16^ are part of our in-house dataset, we used them here as the test set and used the rest of our in-house dataset (932 peptides) for model training and selection. For the dataset consisting of the 56 validated human peptide antibiotics, the ensemble APEX v2 achieved highest values for Pearson and Spearman correlations in most cases (Supplementary Figs. 22–25 and Supplementary Tables 15–16). Lastly, during the subsequent prediction of the antimicrobial activities for the 69 synthesized EPs using ML models trained on the entire in-house dataset, APEX outperformed all baseline ML methods. Notably, APEX achieved the highest Pearson correlation for MIC prediction (Fig. 2b; more details in ‘In vitro antimicrobial activity of antibiotic molecules from extinct organisms’)*.* Collectively, these results substantiate our computational validation of APEX as the most accurate model for antimicrobial activity prediction in comparison to all the other models tested in this work (Fig. 2a,b and Supplementary Figs. 2–25). Based on these results, we decided to use the ensemble APEX v2 model (hereafter referred to as APEX) to mine extinct proteomes for antibiotics.

### Mining the extinctome for antibiotics, and sequence-space exploration

**Fig. 3:**
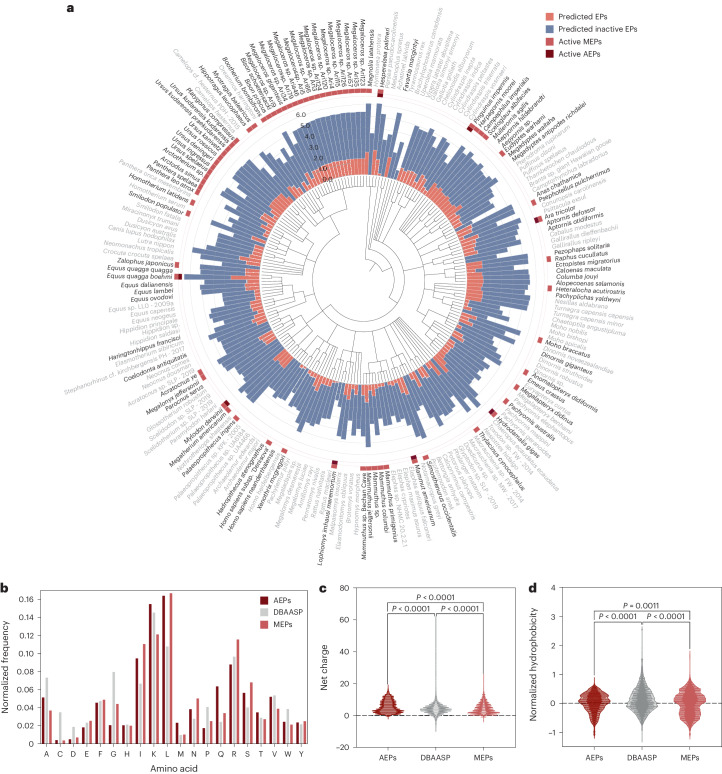
Antimicrobials identified by APEX in extinct organisms and their composition and physicochemical properties. **a**, Phylogenetic tree showing the extinct organisms scanned by APEX. Circular bars denote the log_10_-transformed average active (red) and inactive (blue) EPs discovered by APEX. A peptide was considered active when its predicted median MIC against the bacterial strains tested was ≤80 μmol l^−1^. The values were normalized by the number of proteins per organism scanned. The organisms whose EPs were selected for validation are highlighted in bold type. Extinct organisms that presented active EPs validated experimentally are indicated by a light red square and, within that group, those organisms encoding extinct sequences absent in extant organisms are highlighted with a dark red square. **b**, Amino acid frequency in AEPs and MEPs compared with known AMPs from the DBAASP database. AEPs present a higher frequency of the basic residue K, the aliphatic residue V, and uncharged polar residues (M, Q and T) than MEPs. **c**,**d**, Distribution of two physicochemical properties for peptides with predicted antimicrobial activity (AEPs and MEPs) and AMPs from DBAASP: net charge (**c**) and normalized hydrophobicity (**d**). Net charge directly influences the initial electrostatic interactions between the peptide and negatively charged bacterial membranes, and hydrophobicity directly influences the interactions of the peptide with lipids in the membrane bilayers. EPs from extinct organisms are slightly less hydrophobic and similarly have a net positive charge, compared with EPs from the modern human proteome^16^ or peptides from DBAASP. Statistical significance in **c** and **d** was determined using two-tailed *t*-tests followed by Mann–Whitney test; *P* values are shown in the graph. The solid line inside each box represents the mean value obtained for each group.

To mine the extinctome using APEX, we first collected 12,860 protein sequences from 208 extinct species obtained from the National Center for Biotechnology Information (NCBI) (Fig. 3a). After removing redundant sequences, we were left with 5,190 proteins. EPs were defined as substrings ranging from 8 to 50 amino acid residues within the protein sequences, which align with the lengths of most active antimicrobial peptides reported previously^16^. This resulted in 10,311,899 EP sequences. We then applied APEX to predict the antimicrobial activity of these compounds. This effort led to the identification of 37,176 peptides (Supplementary Dataset 1) predicted to show broad-spectrum antimicrobial activity with median MIC ≤ 80 μmol l^−1^. To test whether the compounds identified by APEX belonged to a new sequence space, we compared the identified 37,176 sequences to previously described peptides in the literature and contained within the DBAASP^17^ database. Briefly, for the EPs and DBAASP peptides, sequence alignment^20^ was used to calculate the pairwise peptide sequence similarity (the sequence similarity calculation procedure can be found under ‘Sequence similarity score’ in the Methods). For each sequence, we used its sequence similarities to all peptides (that is, DBAASP peptides and 37,176 EPs) as its feature representation. Next, we used the uniform manifold approximation and projection (UMAP)^21^ technique to reduce the dimension of the feature representations to a bidimensional (2D) space (Supplementary Fig. 26). While DBAASP peptides mostly fell within the central area of the UMAP-derived 2D space, molecules identified by APEX had a much wider spread (Supplementary Fig. 26), forming multiple distinct clusters that were not covered by DBAASP. These results revealed that sequences identified by APEX within the extinctome can belong to novel and previously unexplored parcels of sequence space.

### Differences between modern and archaic antibiotic molecules

To assess differences between modern EPs (MEPs, that is, sequences present in both extinct and extant organisms) and archaic EPs (AEPs, that is, sequences not found in the available proteomic data from extant organisms) and determine whether these sequences constitute new classes of antimicrobial peptides, we compared sequences identified by APEX (that is, 11,035 AEPs out of the 37,176 EPs identified above) with MEPs from the modern human proteome identified by an antimicrobial scoring function^16^. More details on our classification of MEPs and AEPs are provided in ‘Classification guidelines to identify AEPs’ in Methods.

First, for a direct comparison with the scoring function, we used APEX to find sequences within proteins from the modern human proteome, extracted these sequences and determined their amino acid compositions (Supplementary Fig. 27). In contrast to the scoring function, which considers and selects for net positive charge to identify sequences^16^, APEX selected sequences with a higher frequency of negatively charged residues (that is, aspartic acid and glutamic acid), as well as glycine and polar uncharged residues (that is, asparagine, glutamine and serine). These amino acid residues with net charge and hydrophobicity features are not relevant to the scoring function, which mostly considers net positive charge and amphiphilic peptide sequences. The EPs identified in modern human proteins^16^ by the scoring function also showed a higher content of cysteine, methionine, phenylalanine and arginine. Interestingly, lysine, which is preferentially scored by the scoring function, was slightly overrepresented in APEX-identified sequences (Supplementary Fig. 27).

Furthermore, we compared the amino acid composition of APEX’s sequences to conventional AMPs from DBAASP (Fig. 3b). Generally, molecules identified by APEX presented lower cysteine, aspartic acid and glycine content compared to AMPs from DBAASP (Supplementary Fig. 28). Peptides derived from proteins of extinct organisms also had a higher methionine and glutamine content compared to AMPs from DBAASP (Supplementary Fig. 29). The MEPs identified by APEX had a lower alanine, proline and tryptophan content but a higher isoleucine, leucine, asparagine and serine content than peptides within DBAASP (Supplementary Fig. 30). Comparative analysis between AEP and MEP sequences identified by APEX revealed an overrepresentation of methionine and glutamine in AEPs and of glycine in MEPs (Supplementary Fig. 31).

We then compared the physicochemical features contributing to antimicrobial properties^17^ (Supplementary Fig. 32). These analyses revealed that AEPs showed a lower amphiphilicity (amphiphilicity index <2; Supplementary Fig. 32a) but a slightly higher propensity to be disordered (disordered conformation propensity score from −0.5 to 1) than MEPs or AMPs (Supplementary Fig. 32b). These results indicate that interactions between AEPs and the bacterial membrane are likely to differ from those of standard AMPs, which are more amphiphilic and tend to assume a defined structure upon contact with the lipid from the membrane bilayer^17,22^ (Supplementary Figs. 32a,b and 33). In addition, to determine the potential toxicity and amphiphilicity of the peptides^22^, we assessed their theoretical tendency toward aggregation (Supplementary Fig. 32c) and the angle of the hydrophobic residues upon adopting a secondary structure (Supplementary Fig. 32d). These physicochemical parameters are predictive of how the peptides interact with membrane lipids to exert antimicrobial activity^14^. Interestingly, when comparing AEPs with either MEPs from the human proteome or AMPs from DBAASP, we found that AEPs were less prone to aggregate (in vitro aggregation propensity score <500) and presented a smaller predicted hydrophobic face (<100°) (Supplementary Fig. 32c,d). These results are a direct consequence of the higher frequency of uncharged polar residues in AEPs. To further investigate peptide structure, we obtained the predicted normalized hydrophobic moment (Supplementary Fig. 32e) and isoelectric point of AEPs (Supplementary Fig. 32f), which presented a low range of normalized hydrophobic moment (0 to 0.6) and clustered within a short isoelectric point range (9.5 to 13). These values, found for sequences in extinct organisms (AEPs), overlapped with those obtained for sequences in extinct and extant organisms (MEPs) as well as in AMPs from DBAASP (Supplementary Fig. 32e,f). The values aligned with the lower abundance of acidic residues compared to basic ones, particularly lysine, within AEPs (Fig. 3c,d).

Collectively, AEPs identified by APEX represent a distinct family of peptides with a higher abundance of uncharged polar residues and increased aliphatic content (particularly isoleucine and leucine) with respect to other classes of peptide antibiotics, including other EPs^16,23,24^ and AMPs^25^. There are a few AMP families, such as he-1, brevenins, pleurains and frog defensins^25^, having a lower net charge than most standard AMPs, which have more uncharged polar residues or a balance of positively charged and acidic residues, and whose antimicrobial activity depends on how their electronic density is distributed^17^. Like the AMPs in these families but unlike previously described EPs^16^ (Supplementary Fig. 27) or conventional AMPs (Supplementary Fig. 28), AEPs have a high abundance of uncharged polar residues^22^. Leucine and isoleucine, in particular, are structurally important: the stiffness of these bulky branched residues limits the internal flexibility of the peptide, whereas other aliphatic residues favour specific foldamers during the folding process^26^. The difference between the amino acid composition of known AMPs and that of AEPs and MEPs results in significantly different physicochemical features (Fig. 3c,d and Supplementary Fig. 32), reaffirming that the peptides identified by APEX are different from known antimicrobial peptides.

### In vitro antimicrobial activity of antibiotic molecules from extinct organisms

To further validate APEX’s predictive power in identifying active peptide sequences from extinct organisms, we synthesized and tested two non-overlapping sets of peptides: (1) 49 EPs predicted by a scoring function^16^ (Supplementary Fig. 34 and Supplementary Dataset 3) and (2) 69 EPs predicted by APEX (Fig. 3a and Supplementary Dataset 2) and found in 98 extinct species. While the 49 EPs predicted by the scoring function were found in both extinct and extant organisms (that is, all were classified as MEPs), the APEX-predicted sequences included many that were unique to extinct organisms (20 AEPs and 49 MEPs). APEX was built to predict species-specific antimicrobial activities, and 69 EPs were selected based on multiple selection criteria. Specifically, we ranked the 10,311,899 sequences derived from the extinct proteomes by median antimicrobial activities (that is, broad-spectrum activity) or selectivity against Gram-positive or Gram-negative pathogens. For each ranked list, we used the following criteria to filter out compounds: (1) length not ranging from 8 to 30 amino acid residues, (2) sequences that are present in our in-house dataset, (3) with high sequence similarity to known AMPs from public databases and (4) EPs that are present in the modern human proteome. For each resulting list, we grouped the peptides by their source organism and selected the top-ranked EPs that were not too hydrophobic to be chemically synthesized by solid-phase peptide synthesis. To ensure we explored a wider sequence space, we selected EP sequences from each list that were not sequentially too similar to each other (Supplementary Fig. 35). Detailed selection and filtering criteria can be found in Methods subsection ‘Antibiotic peptide screening and selection’.

Among the 69 EPs identified by APEX, 21 (5 AEPs and 16 MEPs) were derived from secreted proteins, while 48 (15 AEPs and 33 MEPs) were from non-secreted proteins. We included EPs from non-secreted proteins due to the limited annotations of extinct proteins. Filtering out unannotated secreted proteins would have restricted the sequence space explored, so we considered EPs from both secreted and non-secreted proteins. Out of the 21 peptides from secreted proteins identified by APEX, 4 were predicted to target Gram-positive bacteria selectively, 10 to target Gram-negative bacteria selectively and 7 to show broad-spectrum activity. Among the 48 peptides selected by APEX from non-secreted proteins, 19 were predicted to selectively target Gram-positive bacteria, 10 to selectively target Gram-negative bacteria and 19 to show broad-spectrum activity.

**Fig. 4:**
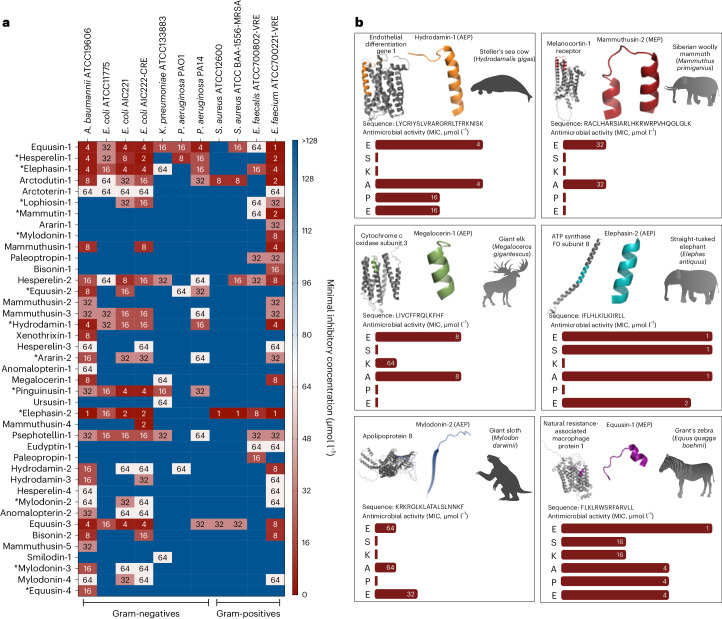
Antimicrobial activity profiles of sequences from the proteomes of extinct organisms. **a**, Heat map of the antimicrobial activities (μmol l^−1^) of the active antimicrobial agents from extinct organisms against 11 clinically relevant pathogens, including strains resistant to conventional antibiotics. Briefly, 10^6^ bacterial cells and serially diluted EPs (0–128 μmol l^−1^) were incubated at 37 °C. One day post treatment, the optical density at 600 nm was measured in a microplate reader to evaluate bacterial growth in the presence of the EPs from extinct organisms. MIC values in the heat map are the arithmetic mean of the replicates in each condition. **b**, Examples of active AEPs and MEPs from various extinct organisms, their parent protein and their activity profile against ESKAPE pathogens (*Enterococcus* spp., *S. aureus*, *K. pneumoniae*, *A. baumannii*, *P. aeruginosa*, *E. coli*). Antimicrobial activity is expressed as the MIC (μmol l^−1^), and activity bars are presented as −log_2_ MIC^14^. The data for the assays in **a** are the mean, and the experiments were performed in three independent replicates. AEPs in **a** are indicated by an asterisk (*). The protein and peptide structures shown in the figure were created with PyMOL Molecular Graphics System, version 2.1 Schrödinger, LLC.

**Fig. 5:**
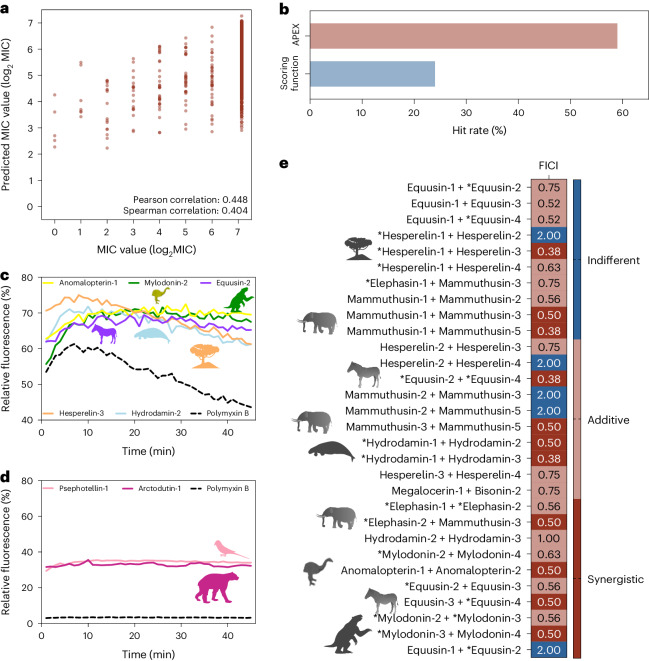
Antimicrobial activity, mechanism of action and synergy of antimicrobials from the proteomes of extinct organisms. **a**, Pan-bacterial Pearson and Spearman correlations of log_2_-transformed MICs between experimentally validated values and values predicted by APEX. **b**, Comparison between the hit rates of APEX and the scoring function previously described by Torres et al.^16^ to detect antibiotics in the modern human proteome. **c**, Cytoplasmic membrane depolarization by five antimicrobials from extinct organisms. The *A. baumannii* membrane was more strongly depolarized by the peptides than by the antibiotic polymyxin B. **d**, NPN permeabilization assays showing the effect of two antimicrobials from extinct organisms on the outer membrane of *A. baumannii*. Higher permeability was observed with the peptides than with the antibiotic polymyxin B. **e**, Heat map showing interactions between antimicrobials identified by APEX, expressed as the FICI. Most of the tested EP pairs from extinct organisms either synergized or had an additive effect against *A. baumannii* and *P. aeruginosa*; the latter was only tested against the peptide pair composed of equusin-1 and equusin-2 shown in the last row of the heat map. The data for the assays in **c**–**e** are the mean, and the experiments were performed in three independent replicates. AEPs in **e** are indicated by an asterisk (*).

Next, we synthesized the 20 AEPs and 49 MEPs identified by APEX from extinct organisms and experimentally determined their MICs for 11 clinically relevant bacterial pathogens (7 Gram-negatives and 4 Gram-positives), 10 of which are on the ESKAPEE pathogen list^18^ (Fig. 4a,b and Supplementary Dataset 2). The name of each source organism was used as the basis for our molecular de-extinction nomenclature. All experimentally determined MICs (log_2_ transformed) were compared to predictions generated by APEX, yielding Pearson and Spearman correlation values of 0.448 and 0.404, respectively (Fig. 5a), underscoring APEX’s substantial predictive power. In terms of species-specific antimicrobial activity prediction, APEX showed a high correlation of predicted versus experimentally validated activity (Pearson correlation >0.3) for *A. baumannii* ATCC (American Type Culture Collection) 19606; *E. coli* strains AIC221, AIC222 (a colistin-resistant strain) and ATCC 11775; *P. aeruginosa* strains PAO1 and PA14; and *E. faecium* ATCC 700221 (a vancomycin-resistant strain). All correlation results obtained for the 11 experimentally validated strains are shown in Supplementary Figs. 36–46. Furthermore, when the average species-specific Pearson correlations of the log_2_-transformed MIC predictions for the 11 pathogens of various baseline ML models were compared with that provided by APEX, our deep learning model yielded the most accurate predictions (Fig. 2b). Of the 69 synthesized peptides, 41 showed notable antimicrobial activity (that is, MIC ≤ 128 μmol l^−1^) against at least one bacterial strain, showing a 59% hit rate for identifying peptides with antimicrobial activity (Fig. 5b). This hit rate is higher than that of the scoring function^16^ (24%) when it was used for extracting antibiotics from the same extinct sources (Fig. 5b). In addition, 13 of the 41 active molecules identified by APEX and experimentally validated were AEPs, meaning that they were present in extinct but not in extant organisms.

We then used the selectivity score (see ‘Selectivity score’ from the Methods section for details) to quantify peptide selectivity. Specifically, among the 69 peptides synthesized, 20 were computationally predicted to selectively target Gram-negative pathogens and 23 to selectively target Gram-positive pathogens. For peptides predicted to be selective for Gram-negative pathogens, the Pearson correlation of selectivity scores calculated by experimentally validated and predicted MICs was 0.295. In addition, the mean Gram-negative selectivity score derived from experimental MICs for peptides selective for Gram-negatives was 0.783 and was 1.33 for the rest of the peptides tested. A *P* value of 0.013 for the one-sided Mann–Whitney *U*-test on the selectivity scores from these two lists suggested a statistically significant difference. This result showed APEX’s ability to discover peptide sequences that selectively target Gram-negative pathogens. However, we observed a weak Pearson correlation (0.11) between selectivity scores calculated by predicted and validated MIC values of peptides that were selected to target Gram-positive bacteria. The mean selectivity score of these peptides for Gram-positive bacteria was 1.02, indicating that the peptides did not selectively kill Gram-positive pathogens.

### Antibiotics identified in the extinctome

Several molecules identified by APEX showed excellent antimicrobial properties. These included anomalopterin-1, a peptide originating from the extinct moa species *Anomalopteryx didiformis*. This peptide is a fragment of the dynein axonemal heavy chain 3, which forms part of the microtubule-associated motor protein complex. Mylodonin-2, derived from the extinct South American giant sloth *Mylodon darwinii*, corresponds to a fragment of apolipoprotein B, a lipoprotein that functions as a ligand for the low-density lipoprotein receptor. Interestingly, peptides derived from the modern human apolipoprotein B have also been described as depolarizers of the membranes of Gram-negative bacterial pathogens^23^. Mylodonin-1 and megalocerin-1 are both fragments from the cytochrome c oxidase subunit 3 from *M. darwinii* and *Megaloceros* sp., respectively. This protein is the last enzyme in the mitochondrial electron transport chain.

Equusins 1 (MEP) and 2 (AEP), originating from the extinct Grant’s zebra *Equus quagga boehmi*, are derived from the natural resistance-associated macrophage protein 1 and the abnormal spindle-like microcephaly-associated protein, respectively. The natural resistance-associated macrophage protein 1 is essential for macrophage regulation and acts as a specific antiporter that fluxes metal ions in either direction against a proton gradient. The abnormal spindle-like microcephaly-associated protein is responsible for calmodulin-binding activity and plays a role in regulating the meiotic cell cycle, gamete generation, centrosome location maintenance and nervous system development.

Mammuthusin-2, derived from *Mammuthus primigenius*, originated from the melanocyte-stimulating hormone receptor, which regulates all types of melanocyte-stimulating hormone (α, β and γ). Elephasin-2, one of the most active antimicrobial molecules identified here, is found as a fragment of adenosine triphosphate (ATP) synthase F0 subunit 8 from the extinct *Elephas antiquus*. This protein is one of the main subunits responsible for ATP synthesis.

Hesperelin-3 is produced by both an extinct magnolia species (*Magnolia latahensis*) and an extinct palm tree species (*Hesperelaea palmeri*). It is a component of the protein ribulose bisphosphate carboxylase large chain. Ribulose bisphosphate carboxylase large chain catalyses two reactions: the carboxylation of d-ribulose 1,5-bisphosphate, which is the primary step in carbon dioxide fixation, and the oxidative fragmentation of the pentose substrate in photorespiration. The extinct manatee *Hydrodamalis gigas* yielded a fragment of the endothelial differential gene 1 (hydrodamin-1), which regulates endothelial cell differentiation, and a fragment from the von Willebrand factor (hydrodamin-2), a protein involved in haemostasis. Peptides derived from the von Willebrand factor have also been previously found in modern humans^16^.

### Secondary structure of antibiotic molecules from extinct organisms

Given that peptides identified by APEX were, on average, different from sequences predicted by the scoring function and previously reported AMPs in terms of physicochemical descriptors and amino acid residue composition, we decided to determine their secondary structure. When AMPs come into contact with bacterial membranes, they typically adopt an α-helical conformation due to their amphipathicity, net charge, hydrophobicity and length, all of which directly influence their secondary structure.

To computationally evaluate the alpha-helix structures and predict the secondary structures of peptides within the training dataset and the 69 EPs selected and validated, we utilized Single-Sequence Secondary Structure PREDictor (S4PRED)^27^. We then used the following ratio to quantify the abundance of α-helix structures in a given peptide dataset:

$$\begin{array}{l}{\rm{\alpha }} {\hbox{-}}{\rm{helix}}\; {\rm{ratio}}\\=\begin{array}{l}\frac{{\rm{the}\,{number}\,{of}\,{amino}\,{acid}\,{residues}\,{predicted}\,{to}\,{be}\,{\rm{\alpha }}{\hbox{-}}{helical}\,{in}\,a\,{dataset}}}{{\rm{total}\,{number}\,{of}\,{amino}\,{acid}\,{residues}\,{in}\,a\,{dataset}}}.\end{array}\end{array}$$

For 988 in-house peptides and 5,093 publicly available AMPs used for APEX model training, the α-helix ratios were 25.99% and 40.88%, respectively. For the 37,176 EPs predicted to have broad-spectrum antimicrobial activity by APEX (Supplementary Dataset 1), the α-helix ratio was 40.0%. For the 69 EPs (Supplementary Dataset 2) identified by APEX and selected for experimental validation, the ratio was 20.06%. The in-house peptides and publicly available AMPs used for APEX model training had a high α-helix ratio, confirming the dominance of α-helical structures in our training dataset. The high α-helix ratio for the 37,176 EPs identified by APEX further confirmed that our model captured this structure pattern from the training data. However, the ratio of α-helical structures for the 69 EPs we selected and validated was lower than those present in the training data. We think this resulted from applying the second of five different filtering steps (‘Antibiotic peptide screening and selection’) to ensure the selected peptides were sequentially different (and consequently structurally diverse) from known AMPs and EPs^16^.

To determine the secondary structure of the active sequences obtained from extinct organisms, we exposed them to a helix-inducing medium^28^ (trifluoroethanol in water, 3:2, *v*:*v*). Interestingly, most molecules synthesized and tested that were identified through the scoring function were not α-helical but instead had a relatively high content of anti-parallel β-structure and were largely unstructured (Supplementary Fig. 33a,c). By contrast, AEPs and MEPs identified by APEX showed predominantly helical structures under the analysed conditions, despite their unusual abundance of uncharged polar residues and low amphiphilicity (Supplementary Fig. 33b,d). These results shed light into the considerably higher success rate achieved by APEX compared to the scoring function. The greater prevalence of α-helical peptides and amphipathic structures within the APEX sequences enhances their interactions with the membrane, resulting in more effective membrane damage^22,29^ (Fig. 5c,d).

### Mechanism of action

The bacterial membrane is a common target for AMPs, where they engage in non-specific interactions with the lipid bilayer^22^. The antimicrobial activity of AMPs is influenced by their amino acid composition, distribution and various physicochemical characteristics such as amphiphilicity and hydrophobicity. To investigate the underlying mechanisms by which the peptides identified by APEX kill bacteria, we tested whether differences in the composition of AEPs and MEPs would affect their mechanism of action. The differences in composition assessed (Supplementary Figs. 26 and 35) include the content of uncharged polar and aliphatic residues (Fig. 3b and Supplementary Figs. 28–31) and different ranges of physicochemical features (Fig. 3c,d and Supplementary Fig. 32).

First, we tested whether AEPs and MEPs depolarized the cytoplasmic membrane of *A. baumannii*. We used the potentiometric fluorophore 3,3′-dipropylthiadicarbocyanine iodide (DiSC_3_-5) whose fluorescence is suppressed by its accumulation and aggregation within the cytoplasmic membrane. Upon disturbances in the transmembrane potential of the cytoplasmic membrane, this fluorophore migrates to the outer environment and emits fluorescence. Polymyxin B was used as a positive control in these experiments as it is a depolarizer that also permeabilizes and damages bacterial membranes. Notably, AEPs and MEPs depolarized the cytoplasmic membrane more effectively than polymyxin B (Fig. 5c and Supplementary Fig. 47) and than peptides previously found in modern human proteins^16^. The most potent depolarization effects were found for the following five peptides: anomalopterin-1, mylodonin-4, equusin-2, hesperelin-3 and hydrodamin-2 (Fig. 5c). We hypothesize that this increased depolarization results from their different amino acid composition, particularly the higher content of long aliphatic residues, such as leucine and isoleucine, in AEPs and MEPs compared to known AMPs.

To determine whether the compounds permeabilized the bacterial outer membrane, we performed 1-(*N*-phenylamino)naphthalene (NPN) assays. NPN, a lipophilic dye, fluoresces faintly in aqueous solutions but fluoresces substantially more when it encounters lipidic environments such as bacterial membranes. NPN can penetrate the bacterial outer membrane only if it is disrupted or compromised. In contrast to cells treated with the positive control polymyxin B or cells left untreated (untreated control group), bacteria exposed to the most active EPs at their MIC were, in general, not effectively permeabilized (Fig. 5d and Supplementary Fig. 48). The peptides that permeabilized the outer membrane, causing a higher increase in the uptake of the fluorescent probe, were the MEP psephotellin-1, derived from the ATP synthase unit of the extinct parrot *Psephotellus pulcherrimus*, and arctodutin-1, a MEP that is part of the enzyme NADH-ubiquinone oxidoreductase chain 5 from the extinct bear *Arctodus simus*. Both enzymes would have played a crucial role in the metabolism of these extinct organisms. These results showed that the two MEPs, psephotellin-1 and arctodutin-1, were more effective permeabilizers than most known AMPs and previously reported EPs derived from human proteins^16^. Outer membrane permeabilization is the most common mechanism of action described for AMPs and a key mechanistic driver for EPs derived from modern human proteins, such as natriuretic peptide, SCUB1-SKE25 and SCUB3-MLP22^16^. However, the permeabilization effect shown by AEPs and MEPs from extinct organisms was not as potent as that shown by EPs from the modern human proteome.

### Synergistic interactions

To investigate whether molecules from the same extinct organism (and close relatives) could synergize and thus potentiate each other’s activity against pathogens, we performed checkerboard assays^16^ at peptide concentrations ranging from four times the MIC to concentrations up to 64 times lower in the same conditions as used for the antimicrobial assays. First, we selected peptides according to their MIC values (Fig. 4a and Supplementary Fig. 49) for two pathogenic strains, *A. baumannii* ATCC 19606 and *P. aeruginosa* PAO1. The former is an opportunistic nosocomial pathogen that is increasingly resistant to antibiotics, leading to considerable mortality worldwide^30^. The latter is an intrinsically resistant bacterium associated with infections of the urinary tract, gastrointestinal tissue, skin and soft tissues and a cause of bacterial pneumonia, as well as a common opportunistic pathogen in cystic fibrosis patients^31^. Most of the combinations tested resulted in synergistic or additive interactions, calculated by using the fractional inhibitory concentration index^32^ (FICI; Fig. 5e). The MICs of combined EPs (Supplementary Fig. 49) were mostly twofold to threefold lower than those of the individual peptides, but in some cases, for example, equusin-1 and equusin-3, the MICs decreased by 64 times (from 4 μmol l^−1^ to 62.5 nmol l^−1^), reaching sub-micromolar concentrations that are comparable to the MICs of some of the most potent antibiotics^33^.

Several pairs of peptides showed particularly strong synergistic interactions, with FICI values as low as 0.38 for *A. baumannii*. These pairs included hesperelin-1 (AEP) and hesperelin-3 (MEP) from *H. palmeri*, mammuthusin-1 (MEP) and mammuthusin-3 (MEP) from *M. primigenius*, equusin-2 (AEP) and equusin-4 (AEP) from *E. q. boehmi* and hydrodamin-1 (AEP) and hydrodamin-3 (MEP) from *H. gigas* (Fig. 5e).

### Cytotoxicity assays

All 41 active AEPs and MEPs identified in the antimicrobial assays (Fig. 4a) were tested for cytotoxic activity against human embryonic kidney (HEK293T) cells (Supplementary Table 17), an extensively characterized cell line. This assay is widely used to assess the toxicity of antimicrobials, yielding highly reproducible results^34–36^. Of the peptides tested, 39 showed no notable cytotoxicity at the concentration range tested (8–128 μmol l^−1^). Cytotoxicity was detected for the AEP lophisin-1 from the ancient crested rat (*Lophiomys imhausi maremortum*) and for the MEP xenothrixin-1 from the extinct Jamaican monkey (*Xenothrix mcgregori*). The peptide dose that led to 50% cytotoxicity (CC_50_), estimated by non-linear regression, was 68.02 and 70.77 μmol l^−1^ for lophisin-1 and xenothrixin-1, respectively. Despite their slight toxicity, the concentration of these two peptides needed to exert antimicrobial activity was, respectively, 8 and 8.84 times lower than their CC_50_ values, underscoring their potential as antimicrobials. The CC_50_ values for the other 39 peptides were higher than the maximum concentration analysed, reinforcing the overall excellent safety profiles of this class of peptides.

### Resistance to proteolytic degradation

The AEPs hydrodamin-1, elephasin-2 and mylodonin-2 and the MEPs mammuthusin-2 and megalocerin-1 were selected for further stability and animal studies because of their potent antimicrobial activity (Fig. 4a) and good safety (Supplementary Table 17) profiles. To assess their stability in the presence of human proteases, we exposed these peptides to human serum, and aliquots were collected and analysed for 6 h at 37 °C. The AEP elephasin-2 and the MEP mammuthusin-2, both from organisms belonging to the same taxonomic order (that is, *Proboscidea*), showed the highest resistance to proteolytic degradation with ~40% peptide remaining after 6 h of exposure (Supplementary Fig. 50). Mammuthusin-2 presented slower degradation kinetics, whereas elephasin-2 was present at ~40% within the first 30 min of the experiment. All other AEPs and MEP analysed quickly degraded in the first 30 min to 1 h of the experiment (Supplementary Fig. 50).

### Anti-infective efficacy of antibiotic molecules in animal models

To assess whether the active AEPs and MEPs had anti-infective efficacy in vivo, we tested them in preclinical mouse models of skin abscess^36^ and thigh infection^16,37^. Five molecules were tested with a single dose at their MIC concentration after the infection was established. We selected the following five compounds having a wide range of MIC values (1–64 μmol l^−1^) when tested in vitro against *A. baumannii*: the MEP elephasin-2 (MIC = 1 μmol l^−1^, 1.3 mg kg^−1^) from *E. antiquus*, the AEP hydrodamin-1 (MIC = 4 μmol l^−1^, 9.5 mg kg^−1^) from *H. gigas*, the MEP megalocerin-1 (MIC = 8 μmol l^−1^, 10.9 mg kg^−1^) from *Megaloceros* sp., the MEP mammuthusin-2 (MIC = 32 μmol l^−1^, 78.1 mg kg^−1^) from *M. primigenius* and the AEP mylodonin-2 (MIC = 64 μmol l^−^^1^, 104.5 mg kg^−1^) from *M. darwinii*.

**Fig. 6:**
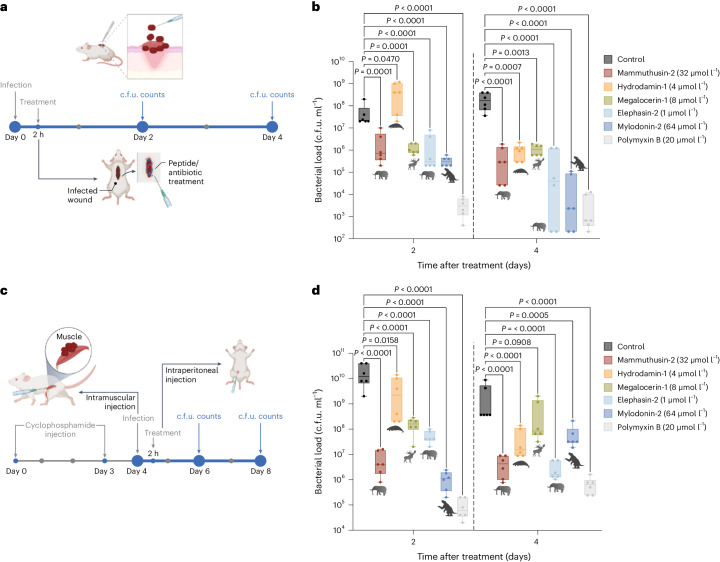
Anti-infective activity of antibiotic molecules in animal models. **a**, Schematic of the skin abscess mouse model used to assess the anti-infective activity of selected antimicrobials from extinct organisms (*n* = 6) against *A. baumannii* ATCC 19606. **b**, The molecules mammuthusin-2 (*M. primigenius*), hydrodamin-1 (*H. gigas*), megalocerin-1 (*Megalocerus* sp.), elephasin-2 (*E. antiquus*) and mylodonin-2 (*M. darwinii*), administered at their MIC in a single dose, inhibited the proliferation of the infection for up to 4 days after treatment compared to the untreated control group. Elephasin-2 and mylodonin-2 cleared the infection in some of the mice, with activity comparable to that of the antibiotic used as control, polymyxin B. **c**, Schematic of the neutropenic thigh infection mouse model in which EPs from extinct organisms were injected intraperitoneally. Anti-infective activity against *A. baumannii* ATCC 19606 was assessed 2 and 4 days after intraperitoneal peptide administration (*n* = 6). **d**, Two days after intraperitoneal injection, mylodonin-2 at its MIC reduced *A. baumannii* ATCC19606 infection as much as polymyxin B, compared to the untreated control group. Four days post treatment, mammuthusin-2 and elephasin-2 showed the same level of activity as polymyxin B. Statistical significance in **b** and **d** was determined using one-way ANOVA followed by Dunnett’s test; *P* values are shown in the graph. For the boxplots, the centre line represents the mean, the box limits the first and third quartiles and the whiskers (minima and maxima) 1.5 × the interquartile range. The solid line inside each box represents the mean value obtained for each group. Panels **a** and **c** were created with BioRender.com. Source data

In the skin abscess infection model, mice were infected with bacterial loads (10^6^ cells of the pathogen *A. baumannii*) (Fig. 6a). Each molecule was administered as a single dose over the infected area. After 2 days, bacterial counts showed that all molecules tested, except hydrodamin-1, markedly reduced the bacterial load by 2–3 orders of magnitude. These results highlight the potential anti-infective activity of these antimicrobials (Fig. 6b). After 4 days, the peptides reduced the infection by 3–5 orders of magnitude. The peptide hydrodamin-1, which was not active during the first 2 days post infection, showed activity by day 4 (Fig. 6b). The results obtained for the more active EPs tested (elephasin-2 and mylodonin-2) indicated antibacterial activity that was comparable to that of the widely used antibiotic polymyxin B, which was used as an antimicrobial control (Fig. 6b). Changes in weight, a surrogate measure of toxicity, were monitored from the time of the bacterial administration. No variations in weight, damage to the skin tissue or other harmful consequences induced by the molecules were observed in the mice throughout the experiments (Supplementary Fig. 51).

Next, we tested the anti-infective efficacy of the molecules using an established preclinical model particularly suited to assess the translatability of potential antibiotics. Using a murine deep thigh infection model, we assessed the efficacy of elephasin-2, hydrodamin-1, megalocerin-1, mammuthusin-2 and mylodonin-2, which were administered after the establishment of the intramuscular thigh infection (Fig. 6c). Briefly, mice were rendered neutropenic by cyclophosphamide treatment before intramuscular injection of 10^6^ *A. baumannii* cells (Fig. 6c). Next, a single dose of each peptide at its MIC was injected intraperitoneally. Two days and 4 days post treatment, all peptides tested, except for hydrodamin-1 at day 2 post treatment, had reduced the bacterial load by 2–4 orders of magnitude compared to the untreated control group (Fig. 6d). Two days post treatment, mylodonin-2 from *M. darwinii* presented the most potent activity, which was comparable to that of the positive control antibiotic, polymyxin B (4–5 orders of magnitude reduction in bacterial counts). Four days post treatment, elephasin-2 from *E. antiquus* and mammuthusin-2 from *M. primigenius* had decreased the bacterial loads by 3–4 orders of magnitude, resulting in similar levels as those in the mice treated with polymyxin B (Fig. 6d). None of the peptides tested were harmful to the mice based on weight monitoring during the experimental period (Supplementary Fig. 52).

These substantial in vivo results with two different preclinical mouse models showed that two AEPs (elephasin-2 and mylodonin-2) and one MEP (mammuthusin-2) displayed anti-infective efficacy comparable to that of a widely used antibiotic under physiologically relevant conditions, underscoring the potential of molecular de-extinction as an approach for antibiotic discovery.

This systematic analysis of all extinct organisms as a source of previously unrecognized antimicrobials shows the concept of molecular de-extinction. In addition, our deep learning model APEX outperforms previous work in this emerging area^3^ and constitutes an important method for antibiotic discovery. Molecular de-extinction enables the exploration of new sequence space, expanding our vision of molecular diversity and potentially unlocking new biology. We hypothesize that the molecules identified here may play a role in immunity throughout evolution, and future work will be needed to further test this notion. Finally, our approach yielded preclinical candidates with activity comparable to the standard of care, such as polymyxin B, highlighting its broad potential applications in biotechnology and medicine. In sum, using deep learning, we have mined the proteomes of all available extinct organisms and have identified antibiotics effective against some of the bacterial pathogens most dangerous to our society.

### Limitations of the study

There are limitations to leveraging deep learning to establish molecular de-extinction as a framework for antibiotic discovery. For example, our deep-learning model is purely sequence-based and does not contain structural information. Although this has advantages, structural and three-dimensional descriptors may be incorporated in the future to increase our model’s accuracy to predict antimicrobial activity. The model is also limited by the number of sequences present in our in-house dataset. Future work will focus on expanding this dataset to characterize larger spaces of peptide sequence and antimicrobial activity. Another limitation of our approach is the dearth of information available on extinct proteins, as most available proteomes so far have yielded only a few dozen proteins with reliable sequencing information. Our AEP classification was similarly limited by currently available extant proteomes, which may change in the future as new proteomes are revealed.

Overall, this work is a proof-of-concept demonstration of the de-extinction of antimicrobial molecules from extinct organisms by combining deep learning with wet-lab validation both in vitro and in animal models. Our approach reveals an untapped source of antibiotics. Furthermore, artificial intelligence and molecular de-extinction hold the potential to yield other medicinal discoveries.

## Methods

### Proteomes of extinct organisms

We collected extinct organisms from the NCBI taxonomy browser (https://www.ncbi.nlm.nih.gov/Taxonomy/taxonomyhome.html/index.cgi?chapter=extinct, access time: December 2021). For each species, we checked the corresponding Entrez records and downloaded the available protein sequences. In total, we retrieved 208 extinct species and a total of 12,860 protein sequences (5,190 non-redundant protein sequences) from them.

### Modern human proteome

To construct the human proteome, we downloaded 20,388 reviewed *Homo sapiens* proteins (20,307 unique ones) from UniProt (https://www.uniprot.org/).

### In-house peptide dataset

We utilized our high-quality in-house peptide dataset to train and evaluate APEX. In total, the dataset contained 14,738 antimicrobial activity measurements obtained by determining the MIC of 988 peptides and 34 bacterial strains, including the following, which were used to train our model: *E. coli* ATCC 11775, *P. aeruginosa* PAO1, *P. aeruginosa* PA14, *S. aureus* ATCC 12600, *E. coli* AIC221, *E. coli* AIC222, *K. pneumoniae* ATCC 13883, *A. baumannii* ATCC 19606, *Akkermansia muciniphila* ATCC BAA-835, *Bacteroides fragilis* ATCC 25285, *Bacteroides vulgatus* (*Phocaeicola vulgatus*) ATCC 8482, *Collinsella aerofaciens* ATCC 25986, *Clostridium scindens* ATCC 35704, *Bacteroides thetaiotaomicron* ATCC 29148, *B. thetaiotaomicron* ∆*tdk* ∆*lpxF* (background: VPI 5482)^38^, *Bacteroides uniformis* ATCC 8492, *Bacteroides eggerthi* ATCC 27754, *Clostridium spiroforme* ATCC 29900, *Parabacteroides distasonis* ATCC 8503, *Prevotella copri* DSMZ 18205, *Bacteroides ovatus* ATCC 8483, *Eubacterium rectale* ATCC 33656, *Clostridium symbiosum* ATCC 14940, *Ruminococcus obeum* ATCC 29174, *Ruminococcus torques* ATCC 27756, methicillin-resistant *S. aureus* ATCC BAA-1556, vancomycin-resistant *Enterococcus faecalis* ATCC 700802, vancomycin-resistant *E. faecium* ATCC 700221, *E. coli* Nissle 1917, *Salmonella enterica* ATCC 9150 (BEIRES NR-515), *S. enterica* (BEIRES NR-170), *S. enterica* ATCC 9150 (BEIRES NR-174) and *Listeria monocytogenes* ATCC 19111 (BEIRES NR-106).

Inactive data points, that is, MIC values higher than 128 μmol l^−1^, were labelled 140 μmol l^−1^. All antimicrobial activities were transformed by $-{\log }_{10}\frac{\rm{MIC}\; \rm{value}}{1,000,000}$ and were treated as labels to be predicted in the ML setting. To perform hyperparameter tuning and prediction performance evaluation, our in-house dataset was randomly split into a CV set and an independent set, which consisted of 790 and 198 peptides (that is, an 80%:20% split), respectively. Here the CV set was used to determine the optimal hyperparameters for ML models. ML models trained with determined hyperparameters on the CV set were evaluated on the independent set to measure their generalizability.

### Publicly available AMP sequences

We augmented the peptide training data by incorporating publicly available AMPs and non-AMPs into our deep learning model training. Public AMP data were retrieved from DBAASP^17^. Peptide sequences that consisted of only the 20 canonical or unknown amino acid residues were selected. The unknown amino acids were denoted by X, which corresponds to any possible canonical amino acid residues usually present within proteins having isoforms whose composition was undetermined because there were issues with metagenomic studies. Any peptide sequences that overlapped with our in-house peptide database were removed. As a peptide may have multiple MIC values for different bacterial species, we used the median MIC value to binarize the data. By using a stringent cut-off (that is, AMPs with MIC ≤ 30 μmol l^−1^), we created a balanced binary classification dataset (5,093 AMPs and 5,500 non-AMPs) for data augmentation and model training. To compare physicochemical properties, 14,114 peptides consisting of 20 canonical amino acids and having sequence length ≥4 amino acid residues were retrieved. We labelled this group of peptides as the DBAASP dataset.

### Physicochemical properties of peptides

To analyse the physicochemical properties of all peptide datasets (DBAASP, EPs generated by the scoring function^16^ and compounds identified by APEX), we used the DBAASP server to calculate the following 12 physicochemical properties that are usually considered in the design and study of peptide antibiotics^17^: normalized hydrophobic moment, normalized hydrophobicity, net charge, isoelectric point, penetration depth, tilt angle, disordered conformation propensity, linear moment, propensity to aggregation in vitro, angle subtended by the hydrophobic residues, amphiphilicity index and propensity to poly proline II (PPII) helix. We used the Eisenberg and Weiss scale (the consensus scale) as the hydrophobicity scale^39^.

### Secreted protein labelling

As proteins from extinct organisms are not as well annotated as those from extant organisms, we resorted to Orthologous Matrix (OMA)^40^ and DeepGOWeb^41^ to predict Gene Ontology (GO) terms from protein sequences. Given a protein sequence, if any of its GO terms predicted by OMA or DeepGOWeb corresponds to an extracellular region (GO:0005576) or a child of extracellular region in a GO-directed acyclic graph, then we considered this sequence to be secreted. Among proteins collected from extinct organisms, 157 sequences were labelled as secreted.

### Peptide sequence encoding

We treated a peptide as a sequence of amino acids. We further added two special characters as start (that is, ‘1’) and terminal symbols (that is, ‘2’) to the beginning and the end of this sequence, respectively. For each amino acid within the sequence, we used the AAindex^42^, which is a 566-dimensional vector storing various physicochemical and biochemical properties of each amino acid to represent it. Non-amino acid symbols and unknown amino acids were represented by 566-dimensional zero vectors. In this work, we only considered peptides shorter than 50 residues to ensure that they could be synthesized by solid-phase peptide synthesis. We created a fixed size input by zero-padding each sequence to maximum length, so that each peptide sequence could be represented by a matrix ${\boldsymbol{x}}\in {{\mathbb{R}}}^{52\times 566}$ (52 = maximum peptide length + two special characters).

### Bacterial distance

The bacterial taxonomy tree (bac120.tree) was downloaded from the Genome Taxonomy Database^43^. The phylogenetic distance matrix ${\boldsymbol{D}}{{\mathbb{\in }}{\mathbb{R}}}^{\rm{g\times g}}$, which stores distance between bacterial species, was calculated via the Python package DendroPy^44^. Here, *g* denotes the number of bacterial species. We converted the distance matrix ***D*** to a bacterial similarity matrix ${\boldsymbol{P}}{{\mathbb{\in }}{\mathbb{R}}}^{\rm{g\times g}}$ using the following function:

$${\boldsymbol{P}}=e^{\frac{-{\boldsymbol{D}}}{\rm{median}({\boldsymbol{D}})}},$$

where median(***D***) stands for the median value of matrix ***D***. We further binarized the similarity matrix ***P*** using the *K*-nearest neighbors algorithm, in which *K* was set to a heuristic number ${\rm{Ceiling}}(\frac{\sqrt{g}}{2})$. Here, Ceiling(**·**) stands for the ceiling function.

### APEX encoder architecture

APEX encoder started from a recurrent neural network (RNN) to process peptide sequence input ***x*** and extract its hidden features, ${{\boldsymbol{h}}}_{{\rm{rnn}}}\in {{\mathbb{R}}}^{52\times \rm{n}}$:

$${{\boldsymbol{h}}}_{{\rm{intermediate}}}={\rm{GRU}}\left({{\boldsymbol{x}}}\right),$$

$${{\boldsymbol{h}}}_{{\rm{rnn}}}={\rm{Layer}}{{\_}}{\rm{normalization}}\,({{\boldsymbol{h}}}_{{\rm{intermediate}}}),$$

where *n* and *GRU*(**·**) denote the hidden feature dimension and gated recurrent unit^45^, respectively. In addition, we added a layer normalization^46^ to stabilize the model training. On top of the RNN, we designed a two-layer attention neural network to better model feature (that is, amino acids) interactions globally and compress the hidden features to a lower-dimensional representation, respectively. Specifically, the first attention layer has the following form:

$${{\boldsymbol{a}}}_{{\bf{1}}}={\rm{softmax}}\left({\rm{concat}}\left({{\boldsymbol{h}}}_{{\rm{rnn}}},{\boldsymbol{x}}\right)\times {{\boldsymbol{W}}}_{{\rm{att}}{{1}}}\right),$$

$${{\boldsymbol{h}}}_{{\rm{att}}{{1}}}={{\boldsymbol{a}}}_{{{1}}}^{{\rm{T}}}\times {{\boldsymbol{h}}}_{{\rm{rnn}}},$$

where ${\rm{concat}}\left({{\boldsymbol{h}}}_{{\rm{rnn}}}{{,}}\;{\boldsymbol{x}}\right){\boldsymbol{\in }}{{\mathbb{R}}}^{52\times (n+566)}$ stands for concatenation operation along feature dimension and can be considered as a residual connection^47^, ${{\boldsymbol{W}}}_{{\rm{att}}{{1}}}{{\in }}{{\mathbb{R}}}^{(n+566)\times 52}$ is the learnable weights in this attention layer, ${\rm{softmax}}{{(}}\bullet {{)}}$ stands for the softmax function, ${{\boldsymbol{a}}}_{{{1}}}{\boldsymbol{\in }}{{\mathbb{R}}}^{52\times 52}$ denotes the attention weights and ${{\boldsymbol{h}}}_{{\rm{att}}{{1}}}{\boldsymbol{\in }}{{\mathbb{R}}}^{52\times n}$ is the output of this attention layer. For the second attention layer, it can be written as follows:

$${{\boldsymbol{a}}}_{{{2}}}={\rm{softmax}}\left({{\boldsymbol{h}}}_{{\rm{att}}{{1}}}\times {{\boldsymbol{w}}}_{{\rm{att}}{{2}}}\right),$$

$${{\boldsymbol{h}}}_{{\rm{att}}{{2}}}={{\boldsymbol{a}}}_{{{2}}}^{{\rm{T}}}\times {{\boldsymbol{h}}}_{{\rm{att}}{{1}}},$$

where ${{\boldsymbol{w}}}_{{\rm{att}}{{2}}}{\boldsymbol{\in }}{{\mathbb{R}}}^{n\times 1}$ is the learnable weights in this attention layer, ${{\boldsymbol{a}}}_{{{2}}}{{\in }}{{\mathbb{R}}}^{52\times 1}$ denotes the attention weights and ${{\boldsymbol{h}}}_{{\rm{att}}{{2}}}{\boldsymbol{\in }}{{\mathbb{R}}}^{1\times n}$ is the output of the second attention layer. In addition, we used a learnable linear transformation with weight matrix ${{\boldsymbol{W}}}_{{{fc}}}{{\in }}{{\mathbb{R}}}^{n\times m}$ and bias term ${{\boldsymbol{b}}}_{{{fc}}}{{\in }}{{\mathbb{R}}}^{1\times m}$ to create the final hidden representation ${\boldsymbol{h}}{{\in }}{{\mathbb{R}}}^{1\times m}$ for a peptide:

$${\boldsymbol{h}}={{\boldsymbol{h}}}_{{\rm{att}}{{2}}}{\times {\boldsymbol{W}}}_{\rm{{fc}}}+{{\boldsymbol{b}}}_{\rm{{fc}}}.$$

### APEX to predict antimicrobial activity

The hidden representation ***h*** of a peptide generated by the encoder above could be fed into two separate FCNNs that predict species-specific antimicrobial activity or a binary AMP/non-AMP label, respectively. For convenience of hyperparameter tuning, both FCNNs were implemented as a four-layer pyramid-like architecture:

$${{\boldsymbol{h}}}_{\rm{l,s}}={\rm{ReLU}}({\rm{Layer}}{{\_}}{\rm{normalization}}\,({\boldsymbol{h}}_{\rm{l-1}}{\times {\boldsymbol{W}}}_{\rm{l,s}}+{{\boldsymbol{b}}}_{\rm{l,s}})),$$

where $s\in \{{\rm{in{\hbox{-}}house}},{\rm{public}}\}$ denotes the training dataset for the FCNN, $l\in \{{1,2,3,4}\}$ denotes the layer index (note that ${{\boldsymbol{h}}}_{{{0}}}{{=}}{\boldsymbol{h}}$) and ${{\boldsymbol{W}}}_{\rm{l,s}}$ and ${{\boldsymbol{b}}}_{\rm{l,s}}$ are the weight matrix and bias term of the *l*th layer, respectively. At the *l*th layer, a linear transformation was first performed and followed by a layer normalization, a nonlinear transformation using rectified linear unit^48^ (ReLU). In addition, if the *l*th layer is not an output layer, a dropout layer^49^ that randomly set the input value to 0 with probability *P* was added to its output side (we empirically set *P* = 0.1). The output dimensions of hidden layers (that is, ${{\boldsymbol{h}}}_{1,{\rm{s}}}$, ${{\boldsymbol{h}}}_{2,\rm{s}}$ and ${{\boldsymbol{h}}}_{3,\rm{s}}$) were set as $k,\frac{k}{2}$ and $\frac{k}{4}$, respectively. The FCNN that was trained on our in-house data adopted a multitask learning strategy to predict species-specific antimicrobial activity. Suppose there are *g* bacterial species (that is, 34 in our context), the corresponding output ${{\boldsymbol{h}}}_{4,{\rm{in}}{\hbox{-}}{\rm{house}}}$ is a *g*-dimensional vector, in which each element is a predicted antimicrobial activity against a certain bacterial strain. The FCNN that was trained on public AMP data only outputted a scaler value $\in [\mathrm{0,1}{{]}}$ indicating the probability of the input peptide to be antimicrobial.

The loss function for training the FCNN that performed binary classification was binary cross-entropy ${l}_{\rm{BCE}}$. For the other FCNN, the loss function for predicting species-specific antimicrobial activity was the mean squared error:

$${l}_{\rm{MSE}}=\frac{1}{g}\mathop{\sum }\limits_{i=1}^{g}\frac{1}{{d}_{\rm{i}}}\mathop{\sum }\limits_{j=1}^{{d}_{\rm{i}}}{\bf{Mask}}\left(i,j\right)\times{[{{\boldsymbol{h}}}_{{{4}}{{,}}{\rm{in}}{\hbox{-}}{\rm{house}}}(i,j)-{\boldsymbol{y}}(i,j)]}^{2},$$

where *d*_*i*_ denotes the number of training data points for *i*th bacterial strain; ${{\boldsymbol{h}}}_{{{4}}{{,}}{\rm{in}}{\hbox{-}}{\rm{house}}}\left(i,j\right),$ ${\boldsymbol{y}}(i,j)$ and ${\bf{Mask}}\left(i,j\right)$ are the predicted antimicrobial activity, the experimentally validated antimicrobial activity and the binary mask (1 for having antimicrobial activity, and 0 for not tested) between *i*th bacterial strain and *j*th peptide. In addition to these two loss functions on AMP prediction, we further imposed a constraint loss on the weights of output layer in the species-specific AMP prediction FCNN. Given a bacterial distance matrix ${\boldsymbol{P}}{\boldsymbol{\in }}{{\mathbb{R}}}^{g\times g}$ and the weights ${{\boldsymbol{W}}}_{4,{\rm{in}{\hbox{-}}{house}}}$ that we want to regularize, the constraint loss can be written as follows:

$${{\boldsymbol{D}}}_{\rm{task}}=1-{\rm{cosine}{{\_}}{similarity}}({{\boldsymbol{W}}}_{4,{\rm{in}{\hbox{-}}{house}}}{{)}}$$

$${l}_{\rm{multitask}{{\_}}{constrain}}=\frac{1}{2}\mathop{\sum }\limits_{i=1}^{g}\mathop{\sum }\limits_{j=1}^{g}{\boldsymbol{P}}\left({{i}}{{,}}{{j}}\right){\times}{{\boldsymbol{D}}}_{\rm{task}}(i,j),$$

where ${\rm{cosine}}\_{\rm{similarity}}(\bullet)$ calculates the pairwise cosine similarity between two rows of the given input matrix, and matrix ${{\boldsymbol{D}}}_{\rm{task}}{{\in }}{{\mathbb{R}}}^{g\times g}$ stores the pairwise cosine distance between two learnable weights of two predictors. Intuitively, if two tasks are similar, their predictors should also be similar (that is, learnable weights have shorter distances). Adding ${l}_{\rm{multitask}\_\rm{constrain}}$ to the loss function encourages similar bacterial strains to have similar predictors and outputs. Taken together, the final loss function has the following form:

$$L={{l}_{\rm{MSE}}+{\lambda }_{\rm{BCE}}{l}_{\rm{BCE}}+{\lambda }_{\rm{multitask}{{\_}}{constrain}}l}_{\rm{multitask}{{\_}}{constrain}}+{\lambda }_{l2}{\rm{l}}_{2},$$

where *l*_2_ is the L2 regularization for constraining deep learning model complexity, and ${\lambda }_{\rm{BCE}}$, ${\lambda }_{\rm{multitask}\_\rm{constrain}}$ and ${\lambda }_{l2}$ are the weight parameters that balance different types of loss. To train the deep learning models, we used mini-batch training with an Adam optimizer^50^. Specifically, at each iteration, we selected *B* peptides from the in-house dataset and the same number of peptides from public AMP data we curated to perform feed-forwarding pass and back propagation. The training terminated when the procedure iterated the whole in-house dataset 5,000 times. The learning rate of the Adam optimizer was empirically set to 0.0001 and was scheduled to decay 10 times every thousand training epochs. Batch size *B* was empirically set to 128.

### Baseline methods

We compared the prediction performance of APEX to that of several baseline ML models, including elastic net, linear support vector regression, extra-trees regressor, random forest and gradient boosting decision tree. For the baseline models, we represented each peptide sequence by the following features: (1) *k*-mer (that is, frequency of *k*-residue substrings, where *k* = 1, 2 and 3) and (2) 10 peptide properties calculated by modlAMP (version 4.3.0)^51^, including sequence length, molecular weight, sequence charge, charge density, isoelectric point, instability index, aromaticity, aliphatic index, Boman index and hydrophobic ratio. Note that for some of the bacterial strains, the trained Elastic Net outputted a constant prediction regardless of the peptide inputs. In this case, the Pearson and Spearman correlations could not be calculated, and we used 0 as pseudo correlation.

### Hyperparameter tuning, model evaluation and ensemble learning

We conducted a fivefold CV on the CV set to select the hyperparameters for deep learning and baseline models. Specifically, the fivefold CV split the whole dataset evenly into five groups. At each time, one group was selected as the test dataset, while the rest were used for ML model training. We used averaged *R*^2^, Pearson correlation coefficient and Spearman’s rank correlation coefficient under fivefold CV to evaluate the prediction performance on the test set. Grid search was used to find the best hyperparameters (see Supplementary Tables 1–4 for the hyperparameter range we searched). Hyperparameters were ranked by the averaged *R*^2^ under CV. For baseline methods, we determined the best hyperparameters to be those resulting in the highest *R*^2^ and trained the ML models with the selected hyperparameters. The trained models were then evaluated on the independent dataset. For APEX, we adopted an ensemble learning strategy. Specifically, we averaged the prediction results from the top eight APEX models. After plotting the prediction performances versus the number of deep learning models involved in the ensemble learning (Supplementary Figs. 8–10), we observed that the elbow region (that is, the area where the curve becomes smaller) was around 5–9 APEX models. After this step, improvement on prediction performance gradually became negligible. This observation led to the conclusion that we should average prediction results from no more than nine APEX models. We decided to select eight APEX models for ensemble learning, given our computational resources (that is, eight graphics processing units were available). To counter the potential stochastic behaviour during mini-batch training and to make prediction results more robust, we trained five copies of an APEX model with the same hyperparameters under different random seeds. In total, we trained 40 APEX models (that is, 8 different hyperparameters × 5 different random seeds) and used the averaged predictions on the independent dataset for prediction performance evaluation. After the performance evaluation, we retrained the 40 APEX models on the entire in-house dataset and used the averaged antimicrobial activity prediction values from the trained models to discover EP sequences within extinct organisms.

### Selectivity score

As APEX was designed to predict species-specific antimicrobial activity, we defined the following two selectivity scores that quantify the peptides’ ability to specifically target Gram-positive or Gram-negative bacteria:

$$\begin{array}{l}{\rm{Gram}}-{\rm{positive}}\,{\rm{selectivity}}\,{\rm{score}}\\=\frac{\rm{Median}\,\rm{MIC}(\rm{Gram}-\rm{positive}\,\rm{pathogens})}{\rm{Median}\,\rm{MIC}(\rm{Gram}-\rm{negative}\,\rm{pathogens})},\end{array}$$

$$\begin{array}{l}{\rm{Gram}}-{\rm{negative}}\,{\rm{selectivity}}\,{\rm{score}}\\=\frac{\rm{Median}\,\rm{MIC}(\rm{Gram}-\rm{negative}\,\rm{pathogens})}{\rm{Median}\,\rm{MIC}(\rm{Gram}-\rm{positive}\,\rm{pathogens})},\end{array}$$

where Median MIC(·) calculates the median value from a given input list. The input list consisted of the Gram-positive pathogens *S. aureus* ATCC 12600, methicillin-resistant *S. aureus* ATCC BAA-1556, vancomycin-resistant *E. faecalis* ATCC 700802 and vancomycin-resistant *E. faecium* ATCC 700221 and the Gram-negative pathogens *P. aeruginosa* PAO1, *P. aeruginosa* PA14, *E. coli* ATCC11775, *E. coli* AIC221, *E. coli* AIC222, *K. pneumoniae* ATCC 13883 and *A. baumannii* ATCC 19606. A selectivity score of <1.0 means that the median MIC of the target bacteria (numerator term) is smaller than that of the off-target bacteria (denominator term), yielding a selective peptide sequence. Thus, the closer to 0, the better is the selective activity towards the specific bacterial targets.

### Sequence similarity score

Given two peptide sequences *i* and *j*, we used the Smith–Waterman algorithm^20^ to calculate their sequence alignment score ${{\rm{SW}}(i,j)}$. The sequence similarity score between these two peptides was defined as the normalized alignment score: $\frac{{\rm{SW}}(i,\;j)}{\sqrt{{\rm{SW}}\left(i,i\right) \times {{\rm{SW}}(j,\;j)}}}\in [\mathrm{0,1}]$. A higher score reflects higher sequence similarity between two peptides than a lower score.

### Antibiotic peptide screening and selection

Given the proteome of extinct organisms, we considered as EP sequence substrings ranging from 8 to 50 amino acid residues. In total, paleoproteome mining yielded 10,311,899 unique molecules from extinct proteomes, of which 771,431 sequences came from secreted proteins. EPs from secreted proteins would have had a higher likelihood of encountering bacterial cells, which are mostly found outside host cells, compared to sequences from non-secreted proteins. Nevertheless, most EPs came from non-secreted proteins and consequently represented a more abundant peptide source. We hypothesized that these non-secreted proteins might also contain antibiotic-like substrings. Therefore, in this work, we selected, synthesized and validated EPs originating from both secreted and non-secreted proteins.

As we used 40 APEX models for activity prediction (that is, ensemble APEX v2 or APEX in the main text), we averaged the prediction results from these 40 models to provide a final predictive output for each peptide. Based on these APEX predictions, we used multiple criteria to select EPs from our predictions for downstream validation. First, we mainly focused on EPs that could target a subset of the eleven pathogens that were listed in the ‘Selectivity score section’ (that is, *E. coli* ATCC 11775, *P. aeruginosa* PAO1, *P. aeruginosa* PA14, *S. aureus* ATCC 12600, *E. coli* AIC221, *E. coli* AIC222, *K. pneumoniae* ATCC 13883, *A. baumannii* ATCC 19606, methicillin-resistant *S. aureus* ATCC BAA-1556, vancomycin-resistant *E. faecalis* ATCC 700802 and vancomycin-resistant *E. faecium* ATCC 700221). Given the entire peptide list, we first generated two EP lists; one with sequences predicted to selectively target Gram-positive and another with sequences predicted to target Gram-negative pathogens. To this end, the peptides in these lists were ranked increasingly by the Gram-positive selectivity score and the Gram-negative selectivity score, respectively. We then generated another peptide list by ranking the peptides based on their median MIC predictions for the 11 pathogens of interest, indicating the level of broad-spectrum antimicrobial activity of the peptides.

As a result, three lists were generated for EPs derived from non-secreted proteins, and another three lists were generated for EPs derived from secreted proteins, corresponding in each case to the following: (a) EPs predicted to selectively target Gram-positive bacteria, (b) EPs predicted to selectively target Gram-negative bacteria and (c) EPs predicted to have broad-spectrum activity. The six lists were filtered by the following criteria:

1. The selected EP sequences were restricted to a range between 8 and 30 residues long. Selection of shorter sequences within this range facilitated chemical synthesis.
2. Sequences that were present in the modern human proteome were excluded.
3. Sequences that were present in our in-house dataset, including both natural and synthetic sequences, were excluded.
4. EP candidates with more than 75% sequence similarity to peptides from DBAASP were considered analogues or derivatives of such peptides and were excluded.
5. If two candidate EPs presented more than 75% sequence similarity, the one ranked higher by APEX (that is, lower median MIC or better selectivity score) was kept, and the analogue/derivative was excluded, encouraging diversity of the identified sequences.
6. Six lists were generated, each containing up to 1,000 top candidate EP sequences. The lists contained peptide sequences that were predicted to be broad spectrum, selective towards Gram-negative and selective for Gram-positive bacteria. These sequences derived from non-secreted proteins or secreted proteins were present in extinct proteomes. The broad-spectrum lists were ranked by median MIC, while the selectivity lists were ranked by the selectivity score.
7. For the broad-spectrum lists, candidate EPs with median MIC predictions of ≥80 μmol l^−1^ were excluded as they were deemed inactive. For peptide lists selective towards Gram-positive or Gram-negative bacteria, sequences with selectivity scores ≥0.75 were excluded as they were considered not sufficiently selective.

After applying the seventh filter, a total of 3,784 unique candidate EPs were left from the initial 10,311,899 sequences derived from extinct proteomes. Note that before selecting the peptides for synthesis, we grouped the candidate EPs by their source organisms. We selected the top one to five EPs (that is, more active or selective depending on which list they were from) taking into account species and sequence diversity (Supplementary Dataset 2). In summary, 21 EPs from secreted extinct proteins were selected for synthesis and subsequent experimental validation: 4 of these were predicted to selectively target Gram-positive bacteria, 10 to selectively target Gram-negative bacteria and 7 to have broad-spectrum activity. Another set of 48 EPs from non-secreted extinct proteins was also selected for downstream experimental validation: 19 that were predicted to selectively target Gram-positive bacteria, 10 that were predicted to selectively target Gram-negative bacteria and 19 that were predicted to show broad-spectrum activity. Therefore, a total of 69 peptides (21 from secreted extinct proteins and 48 from non-secreted extinct proteins) out of the 3,784 sequences were selected, synthesized and validated experimentally.

### Classification guidelines to identify AEPs

As de-extinct sequences have not been previously identified, we developed our own classification guidelines to determine whether a particular peptide sequence qualified as truly de-extinct, that is, not present in available proteomic data from extant organisms. As EP sequences from the proteomes of extinct organisms may also be found in modern organisms, we used the following procedure to classify an EP sequence as an AEP: First, we accessed the NCBI taxonomy browser (https://www.ncbi.nlm.nih.gov/Taxonomy/taxonomyhome.html/index.cgi?chapter=extinct, access time: December, 2022) to get the most up-to-date taxonomy IDs of extinct organisms, and this was our sole source of extinct proteins. Next, we created a protein sequence set containing all possible organism sources by downloading protein sequences and their corresponding taxonomy IDs from Reviewed (Swiss-Prot), Unreviewed (TrEMBL) and Isoform sequences at UniProt^52^ (https://www.uniprot.org/help/downloads). This constituted our source of extant proteins. We excluded from the protein sequence set those protein sequences whose taxonomy IDs belonged to extinct organisms. We labelled the resulting set as ‘extant protein set’ as it contained protein sequences from extant organisms. If a given EP sequence was not present in any protein sequence from the available extant protein set, we defined it as an AEP. Otherwise, the EP was considered as a MEP.

### Peptide synthesis

All peptides were synthesized by solid-phase peptide synthesis using the Fmoc strategy and purchased from AAPPTec.

### Bacterial strains and growth conditions used in the experiments

The following Gram-negative bacteria were used in our study: *A. baumannii* ATCC 19606, *E. coli* ATCC 11775, *E. coli* AIC221 (*E. coli* MG1655 phnE_2::FRT), *E. coli* AIC222 (*E. coli* MG1655 pmrA53 phnE_2::FRT (colistin resistant)), *K. pneumoniae* ATCC 13883, *P. aeruginosa* PAO1 and *P. aeruginosa* PA14. The following Gram-positive bacteria were also used in our study: *S. aureus* ATCC 12600, *S. aureus* ATCC BAA-1556 (methicillin-resistant strain), *E. faecalis* ATCC 700802 (vancomycin-resistant strain) and *E. faecium* ATCC 700221 (vancomycin-resistant strain). Bacteria were grown from frozen stocks and plated on Luria–Bertani (LB) or *Pseudomonas* isolation agar plates (*P. aeruginosa* strains) and incubated overnight at 37 °C. After the incubation period, a single colony was transferred to 5 ml of LB medium, and cultures were incubated overnight (16 h) at 37 °C. The following day, an inoculum was prepared by diluting the overnight cultures 1:100 in 5 ml of the respective media and incubating them at 37 °C until bacteria reached logarithmic phase (OD_600_ = 0.3–0.5).

### Antibacterial assays

The in vitro antimicrobial activity of the peptides was assessed by using the broth microdilution assay^36^. MIC values of the peptides were determined with an initial inoculum of 2 × 10^6^ cells ml^−1^ in LB in microtitre 96-well flat-bottom transparent plates. Aqueous solutions of the peptides were added to the plate at concentrations ranging from 1 to 64 μmol l^−1^. The lowest concentration of peptide that inhibited 100% of the visible growth of bacteria was established as the MIC value in an experiment of 20 h of exposure at 37 °C. The optical density of the plates was measured at 600 nm using a spectrophotometer. All assays were done as three biological replicates.

### Outer membrane permeabilization assays

The membrane permeability of the peptides was determined by using the NPN uptake assay^16^. NPN is a hydrophobic fluorescent dye that does not readily permeate the bacterial outer membrane. However, when the membrane integrity is compromised, NPN can enter the cell and bind to the bacterial membrane lipids. This causes the dye to show a strong fluorescence. *A. baumannii* ATCC19606 and *P. aeruginosa* PAO1 were grown (OD_600_ = 0.4), centrifuged (10,000 r.p.m. at 4 °C for 10 min), washed and resuspended in buffer (5 mmol l^−1^ HEPES, 5 mmol l^−1^ glucose, pH 7.4). NPN solution (4 μl at the working concentration of 10 mmol l^−1^ after dilution) was added to 100 μl of the bacterial solution in a white 96-well plate. The fluorescence was recorded at *λ*_ex_ = 350 nm and *λ*_em_ = 420 nm. Aqueous solutions of the peptides (100 μl final volume at their MIC against the strain of interest) were added to a white 96-well plate, and fluorescence was recorded for 45 min after no further increase in fluorescence was observed. All assays were done as three biological replicates. The relative fluorescence values were calculated for the entire course of the experiment using non-linear fitting, and the untreated control (buffer + bacteria + fluorescent dye) as baseline. The following equation was applied to show the percentage difference between the fluorescence of the untreated control (baseline) and the sample:

$${\rm{{Percentage}\,{difference}}}=\frac{100\times ({\rm{fluorescence}_{sample}}-{\rm{baseline}})}{\rm{baseline}}$$

### Cytoplasmic membrane depolarization assays

The depolarization of the bacterial cytoplasmic membrane was determined by fluorescence measurements of the membrane potential-sensitive dye, DiSC_3_-5 (ref. ^16^). Briefly, *A. baumannii* ATCC 19606 and *P. aeruginosa* PAO1 were grown at 37 °C until mid-log phase (OD_600_ = 0.5). The cells were then centrifuged using the same conditions described for the NPN uptake assays, washed twice with washing buffer containing 20 mmol l^−1^ glucose and 5 mmol l^−1^ HEPES (pH 7.2). The cells were diluted 1:10 (OD_600_ = 0.05) in a buffer containing 0.1 mol l^−1^ KCl, 20 mmol l^−1^ glucose and 5 mmol l^−1^ HEPES (pH 7.2). About 100 μl of bacterial solution was then incubated for 15 min with 20 nmol l^−1^ of DiSC_3_-5 until the fluorescence reached a plateau, that is, the dye was fully internalized into the bacterial membrane. Transmembrane potential changes were monitored by observing the difference in the fluorescence emission intensity of DiSC_3_-5 (*λ*_ex_ = 622 nm, *λ*_em_ = 670 nm), after the addition of 100 μl of peptide aqueous solution at its MIC. All assays were performed in three biological replicates. The relative fluorescence values were calculated for the course of the experiment using non-linear fitting, and the untreated control (buffer + bacteria + fluorescent dye) served as baseline. The following equation was applied to show the percentage difference between the fluorescence of the untreated control (baseline) and the sample:

$${\rm{{Percentage}\,{difference}}}=\frac{100 \times (\rm{{fluorescence}_{sample}}-{baseline})}{\rm{baseline}}$$

### Synergy between antibiotic molecules from extinct organisms

*P. aeruginosa* PAO1 and *A. baumannii* ATCC 19606 were used to assess the synergistic interactions of peptides derived from the same organisms using checkerboard assays. These bacterial pathogens were selected due to their resistance to antibiotics. The most active de-extinct EPs against *P. aeruginosa* PAO1 and *A. baumannii* ATCC 19606 were orthogonally diluted using the microdilution technique to concentrations ranging from 4 times MIC to 0.0625 times MIC. Plates were incubated for 20 h at 37 °C. All assays were done in three biological replicates.

### Eukaryotic cell culture conditions

HEK293T cells were obtained from the American Type Culture Collection (CRL-3216). The cells were cultured in high-glucose Dulbecco’s modified Eagle’s medium supplemented with 1% penicillin and streptomycin (antibiotics) and 10% fetal bovine serum and grown at 37 °C in a humidified atmosphere containing 5% CO_2_.

### Cytotoxicity assays

One day before the experiment, an aliquot of 100 μl of the cells at 50,000 cells per ml was seeded into each well of the cell-treated 96-well plates used in the experiment (that is, 5,000 cells per well). The attached HEK293T cells were then exposed to increasing concentrations of the peptides (8–128 μmol l^−1^) for 24 h. After the incubation period, we performed the 3-(4,5-dimethylthiazol-2-yl)-2,5-diphenyltetrazolium bromide tetrazolium reduction assay (MTT assay)^53^. The MTT reagent was dissolved at 0.5 mg ml^−1^ in medium without phenol red and was used to replace cell culture supernatants containing the peptides (100 μl per well), and the samples were incubated for 4 h at 37 °C in a humidified atmosphere containing 5% CO_2_ yielding the insoluble formazan salt. The resulting salts were then resuspended in hydrochloric acid (0.04 mol l^−1^) in anhydrous isopropanol and quantified by spectrophotometric measurements of absorbance at 570 nm. All assays were done as three biological replicates.

### Resistance to proteolytic degradation assays

To assess the resistance of EPs to proteolysis, we incubated them in human serum^54^. The following five lead compounds were exposed to 25% human serum in water for 6 h at 37 °C: mammuthusin-2, hydrodamin-1, megalocerin-1, elephasin-2 and mylodonin-2, each at 3 mg ml^−1^. About 100 μl aliquots were collected after 0, 0.5, 1, 3 and 6 h, and 10 μl of 100% trifluoroacetic acid was added to each sample to induce protein precipitation and incubated for 10 min on ice (at ~4 °C). Samples were then processed in a Waters Acquity UPLCMS equipped with a photodiode array detector (190–400 nm data collection) and a Waters TQD triple quadrupole MSMS, with 5 μl injections. The column used was a Waters Acquity UPLC HSS C_18_, 1.8 μm (2.1 mm × 50 mm). The mobile phases used were A (100% water with 0.1%, *v*/*v*, formic acid) and B (100% acetonitrile with 0.1%, *v*/*v*, formic acid), Fisher optima grades. The solvent gradient used is described in detail in Supplementary Table 18. Measurements were made by ionization ESI +/− simultaneous over *m*/*z* 100–2,000 Da. The percentage of remaining peptide was calculated by integrating the area under the curve related to the peptide at time point 0. Experiments were performed in three independent replicates.

### Circular dichroism assays

Circular dichroism assays were performed at the University of Pennsylvania’s Biological Chemistry Resource Center using a J1500 circular dichroism spectropolarimeter (Jasco). The circular dichroism spectra represent an average of three accumulations at 25 °C obtained using a quartz cuvette with an optical path length of 1.0 mm. The experiments covered a wavelength range from 260 to 190 nm at a rate of 50 nm min^−1^ and a bandwidth of 0.5 nm. The concentration of all peptides tested was kept at 50 μmol l^−1^, and the measurements were performed in a mixture of water and trifluoroethanol in a 3:2 ratio. A Fourier transform filter was applied to minimize background effects. Secondary structure fraction values were calculated using the single spectra analysis tool on the server BeStSel (version from March 27, 2022)^55^.

### Skin abscess infection mouse model

*A. baumannii* ATCC 19606 cells were grown in LB medium to an OD_600_ = 0.5. Cells were washed twice with sterile phosphate-buffered saline (PBS) (pH 7.4, 13,000 r.p.m. for 2 min) and resuspended to a final concentration of 10^6^ (*A. baumannii* cells) colony-forming units per millilitre (c.f.u. ml^−1^). Six-week-old female CD-1 mice from Charles River (stock number 18679700-022) were anaesthetized with isoflurane, and their backs were sterilized and shaved. A superficial linear skin abrasion was made with a needle to damage the stratum corneum and upper layer of the epidermis. An aliquot of 20 μl containing the bacterial load resuspended in PBS was inoculated over the scratched area. Two hours after the infection, peptides diluted in water at their MIC value were administered to the infected area, and the untreated mice were inoculated with the same volume of deionized water. Mice were euthanized, and the area of scarified skin was excised 2 days and 4 days post infection, homogenized using a bead beater for 20 min (25 Hz) and then serially diluted tenfold for quantification of colony-forming units. This quantitative method was used as it has been established to accurately reflect the number of bacteria present in a given infected area. MacConkey agar plates were used as *A. baumannii* colonies appear purple in this medium and can easily be distinguished from other strains. The experiments were performed with six mice per group. All experiments were performed blindly, and no animal subjects were excluded from the analysis. The skin abscess infection mouse model was approved by the University Laboratory Animal Resources from the University of Pennsylvania (protocol 806763). Statistical significance was determined using one-way analysis of variance (ANOVA) followed by Dunnett’s test in log_10_-transformed data to mitigate the effect of outliers; *P* values are presented for each group, with all groups being compared to the untreated control group.

### Thigh infection mouse model

Six-week-old female CD-1 mice from Charles River (stock number 18679700-022) were rendered neutropenic by two doses of cyclophosphamide (150 mg kg^−1^) applied intraperitoneally with an interval of 72 h. One day after the last dose of cyclophosphamide, the mice were injected intramuscularly in their right thigh with a bacterial load of 10^6^ c.f.u. ml^−1^ of *A. baumannii* ATCC 19606 cells. The bacteria had been grown in LB broth, washed twice with PBS (pH 7.4), and resuspended to the desired concentration. Two hours after bacterial injection, peptides resuspended in water were administered intraperitoneally, and the untreated mice were inoculated with the same volume of deionized water. Before each injection, mice were anaesthetized with isoflurane and monitored for respiratory rate and pedal reflexes. Next, we monitored the establishment of the infection and euthanized the mice. The infected area was excised 2 days and 4 days post infection, homogenized using a bead beater for 20 min (25 Hz), and then serially diluted tenfold for quantification of colony-forming units in MacConkey agar plates. The experiments were performed with six mice per group. All experiments were performed blindly, and no animal subjects were excluded from the analysis. The thigh infection mouse model was approved by the University Laboratory Animal Resources from the University of Pennsylvania (protocol 807055). Statistical significance was determined using one-way ANOVA followed by Dunnett’s test in log_10_-transformed data to mitigate the effect of outliers; *P* values are presented for each group, with all groups being compared to the untreated control group.

### Statistical analyses

Unless otherwise stated, all assays were performed in three independent biological replicates as indicated in each figure legend and Methods sections. The cytotoxic activities values were estimated using non-linear regression based on the range of concentrations screened and were shown as the values that cause lysis of 50% of the cells in the experiment. Two technical replicates were performed in the cytotoxicity assays within each of the three biological replicates.

In the mouse experiments, the statistical significance was determined using one-way ANOVA followed by Dunnett’s test. All the *P* values are shown for each of the groups, and all groups were compared to the untreated control group. The solid line inside each box represents the mean value obtained for each group.

All calculation and statistical analyses of the experimental and computational data were conducted using GraphPad Prism v.10.0.2 and Python, respectively. Statistical significance between different groups was calculated using the tests indicated in each figure legend. No statistical methods were used to predetermine sample size.

### Reporting summary

Further information on research design is available in the Nature Portfolio Reporting Summary linked to this article.

## Supplementary information

Supplementary Information Supplementary Figures and Tables.

Reporting Summary

Supplementary Dataset 1 List of EPs predicted by APEX to have a median MIC ≤ 80 μmol l^−1^.

Supplementary Dataset 2 Information of EPs identified by APEX and validated experimentally.

Supplementary Dataset 3 Information of EPs identified by a scoring function and validated experimentally.

## Source data

Source Data Fig. 6 Source data.

## Data Availability

The main data supporting the results in this study are available within the paper and its Supplementary Information. Proteomes mined in this study are publicly available from NCBI and UniProt (https://www.ncbi.nlm.nih.gov/Taxonomy/taxonomyhome.html/index.cgi?chapter=extinct and https://www.uniprot.org/help/downloads). All data generated in this study, including Source Data for the figures, are available from the corresponding author on reasonable request. Source data are provided with this paper.

## Code availability

The custom code for the pretrained APEX models is available at GitLab (https://gitlab.com/machine-biology-group-public/apex).
