# Supplementary material for: Deep-learning-enabled antibiotic discovery through molecular de-extinction
Source: Nat Biomed Eng. 2024 Jun 11;8(7):854–71. doi: 10.1038/s41551-024-01201-x (PMC11310081; doi:10.1038/s41551-024-01201-x)
Supplement: Supplementary file 1 — Supplementary Figures and Tables. [file 41551_2024_1201_MOESM1_ESM.pdf]

nature biomedical engineering

Supplementary information

https://doi.org/10.1038/s41551-024-01201-x

Deep-learning-enabled antibiotic discovery through molecular de-extinction

In the format provided by the authors and unedited

Contents

Abbreviations

Supplementary Figs. 1 to 52

Supplementary Tables 1 to 18

References

Description of Supplementary Datasets 1 to 3

Abbreviations

EP: encrypted peptides (encrypted peptides are generally defined as peptide sequences embedded within proteins)

MEPs: modern EPs, EPs found in both extinct and extant organisms

AEPs: archaic EPs, EPs found only in extinct organisms

AMPs: antimicrobial peptides from publicly available databases

DL: deep learning

APEX: Antibiotic Peptide de-EXtinction model.

Supplementary figures

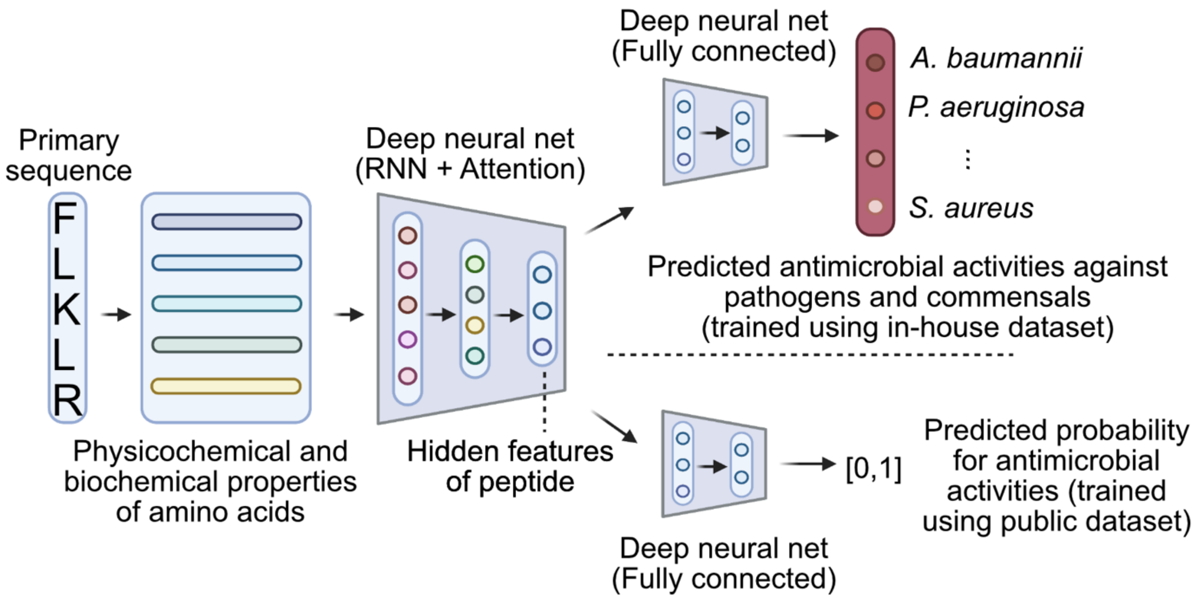

Supplementary Fig. 1 | Schematic illustration of APEX. APEX utilized a hybrid of recurrent and attention neural networks to extract peptide sequence information. Extracted hidden features for peptides from in-house or public datasets were processed by two fully connected neural networks to predict species-specific antimicrobial activities (i.e., a multi-output regression task) or general antimicrobial or not (i.e., a binary classification task), respectively. We adopted this multitask framework to accurately predict whether a given peptide sequence was likely to have antimicrobial activity. This figure was created with BioRender.com.

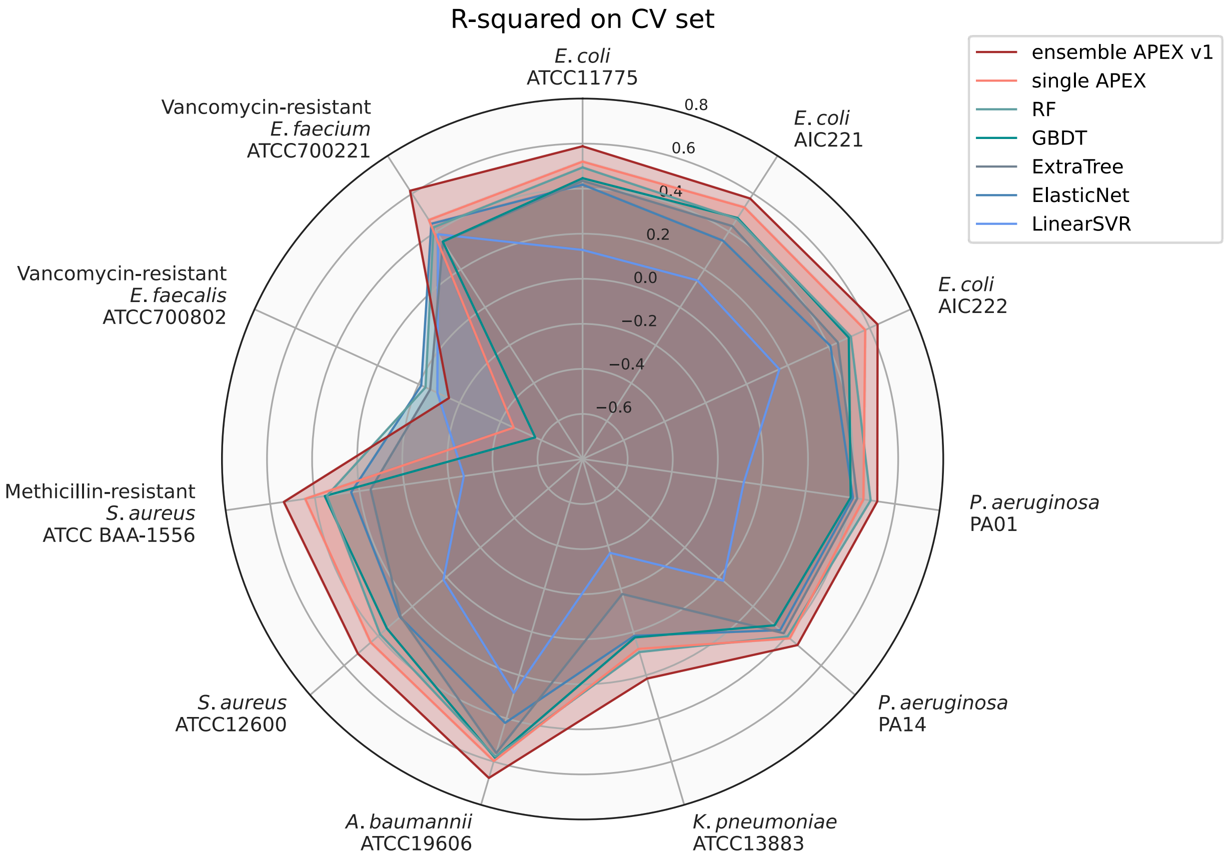

Supplementary Fig. 2 | R-squared scores of various ML models on cross-validation (CV) set. We divided our in-house peptide dataset into a CV set for hyperparameter tuning and ML model selection, and an independent dataset to evaluate the prediction performance. The figure shows the average species-specific prediction performance of 5-fold CV in terms of R-squared for various ML models on the CV set. The radius reflects the R-squared value. RF: random forest; GBDT: gradient boosting decision tree; ExtraTree: extra-tree regressor; ElasticNet: elastic net; LinearSVR: linear support vector regression.

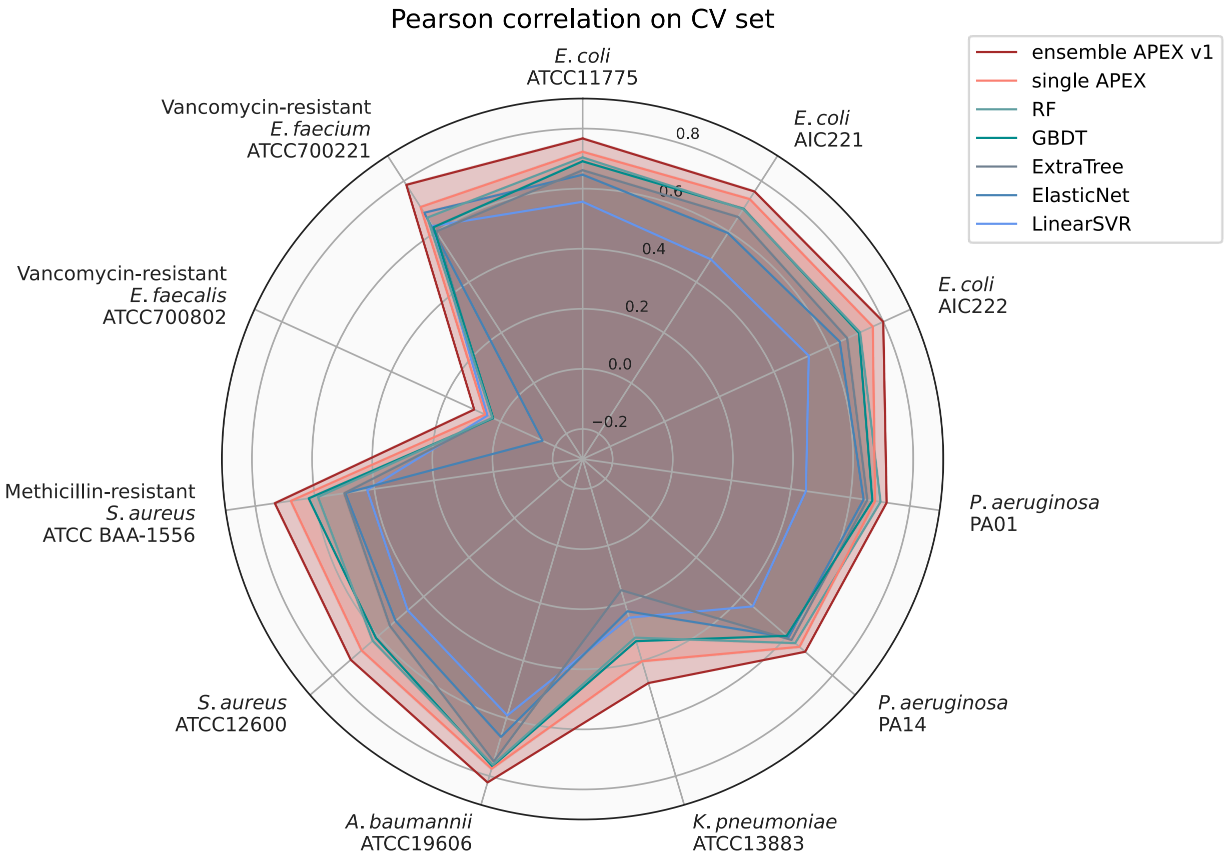

Supplementary Fig. 3 | Pearson correlation scores of various ML models on cross-validation (CV) set. We divided our in-house peptide dataset into a CV set for hyperparameter tuning and ML model selection, and an independent dataset to evaluate the prediction performance. The figure shows the average species-specific prediction performance of 5-fold CV in terms of Pearson correlation for various ML models on the CV set. The radius reflects the Pearson correlation value. RF: random forest; GBDT: gradient boosting decision tree; ExtraTree: extra-tree regressor; ElasticNet: elastic net; LinearSVR: linear support vector regression.

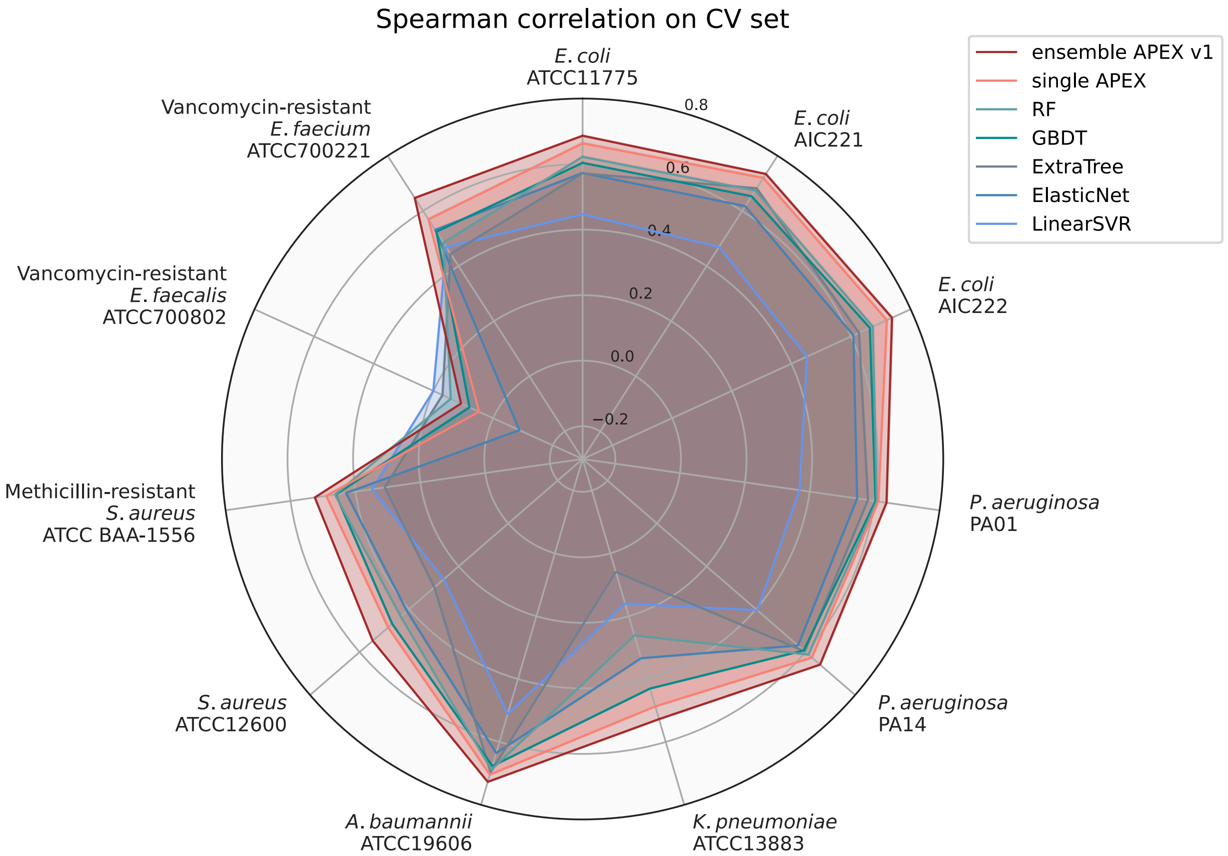

Supplementary Fig. 4 l Spearman correlation scores of various ML models on cross-validation (CV) set. We divided our in-house peptide dataset into a CV set for hyperparameter tuning and ML model selection, and an independent dataset to evaluate the prediction performance. The figure shows the average species-specific prediction performance of 5-fold CV in terms of Spearman correlation for various ML models on the CV set. The radius reflects the Spearman correlation value. RF: random forest; GBDT: gradient boosting decision tree; ExtraTree: extra-tree regressor; ElasticNet: elastic net; LinearSVR: linear support vector regression.

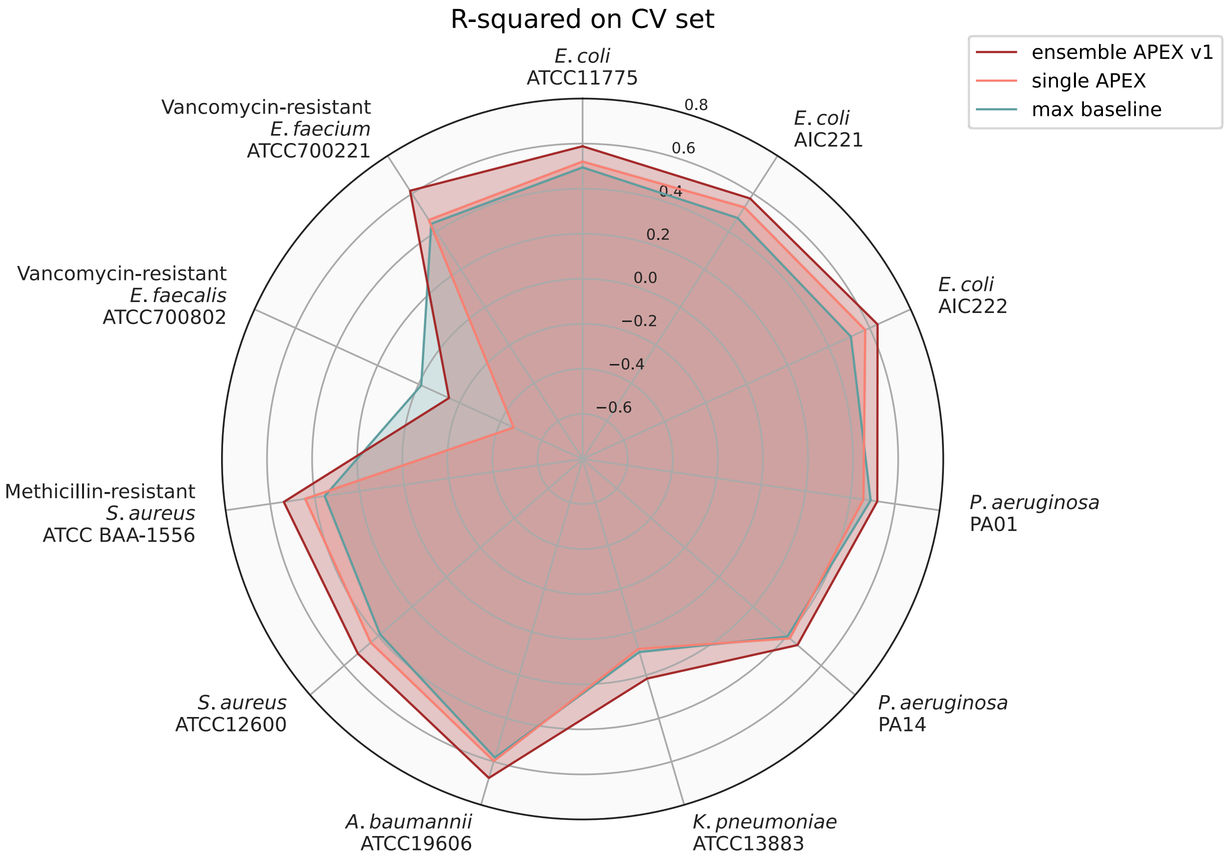

Supplementary Fig. 5 | R-squared scores of various ML models on cross-validation (CV) set. We divided our in-house peptide dataset into a CV set for hyperparameter tuning and ML model selection, and an independent dataset to evaluate the prediction performance. The figure shows the average species-specific prediction performance of 5-fold CV in terms of R-squared for APEX models and maximum performance from baseline ML models on the CV set. The radius reflects the R-squared value. Max baseline denotes the highest R-squared values from baseline ML models, including elastic net, linear support vector regression, extra-trees regressor, random forest and gradient boosting decision tree.

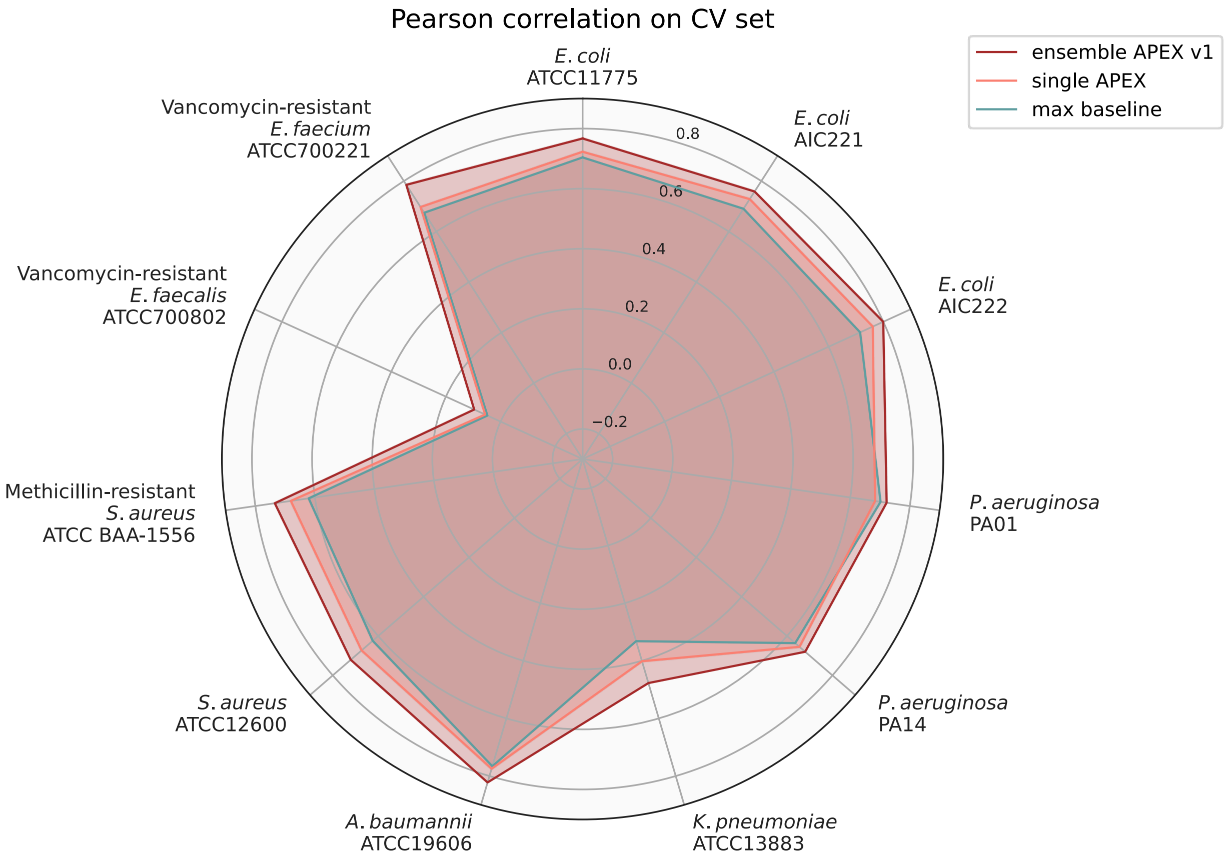

Supplementary Fig. 6 | Pearson correlation scores of various ML models on CV set. We divided our in-house peptide dataset into a CV set for hyperparameter tuning and ML model selection, and an independent dataset to evaluate the prediction performance. The figure shows the average species-specific prediction performance of 5-fold CV in terms of Pearson correlation for APEX models and maximum performance from baseline ML models on the CV set. The radius reflects the Pearson correlation value. Max baseline denotes the highest Pearson correlation values from baseline ML models, including elastic net, linear support vector regression, extra-trees regressor, random forest and gradient boosting decision tree.

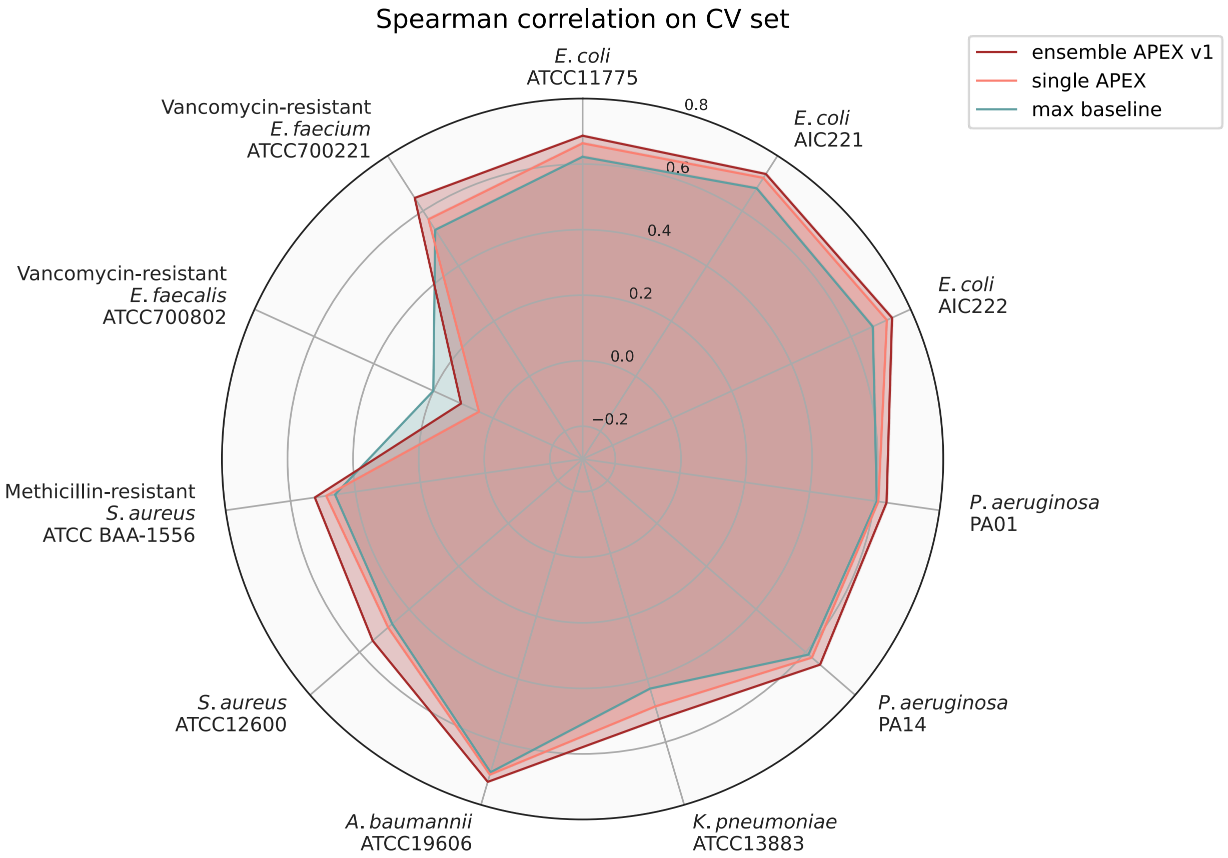

Supplementary Fig. 7 | Spearman correlation scores of various ML models on CV set. We divided our in-house peptide dataset into a CV set for hyperparameter tuning and ML model selection, and an independent dataset to evaluate the prediction performance. The figure shows the average species-specific prediction performance of 5-fold CV in terms of Spearman correlation for APEX models and maximum performance from baseline ML models on the CV set. The radius reflects the Spearman correlation value. Max baseline denotes the highest Spearman correlation values from baseline ML models, including elastic net, linear support vector regression, extra-trees regressor, random forest and gradient boosting decision tree.

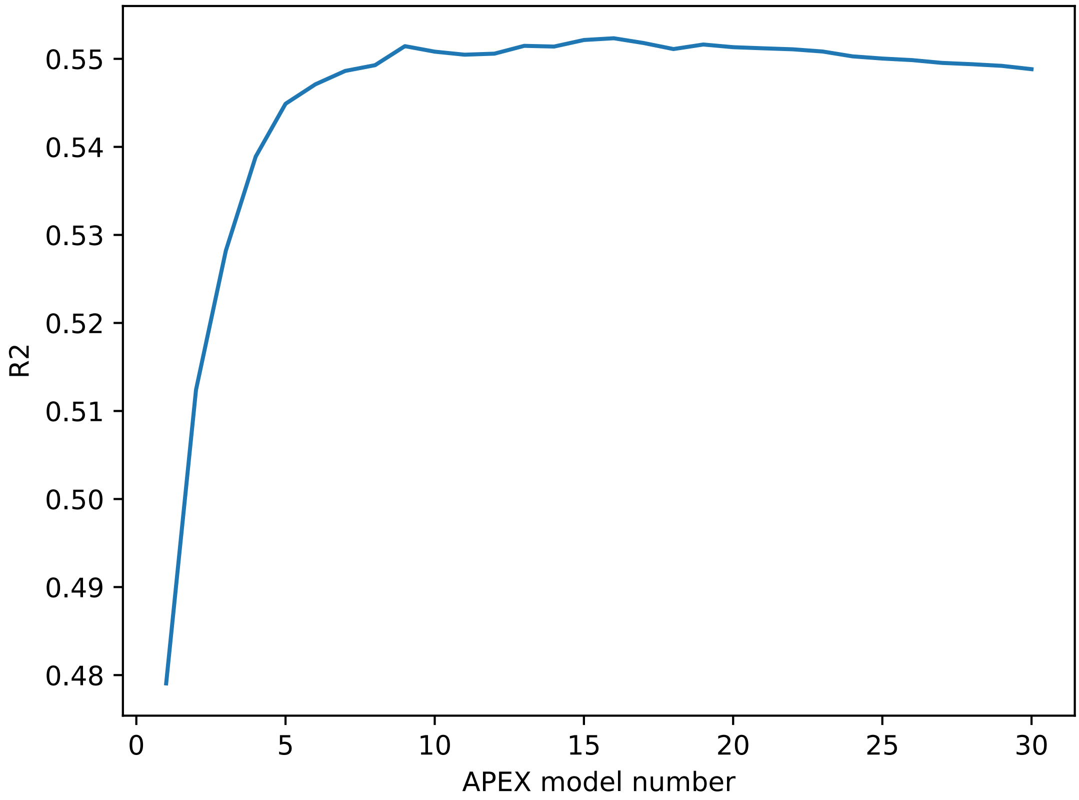

Supplementary Fig. 8 | Relationship between R-squared and the number of APEX models used in ensemble learning on the CV set. We divided our in-house peptide dataset into a CV set for hyperparameter tuning and ML model selection, and an independent dataset to evaluate prediction performance. On the CV set, we trained several APEX models under different deep learning architectures and training strategies. We ranked the APEX models by the averaged R-squared to predict species-specific antimicrobial activity. We averaged the predictions from the top-ranked models to create the ensemble APEX to improve antibiotic prediction performance. The figure shows the relationship between the averaged R-squared for species-specific antimicrobial activity prediction and the number of APEX models being averaged.

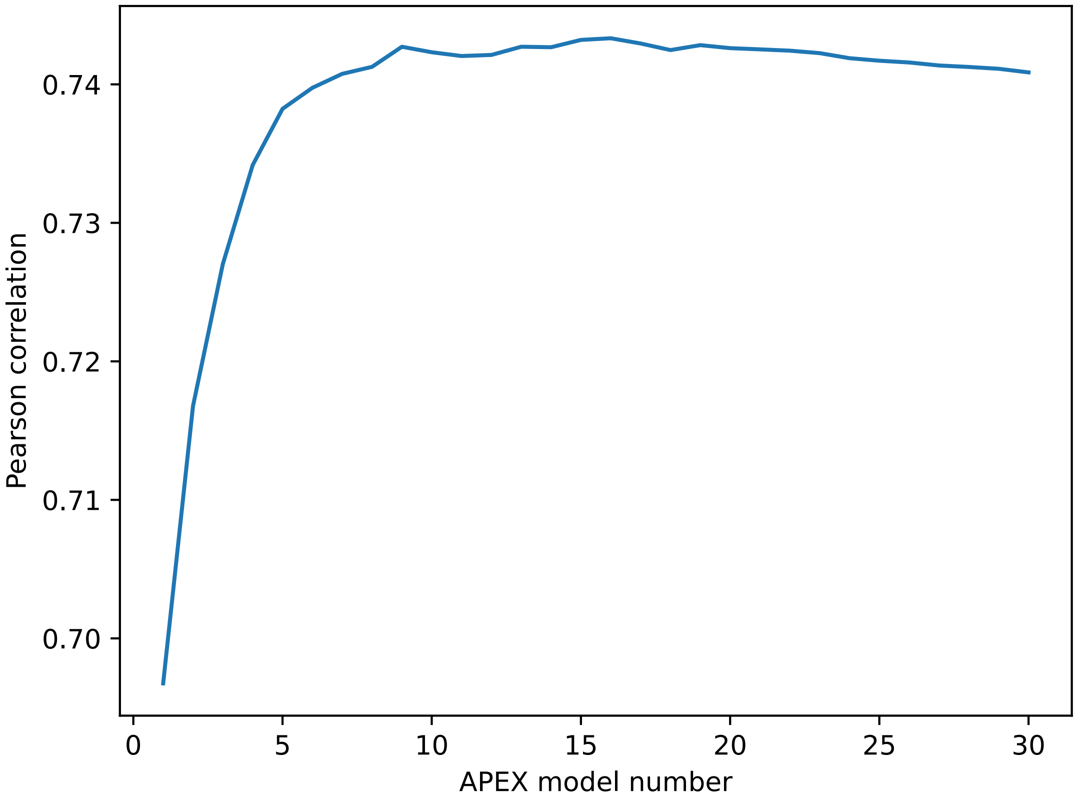

Supplementary Fig. 9 | Relationship between Pearson correlation and the number of APEX models used in ensemble learning on the CV set. We divided our in-house peptide dataset into a CV set for hyperparameter tuning and ML model selection, and an independent dataset to evaluate prediction performance. On CV set, we trained the APEX models under different deep learning architectures and training strategies, then ranked them by the averaged R-squared for species-specific antimicrobial activity prediction. To improve antibiotic prediction performance, we averaged the predictions from the top-ranked models creating ensemble APEX. The figure shows the relationship between the averaged Pearson correlation for species-specific antimicrobial activity prediction and the number of APEX models being averaged.

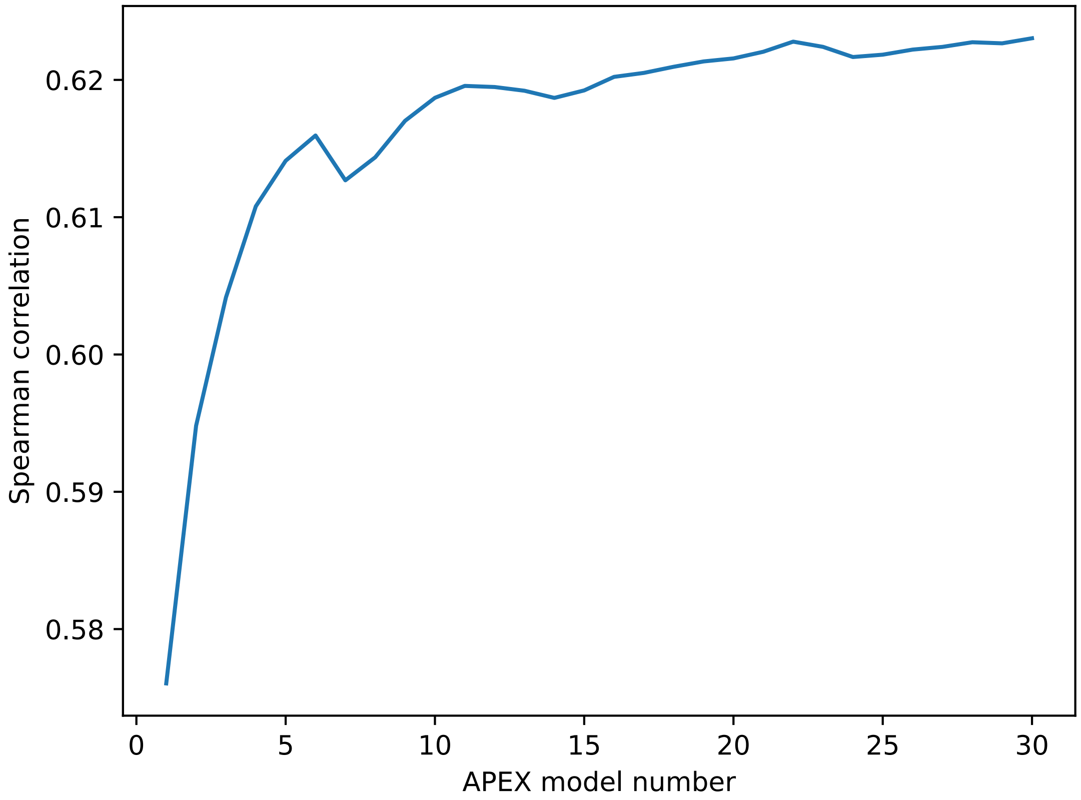

Supplementary Fig. 10 | Relationship between Spearman correlation and the number of APEX models used in ensemble learning on CV set. We divided our in-house peptide dataset into a CV set for hyperparameter tuning and ML model selection, and an independent dataset to evaluate prediction performance. On CV set, we trained APEX models under different deep learning architectures and training strategies. We ranked the APEX models by the averaged R-squared for species-specific antimicrobial activity prediction. To improve antibiotic prediction performance, we averaged the predictions from the top-ranked models to create ensemble APEX to improve antibiotic prediction performance. The figure shows the relationship between averaged Spearman correlation for species-specific antimicrobial activity prediction and the number of APEX models being averaged.

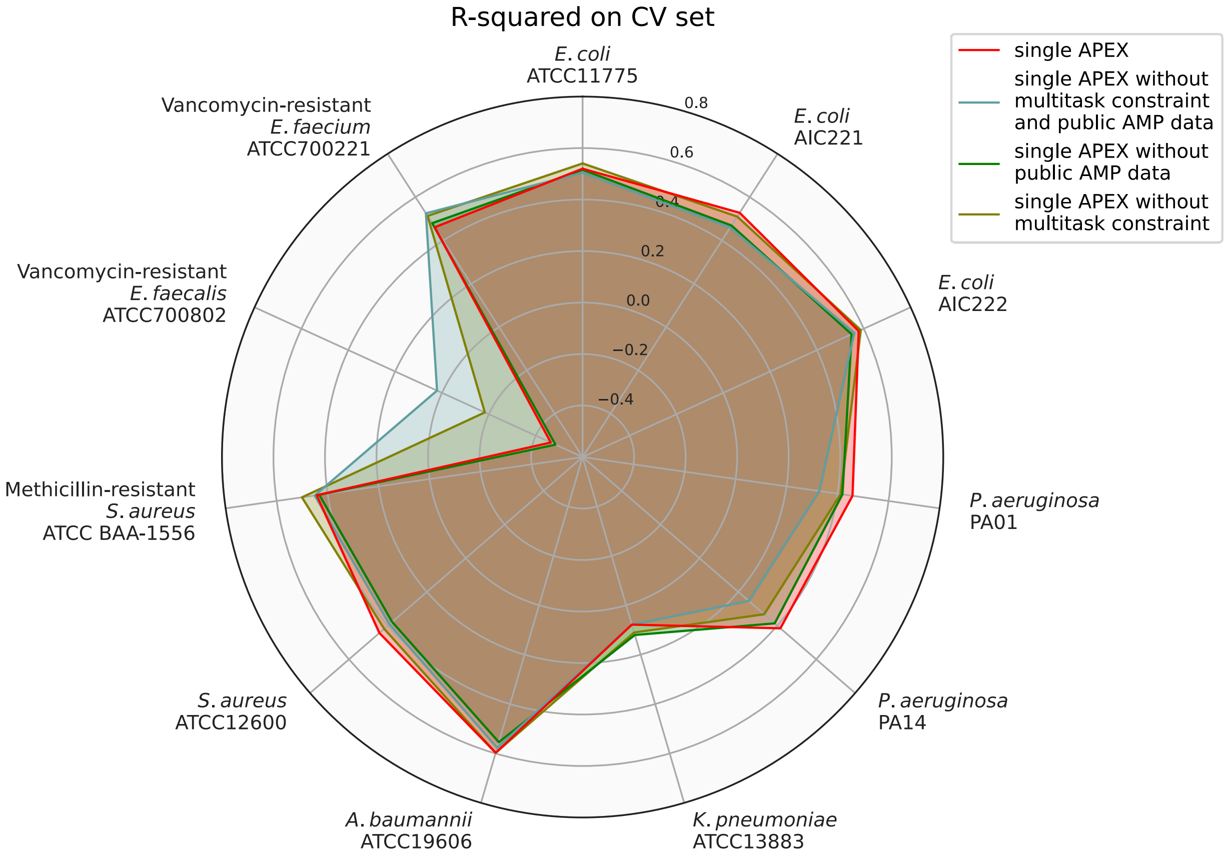

Supplementary Fig. 11 | Ablation study of the multitask learning strategy of APEX in terms of R-squared on the CV set. We divided our in-house peptide dataset into a CV set for hyperparameter tuning and ML model selection, and an independent dataset to evaluate prediction performance. The figure shows the average species-specific prediction performance of 5-fold cross validation in terms of R-squared for various APEX variants, including APEX without multitask constraint, APEX without using public AMP data during training, APEX without multitask constraint and public AMP data during training, and the original APEX (i.e., single APEX). The radius reflects the R-squared value.

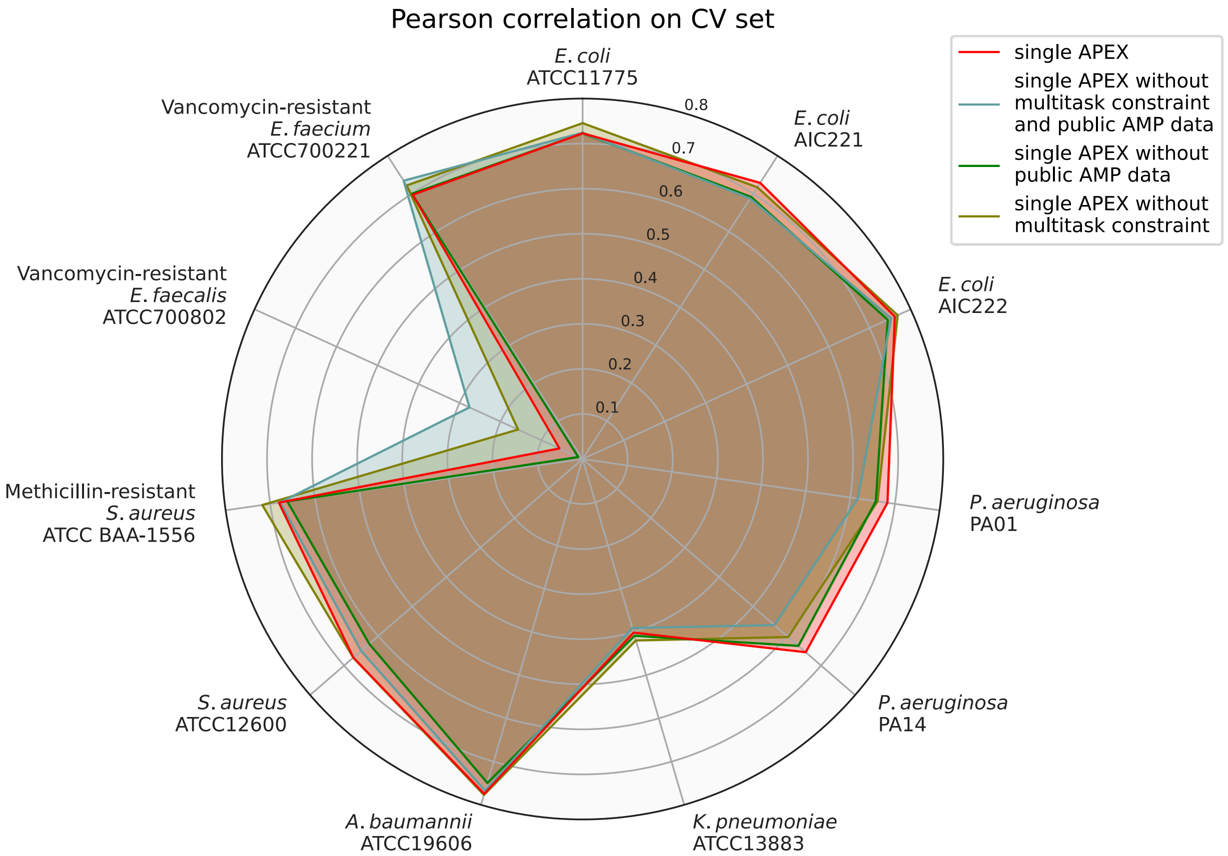

Supplementary Fig. 12 | Ablation study of the multitask learning strategy of APEX in terms of Pearson correlation on CV set. We divided our in-house peptide dataset into a CV set for hyperparameter tuning and ML model selection, and an independent dataset to evaluate prediction performance. The figure shows the average species-specific prediction performance of 5-fold cross validation in terms of Pearson correlation for various APEX variants, including APEX without multitask constraint, APEX without using public AMP data during training, APEX without multitask constraint and public AMP data during training, and the original APEX (i.e., single APEX). The radius reflects the Pearson correlation value.

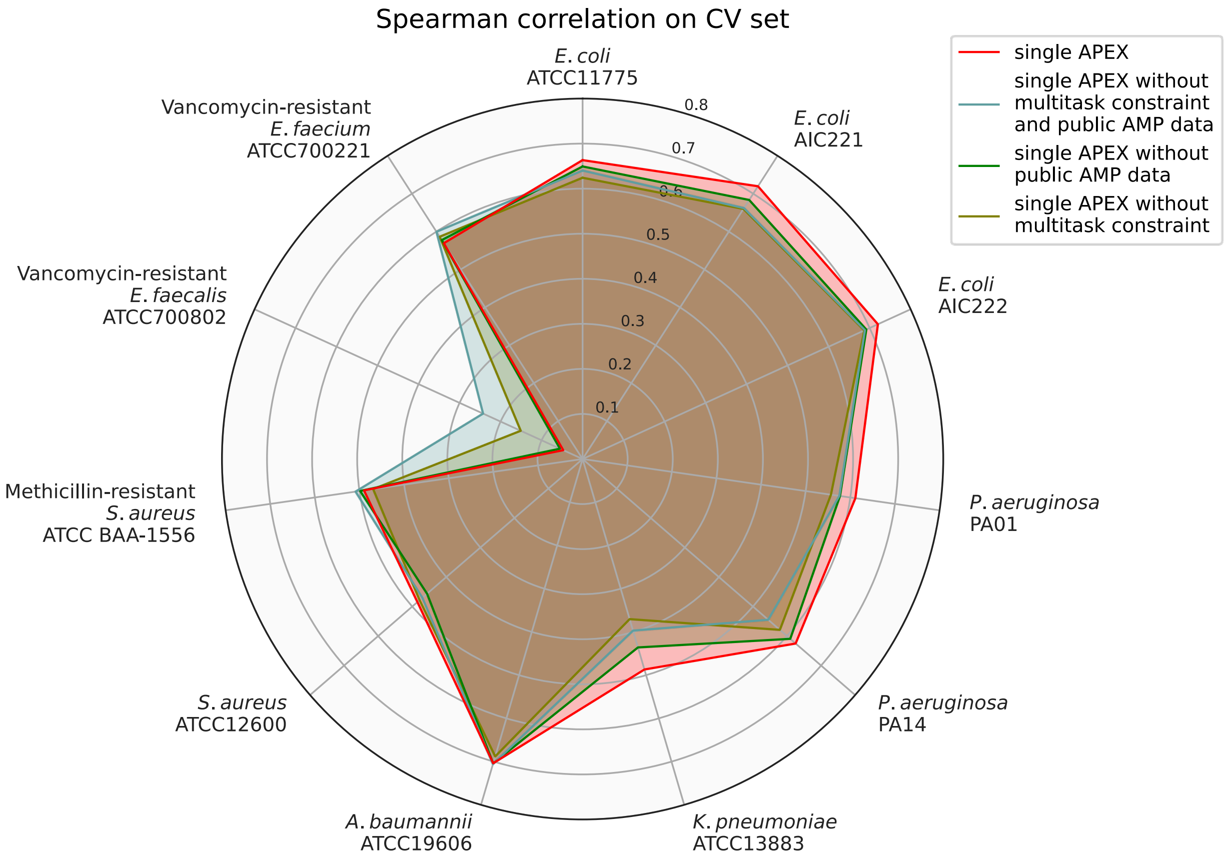

Supplementary Fig. 13 | Ablation study of the multitask learning strategy of APEX in terms of Spearman correlation on CV set. We divided our in-house peptide dataset into a CV set for hyperparameter tuning and ML model selection, and an independent dataset to evaluate prediction performance. The figure shows the average species-specific prediction performance of 5-fold cross validation in terms of Spearman correlation for various APEX variants, including APEX without multitask constraint, APEX without using public AMP data during training, APEX without multitask constraint and public AMP data during training, and the original APEX (i.e., single APEX). The radius reflects the Spearman correlation value.

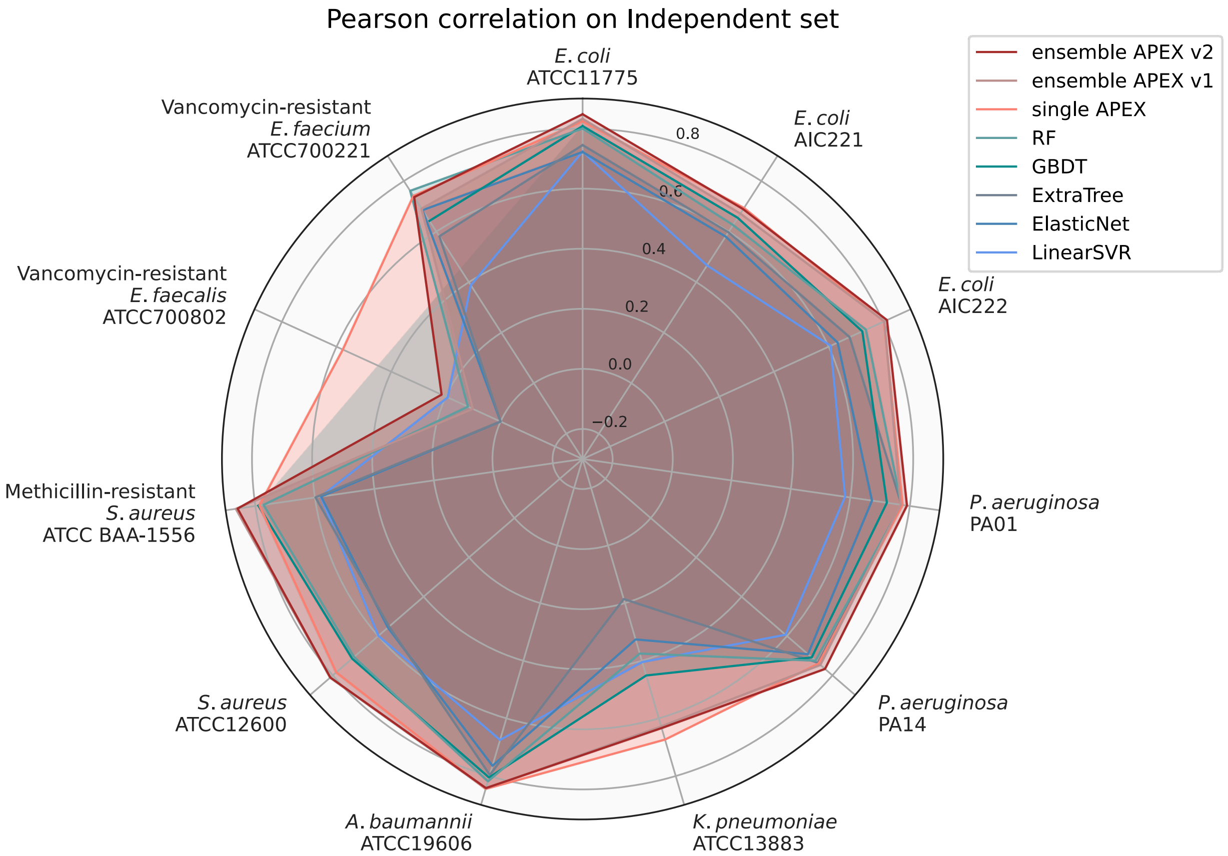

Supplementary Fig. 14 l Pearson correlation scores of various ML models on an independent set. We divided our in-house peptide dataset into a CV set for hyperparameter tuning and ML model selection, and an independent dataset to evaluate prediction performance. The figure shows the species-specific prediction performance in terms of Pearson correlation for various ML models that were trained on the CV set and evaluated on the independent set. The radius reflects the Pearson correlation value. RF: random forest; GBDT: gradient boosting decision tree; ExtraTree: extra-tree regressor; ElasticNet: elastic net; LinearSVR: linear support vector regression.

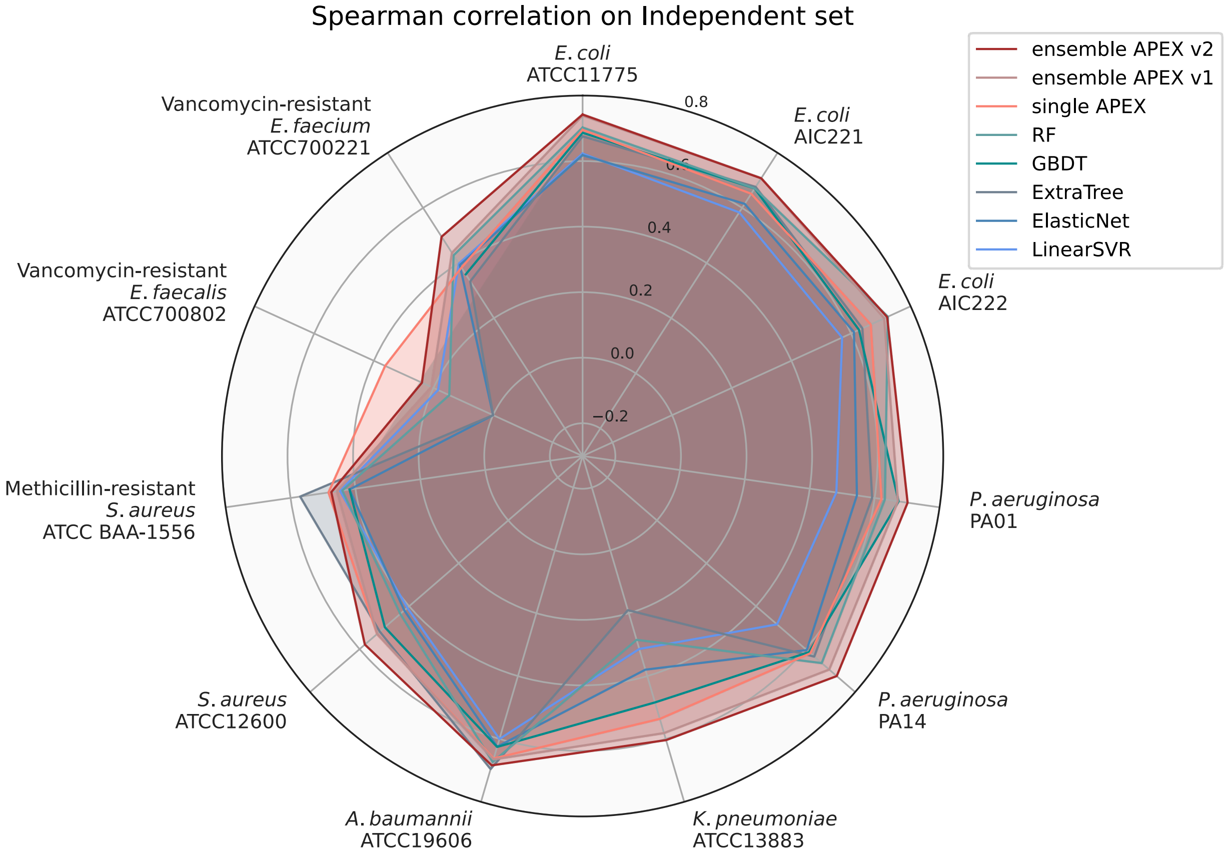

Supplementary Fig. 15 I Spearman correlation scores of various ML models on an independent set. We divided our in-house peptide dataset into a CV set for hyperparameter tuning and ML model selection, and an independent dataset to evaluate prediction performance. The figure shows the species-specific prediction performance in terms of Spearman correlation for various ML models that were trained on the CV set and evaluated on the independent set. The radius reflects the Spearman correlation value. RF: random forest; GBDT: gradient boosting decision tree; ExtraTree: extra-tree regressor; ElasticNet: elastic net; LinearSVR: linear support vector regression.

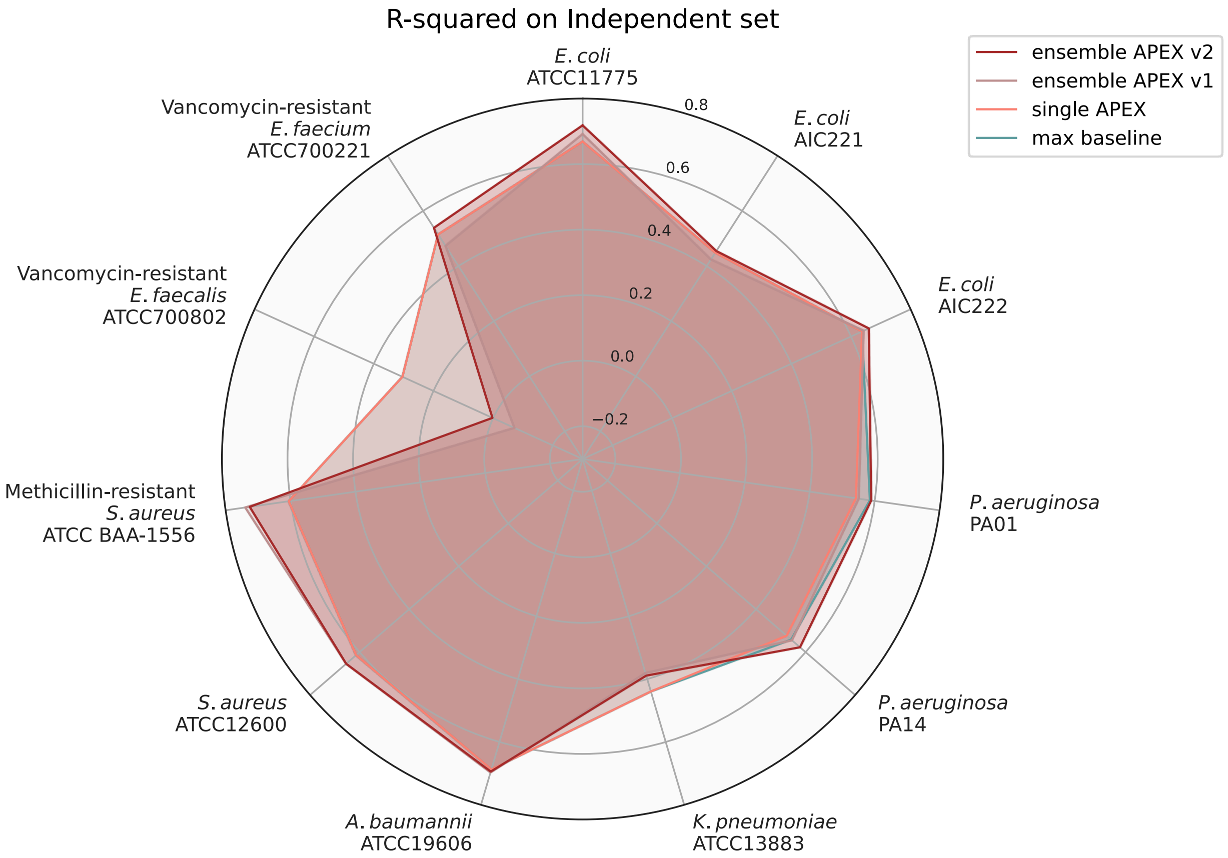

Supplementary Fig. 16 | R-squared scores of various ML models on an independent set. We divided our in-house peptide dataset into a CV set for hyperparameter tuning and ML model selection, and an independent dataset to evaluate prediction performance. The figure shows the species-specific prediction performance in terms of R-squared for APEX models and maximum performance from baseline ML models that were trained on the CV set and evaluated on the independent set. The radius reflects the R-squared value. Max baseline denotes the highest R-squared values from baseline ML models, including elastic net, linear support vector regression, extra-trees regressor, random forest and gradient boosting decision tree.

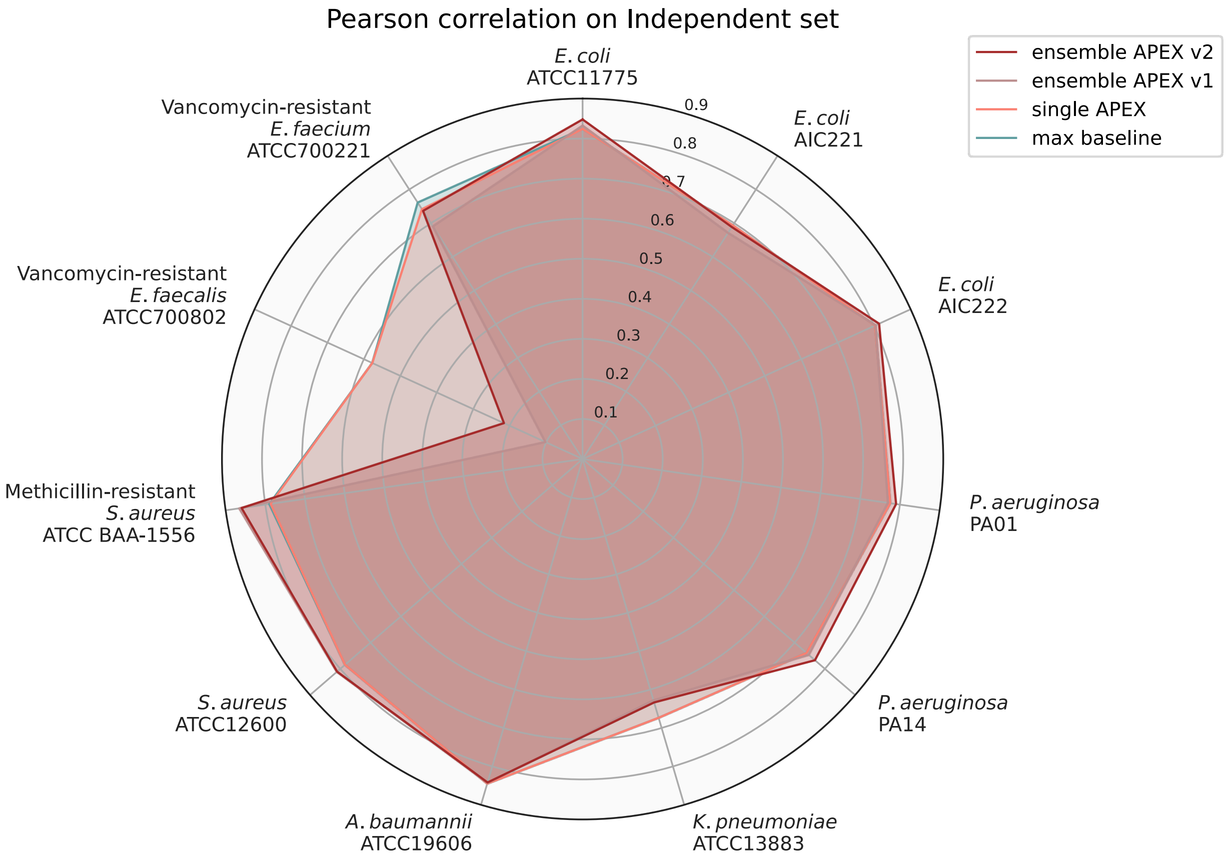

Supplementary Fig. 17 | Pearson correlation scores of various ML models on an independent set. We divided our in-house peptide dataset into a CV set for hyperparameter tuning and ML model selection, and an independent dataset to evaluate prediction performance. The figure shows the species-specific prediction performance in terms of Pearson correlation for APEX models and maximum performance from baseline ML models that were trained on the CV set and evaluated on the independent set. The radius reflects the Pearson correlation value. Max baseline denotes the highest Pearson correlation values from baseline ML models, including elastic net, linear support vector regression, extra-trees regressor, random forest and gradient boosting decision tree.

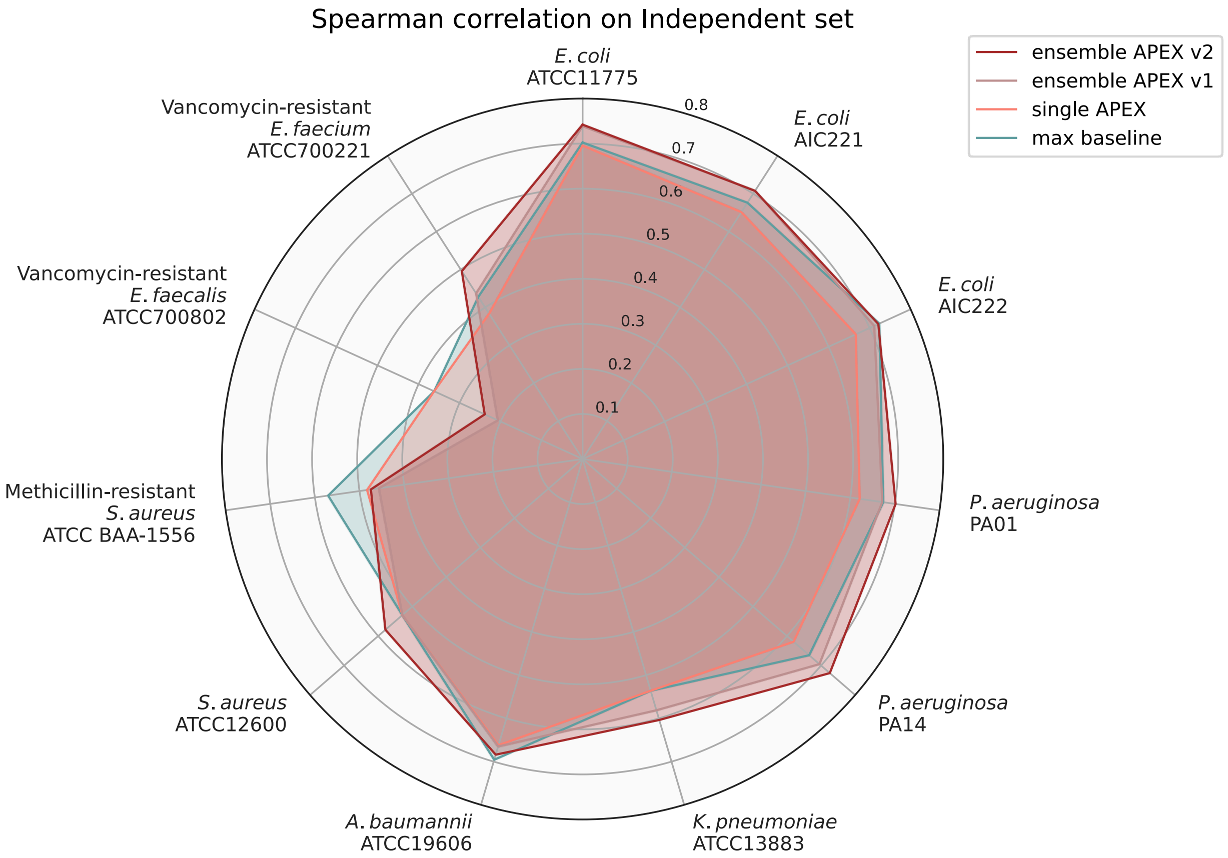

Supplementary Fig. 18 | Spearman correlation scores of various ML models on an independent set. We divided our in-house peptide dataset into a CV set for hyperparameter tuning and ML model selection, and an independent dataset to evaluate prediction performance. The figure shows the species-specific prediction performance in terms of Spearman correlation for APEX models and maximum performance from baseline ML models that were trained on the CV set and evaluated on the independent set. The radius reflects the Spearman correlation value. Max baseline denotes the highest Spearman correlation values from baseline ML models, including elastic net, linear support vector regression, extra-trees regressor, random forest and gradient boosting decision tree.

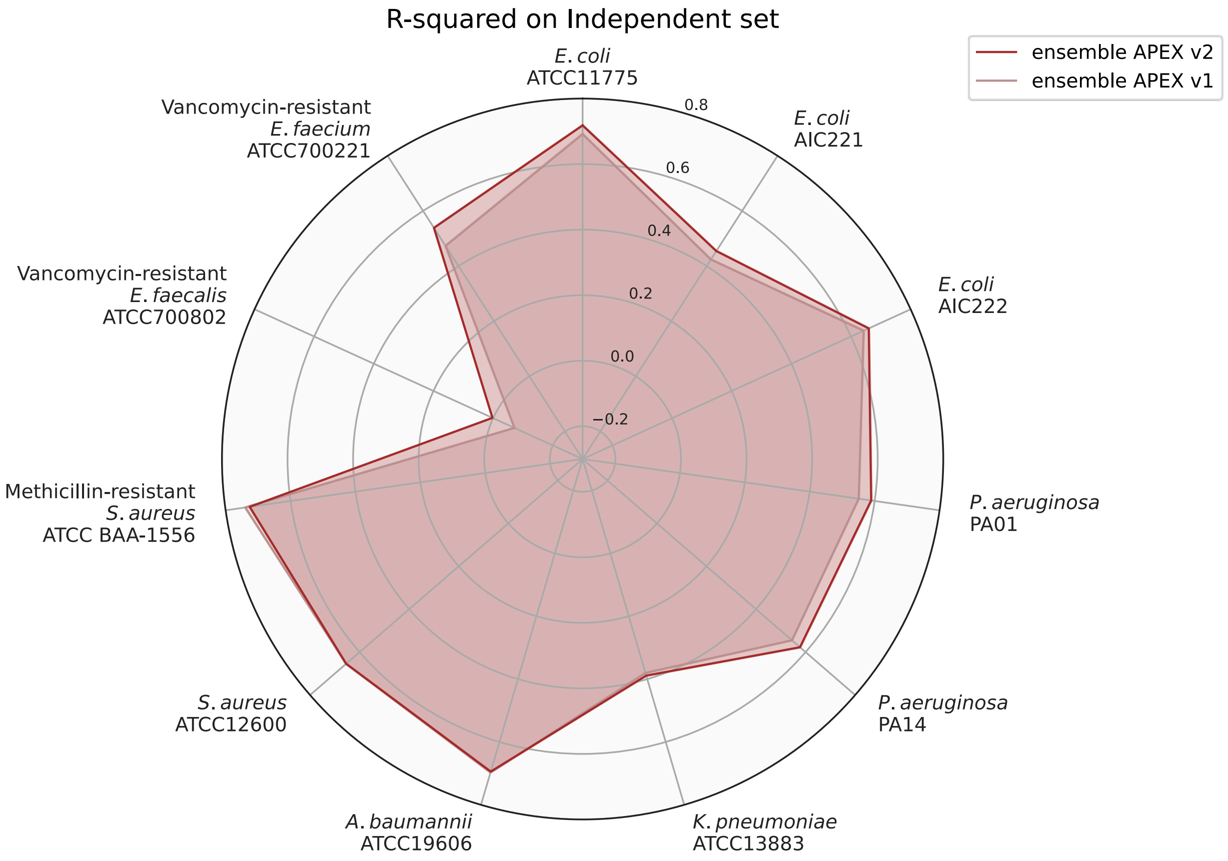

Supplementary Fig. 19 | R-squared scores of ensemble APEX v2 and v1 on an independent set. We divided our in-house peptide dataset into a CV set for hyperparameter tuning and ML model selection, and an independent dataset to evaluate prediction performance. The figure shows the species-specific prediction performance in terms of R-squared for two APEX variants that were trained on the CV set and evaluated on the independent set. The ensemble APEX v1 averaged the predictions from 8 different neural network architectures and training strategies. On top of ensemble APEX v1, ensemble APEX v2 further trained 5 copies under different random seeds for each base learner from ensemble APEX v1 to create 8×5 = 40 deep neural network predictors. The predictions from the 40 models were averaged to create the final prediction for ensemble APEX v2. The radius reflects the R-squared value.

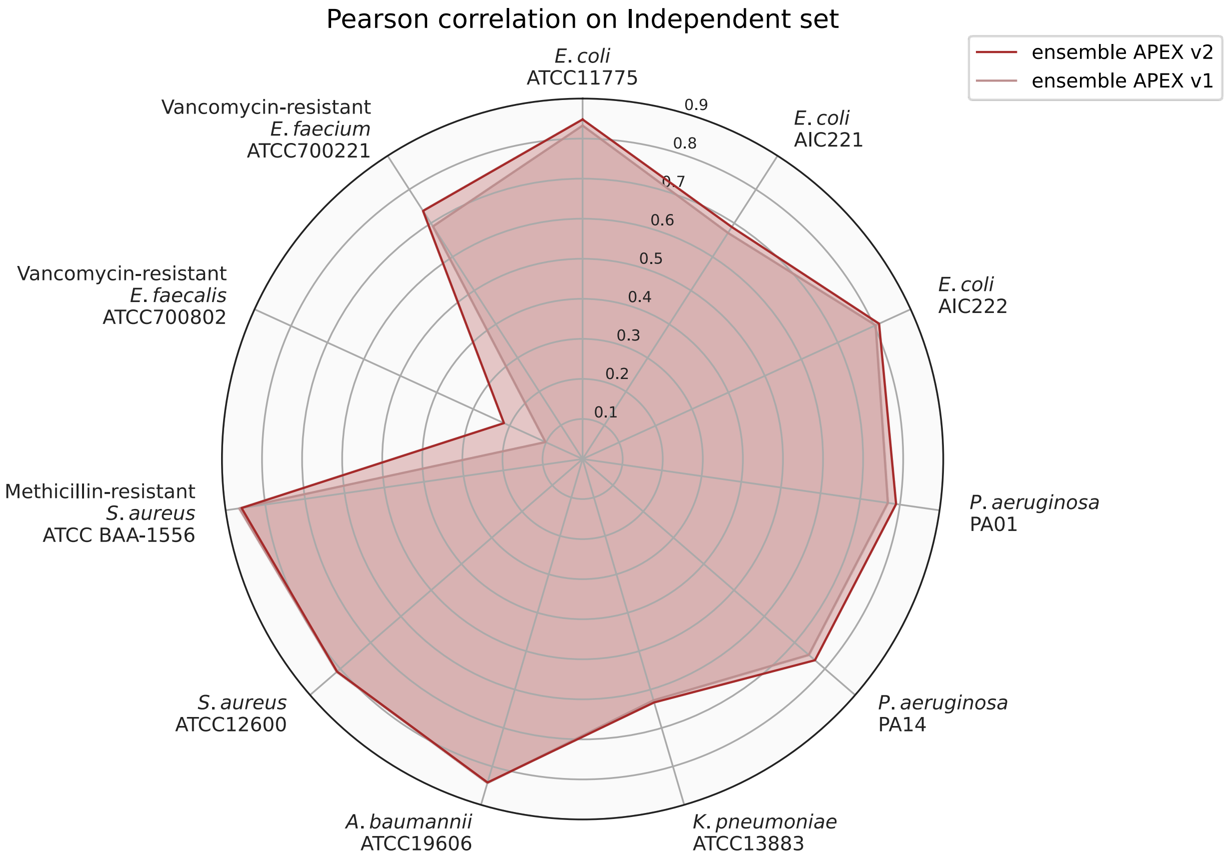

Supplementary Fig. 20 | Pearson correlation scores of ensemble APEX v2 and v1 on an independent set. We divided our in-house peptide dataset into a CV set for hyperparameter tuning and ML model selection, and an independent dataset to evaluate prediction performance. The figure shows the species-specific prediction performance in terms of Pearson correlation for two APEX variants that were trained on the CV set and evaluated on the independent set. The ensemble APEX v1 averaged the predictions from 8 different neural network architectures and training strategies. On top of ensemble APEX v1, ensemble APEX v2 further trained 5 copies under different random seeds for each base learner from ensemble APEX v1 to create 8×5 = 40 deep neural network predictors. The predictions from the 40 models were averaged to create the final prediction for ensemble APEX v2. The radius reflects the Pearson correlation value.

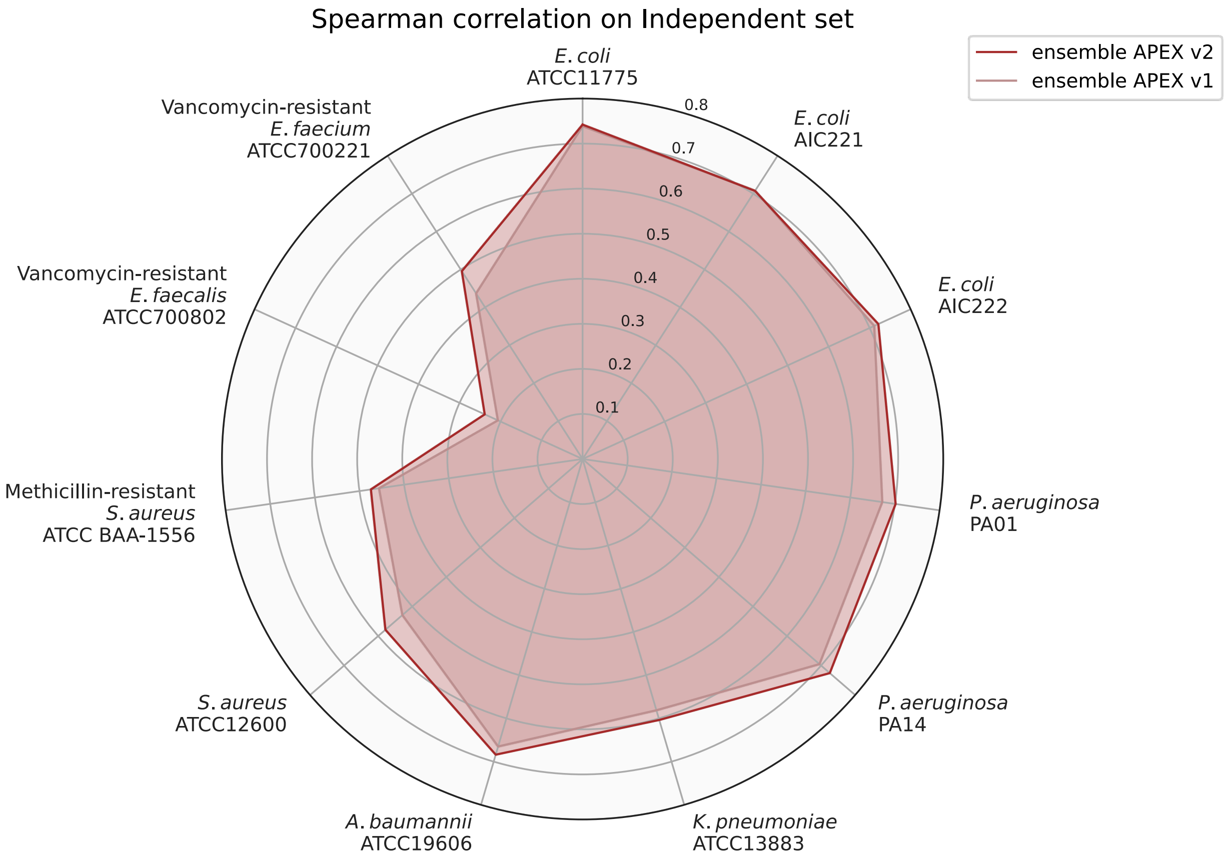

Supplementary Fig. 21 | Spearman correlation scores of ensemble APEX v2 and v1 on an independent set. We divided our in-house peptide dataset into a CV set for hyperparameter tuning and ML model selection, and an independent dataset to evaluate prediction performance. The figure shows the species-specific prediction performance in terms of Spearman correlation for two APEX variants that were trained on the CV set and evaluated on the independent set. The ensemble APEX v1 averaged the predictions from 8 different neural network architectures and training strategies. On top of ensemble APEX v1, ensemble APEX v2 further trained 5 copies under different random seeds for each base learner from ensemble APEX v1 to create 8×5 = 40 deep neural network predictors. The predictions from the 40 models were averaged to create the final prediction for ensemble APEX v2. The radius reflects the Spearman correlation value.

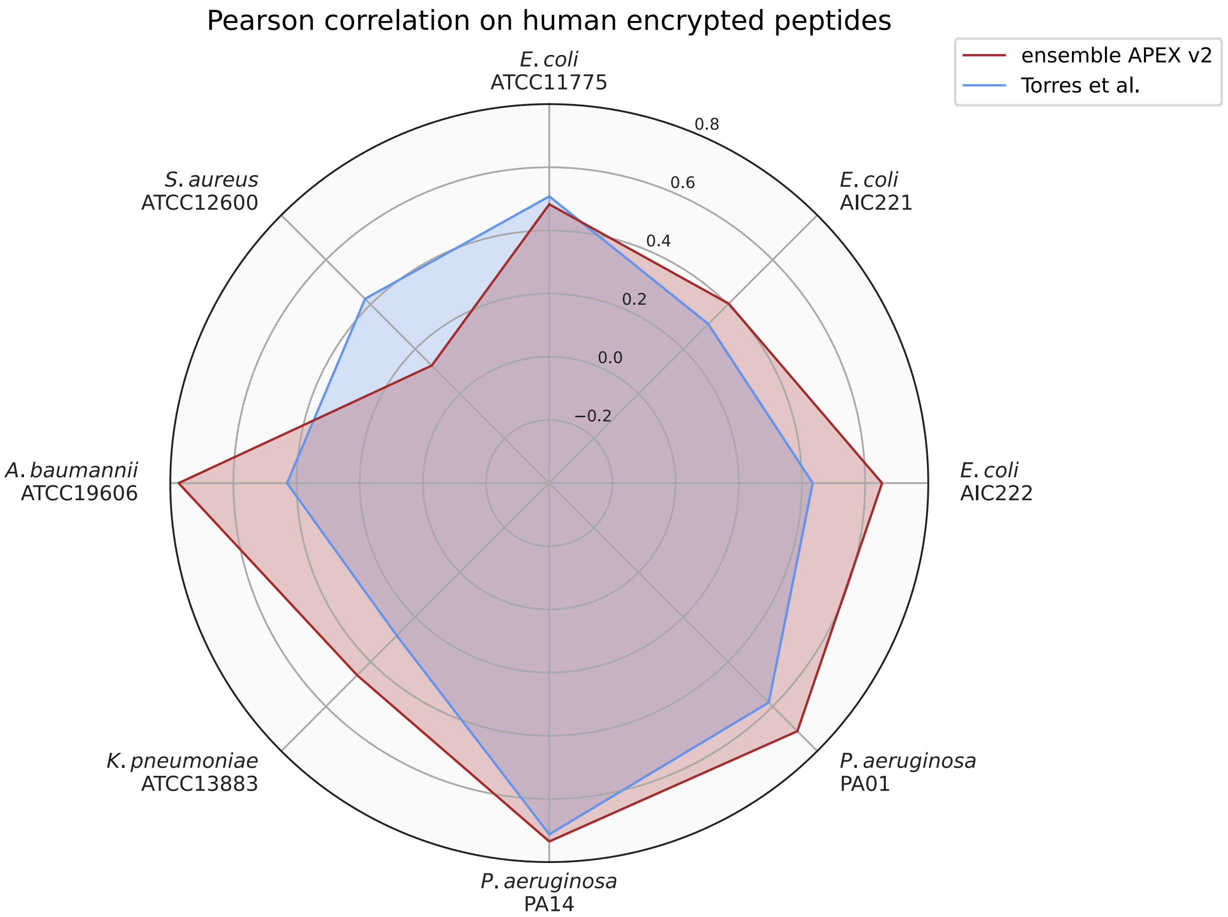

Supplementary Fig. 22 | Pearson correlation scores of ensemble APEX v2 and the scoring function used to identify modern human encrypted peptides. The human encrypted peptides from Torres et al.[1] constitute a subset of our in-house peptide dataset. We treated this subset as the test data and excluded it from ML model training. The figure shows the species-specific prediction performance in terms of Pearson correlation for ensemble APEX v2 and the AMP scoring function used by Torres et al.[1]. The radius reflects the Pearson correlation value.

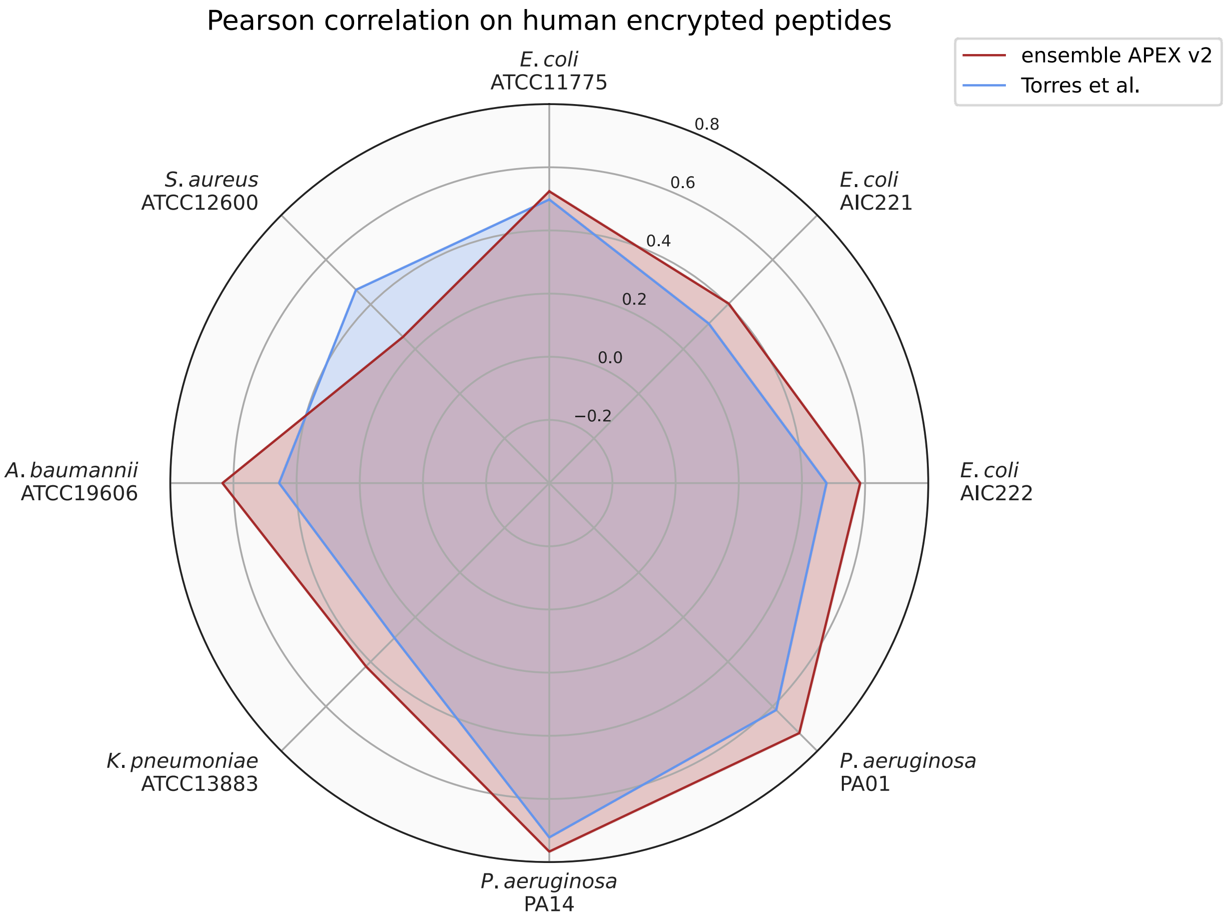

Supplementary Fig. 23 | Spearman correlation scores of ensemble APEX v2 and the scoring function used to identify modern human encrypted peptides. The human encrypted peptides from Torres et al.[1] constitute a subset of our in-house peptide dataset. We treated this subset as the test data and excluded it from ML model training. The figure shows the species-specific prediction performance in terms of Spearman correlation for ensemble APEX v2 and the AMP scoring function used in Torres et al.[1]. The radius reflects the Spearman correlation value.

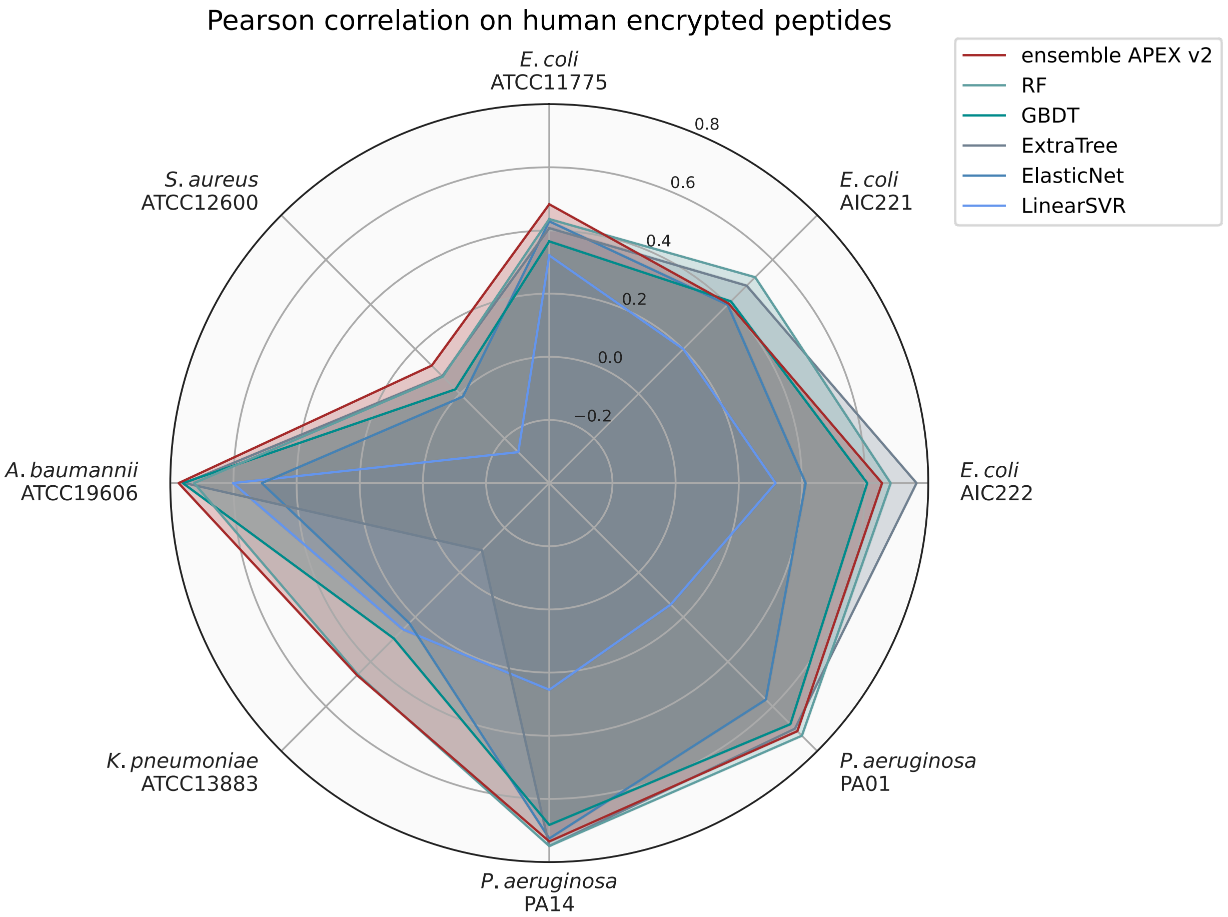

Supplementary Fig. 24 | Pearson correlation scores of various ML models used to identify modern human encrypted peptides. The human encrypted peptides from Torres et al.[1] constitute a subset of our in-house peptide dataset. We treated this subset as the test data and excluded it from ML model training. The figure shows the species-specific prediction performance in terms of Pearson correlation for various ML models. The radius reflects the Pearson correlation value. RF: random forest; GBDT: gradient boosting decision tree; ExtraTree: extra-tree regressor; ElasticNet: elastic net; LinearSVR: linear support vector regression.

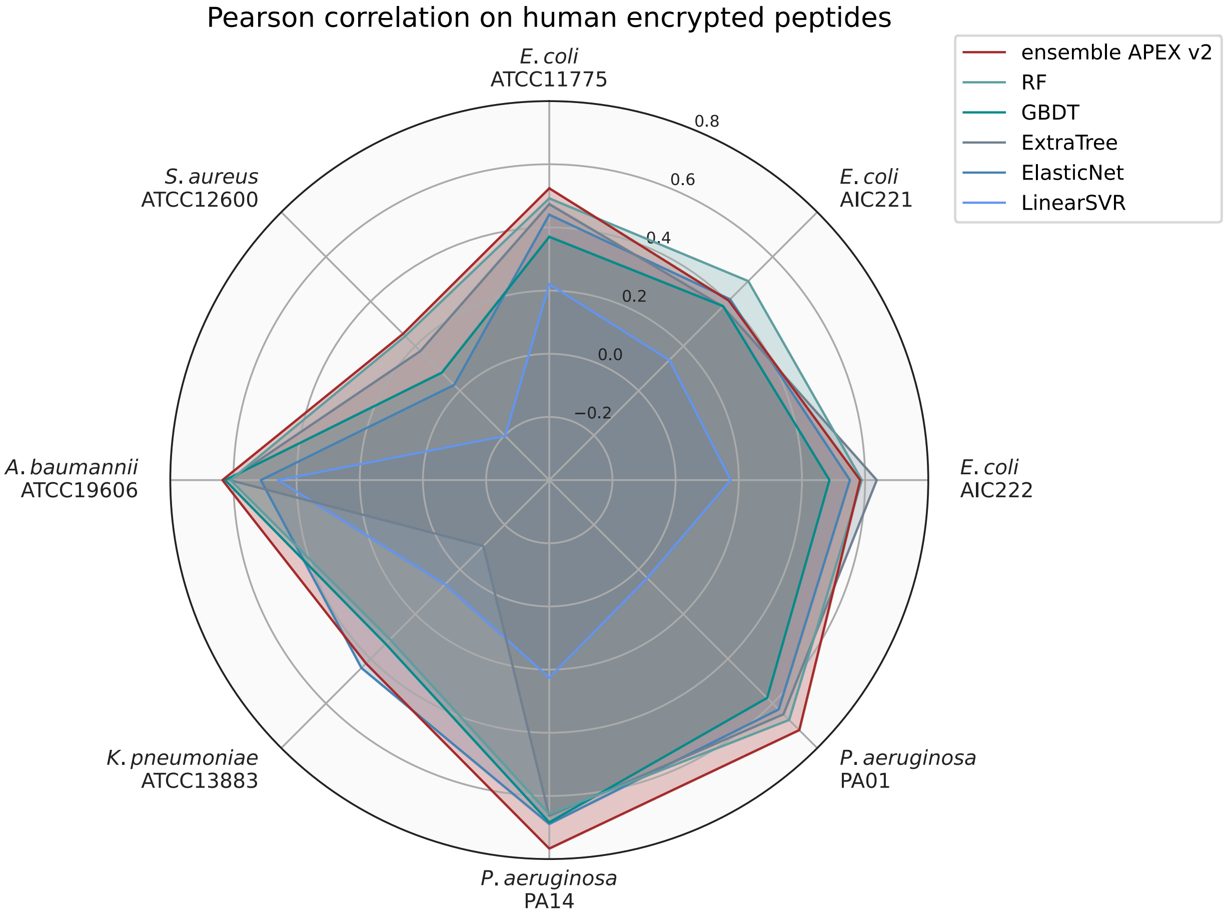

Supplementary Fig. 25 | Spearman correlation scores of various ML models used to identify modern human encrypted peptides. The human encrypted peptides from Torres et al.[1] constitute a subset of our in-house peptide dataset. We treated this subset as the test data and excluded it from ML model training. The figure shows the species-specific prediction performance in terms of Spearman correlation for various ML models. The radius reflects the Spearman correlation value. RF: random forest; GBDT: gradient boosting decision tree; ExtraTree: extra-tree regressor; ElasticNet: elastic net; LinearSVR: linear support vector regression.

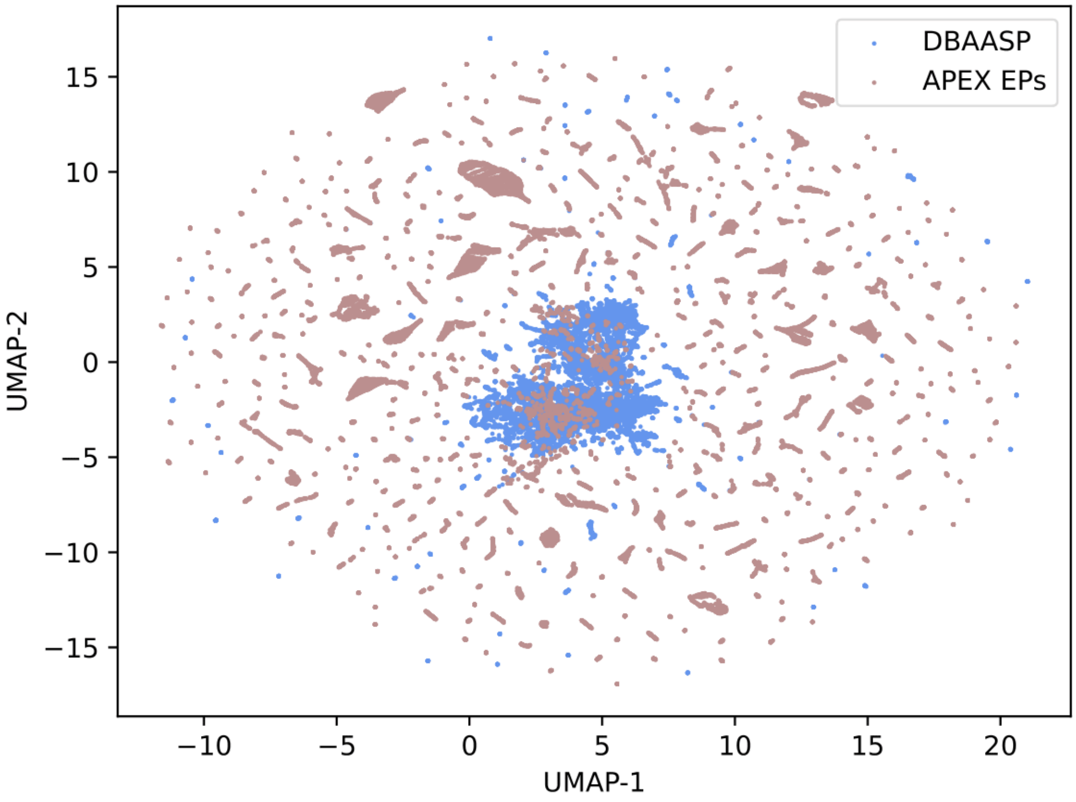

Supplementary Fig. 26 | Sequence space exploration using a similarity matrix. The graph represents a bidimensional sequence space visualization of peptide sequences found in DBAASP and antimicrobial EPs discovered by APEX**.** We used sequence alignment to generate a sequence similarity matrix for all peptide sequences in DBAASP and the 37,176 antimicrobial EPs predicted by APEX. Each row in the similarity matrix corresponds to a feature representation of a peptide in terms of amino acid residues. Uniform Manifold Approximation and Projection (UMAP) was used to reduce the feature representation to two dimensions for visualization purposes.

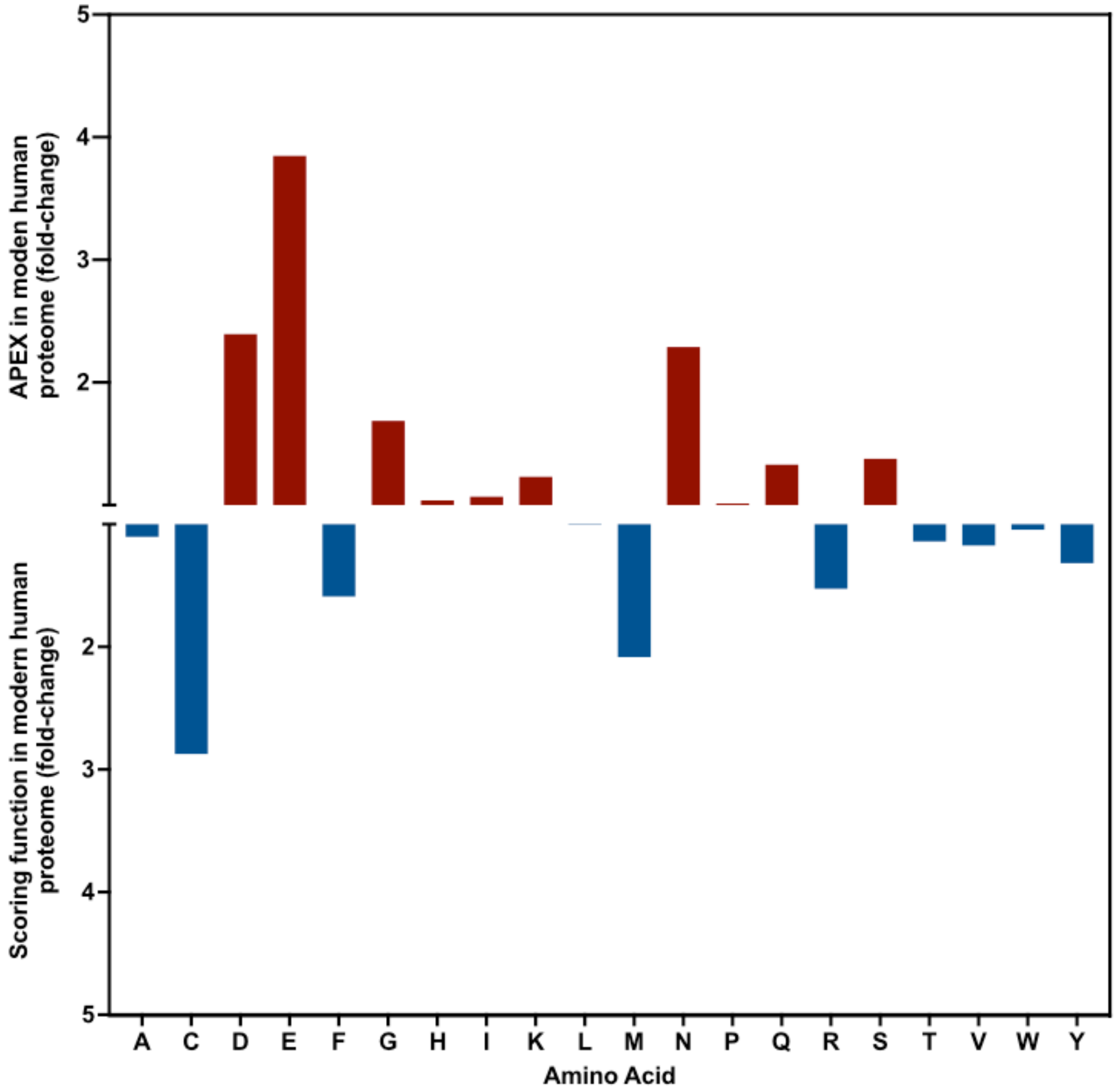

Supplementary Fig. 27 | Relative abundance of the amino acid content of encrypted peptides (EPs) from the modern human proteome identified by APEX (top) and the scoring function (bottom). The frequency of amino acid was normalized by the total number of amino acid residue counts. The ratio between normalized amino acid frequencies highlights the overrepresentation of negatively charged residues (D and E), glycine (G), and uncharged polar residues (N, Q, and S) in peptides identified by APEX. The scoring function preferably identified encrypted peptides with a high frequency of cysteine (C), methionine (M), arginine (R) and phenylalanine (F) residues.

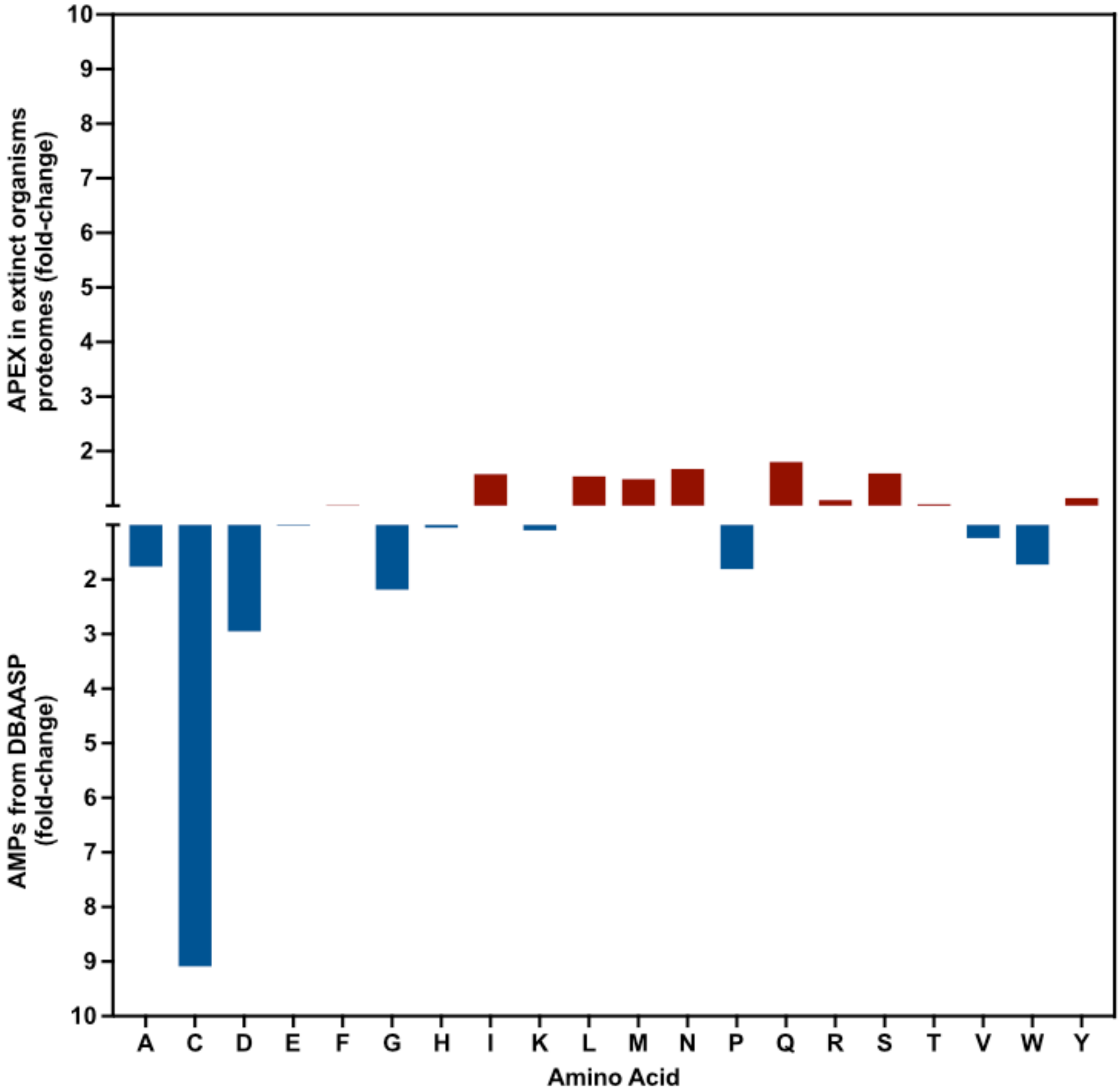

Supplementary Fig. 28 | Relative abundance of the amino acid content of encrypted peptides (EPs) identified by APEX from the proteomes of extinct organisms (top) compared to known AMPs from DBAASP (bottom). The frequency of each amino acid residue was normalized by the total number of amino acid residue counts. The ratio between AEPs identified by APEX and known peptide from DBAASP highlights the relative abundance of aliphatic (L and I) and uncharged polar (M, N, Q, and S) residues in sequences from extinct organisms, whereas peptides from DBAASP present a higher content of negatively charged (D) and aromatic (W) residues, as well as residues known for their role in secondary structure (C, G, P).

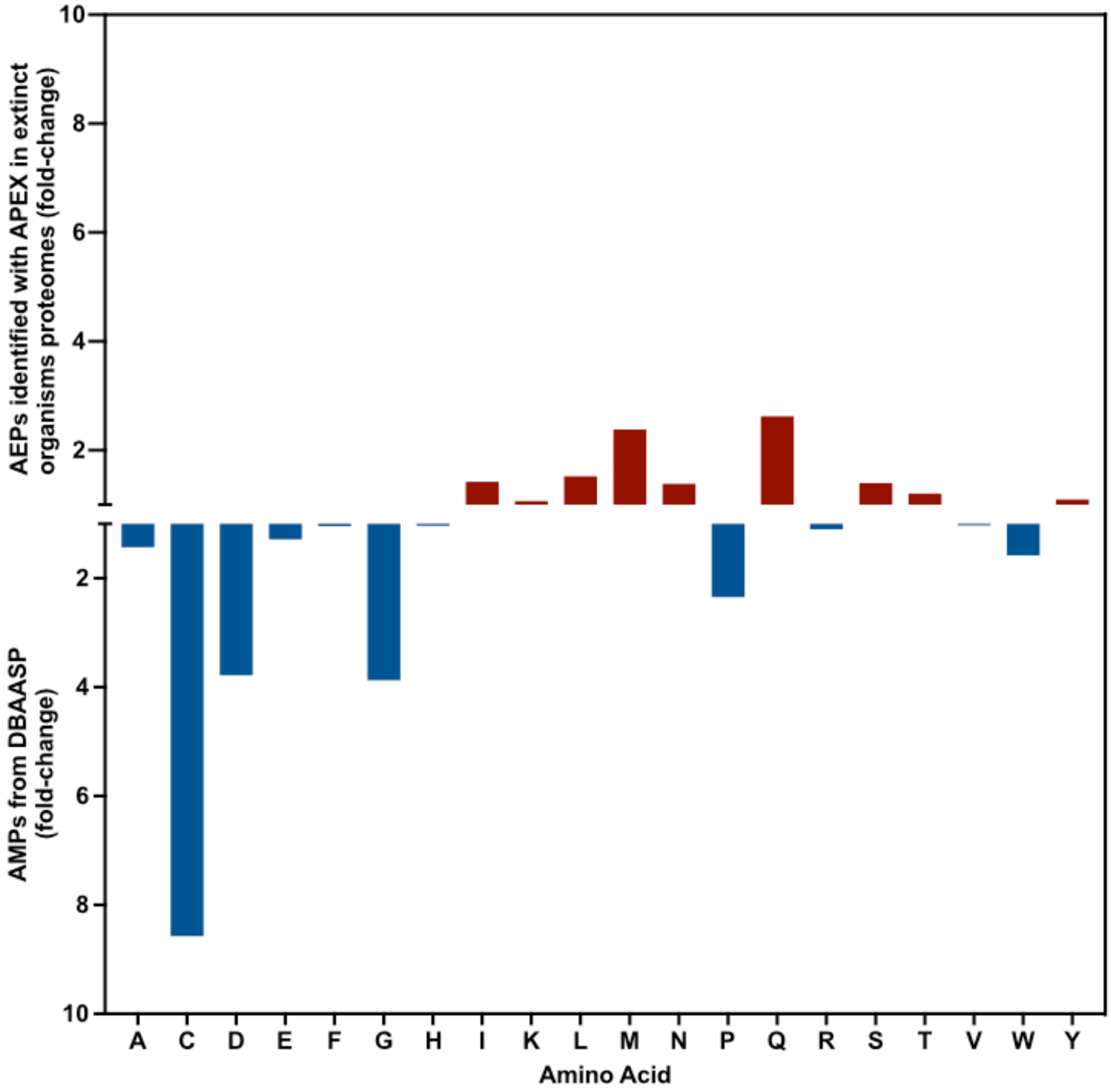

Supplementary Fig. 29 I Relative abundance of the amino acid content of archaic encrypted peptides (AEPs) identified by APEX from the proteomes of extinct organisms (top) compared to known AMPs from DBAASP (bottom). The frequency of each amino acid was normalized by the total number of amino acid residue counts. The ratio between encrypted peptides from extinct proteins identified by APEX and known peptides from the DBAASP highlights the relative abundance of aliphatic (L and I) and uncharged polar (M, N, Q, and S) residues in sequences from extinct proteins, whereas peptides from the DBAASP had a higher content of negatively charged (D and E) and aromatic (W) residues, as well as residues known for their role in secondary structure (A, C, G, P).

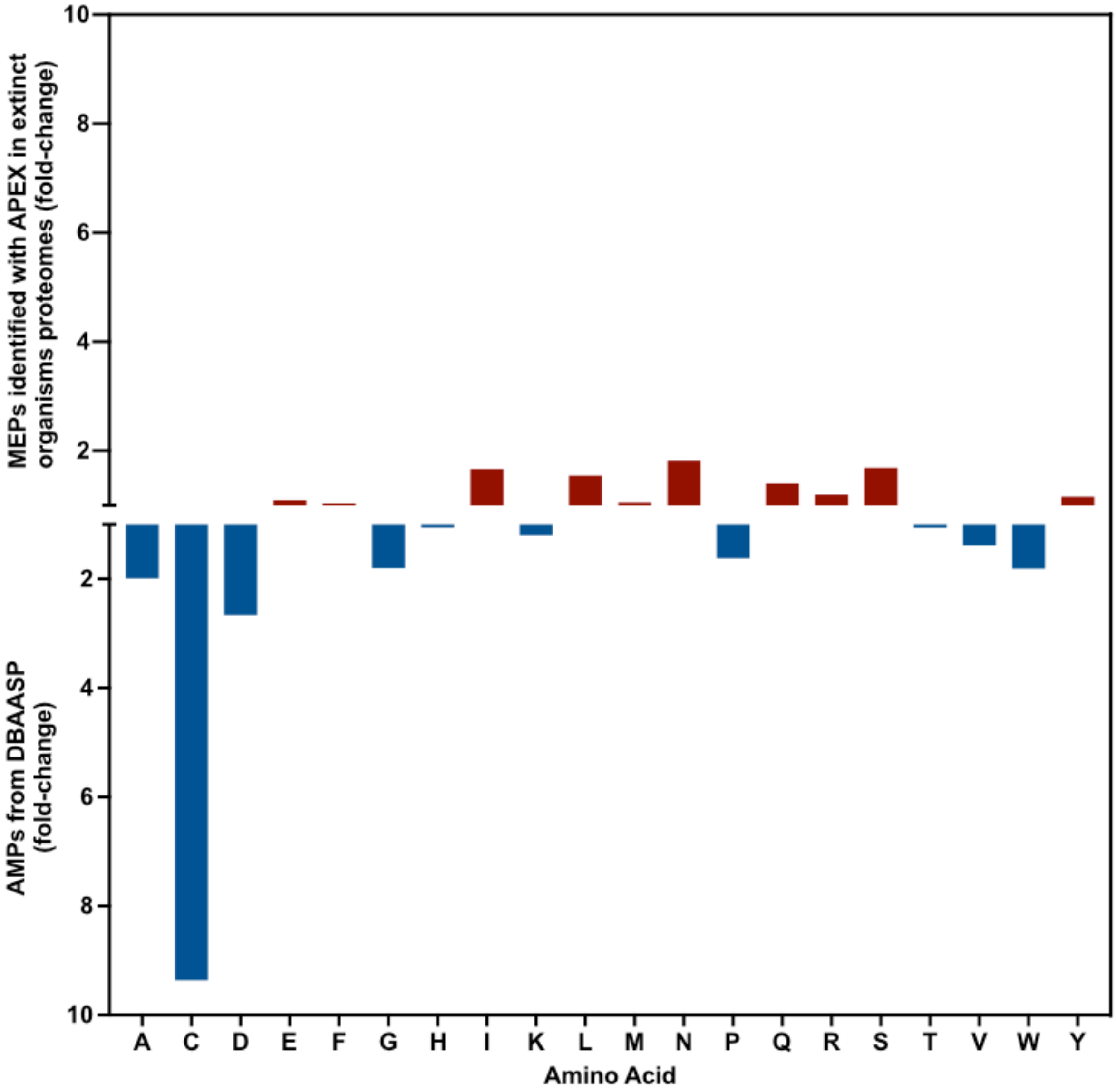

Supplementary Fig. 30 I Relative abundance of the amino acid content of MEPs identified by APEX from the proteomes of extinct organisms compared to known AMPs from DBAASP. The frequency of amino acid was normalized by the total number of amino acid residue counts. The ratio between encrypted peptides from extinct organisms that still exist in modern organisms proteomes identified by APEX and known peptide from DBAASP highlights the relative abundance of aliphatic (L and I) and polar non-charged (M, N, Q, and S) residues in sequences from extinct organisms proteins, whereas peptides from DBAASP present a higher content of negatively charged (D), aromatic (W), and residues known for their role on secondary structure (A, C, G, P).

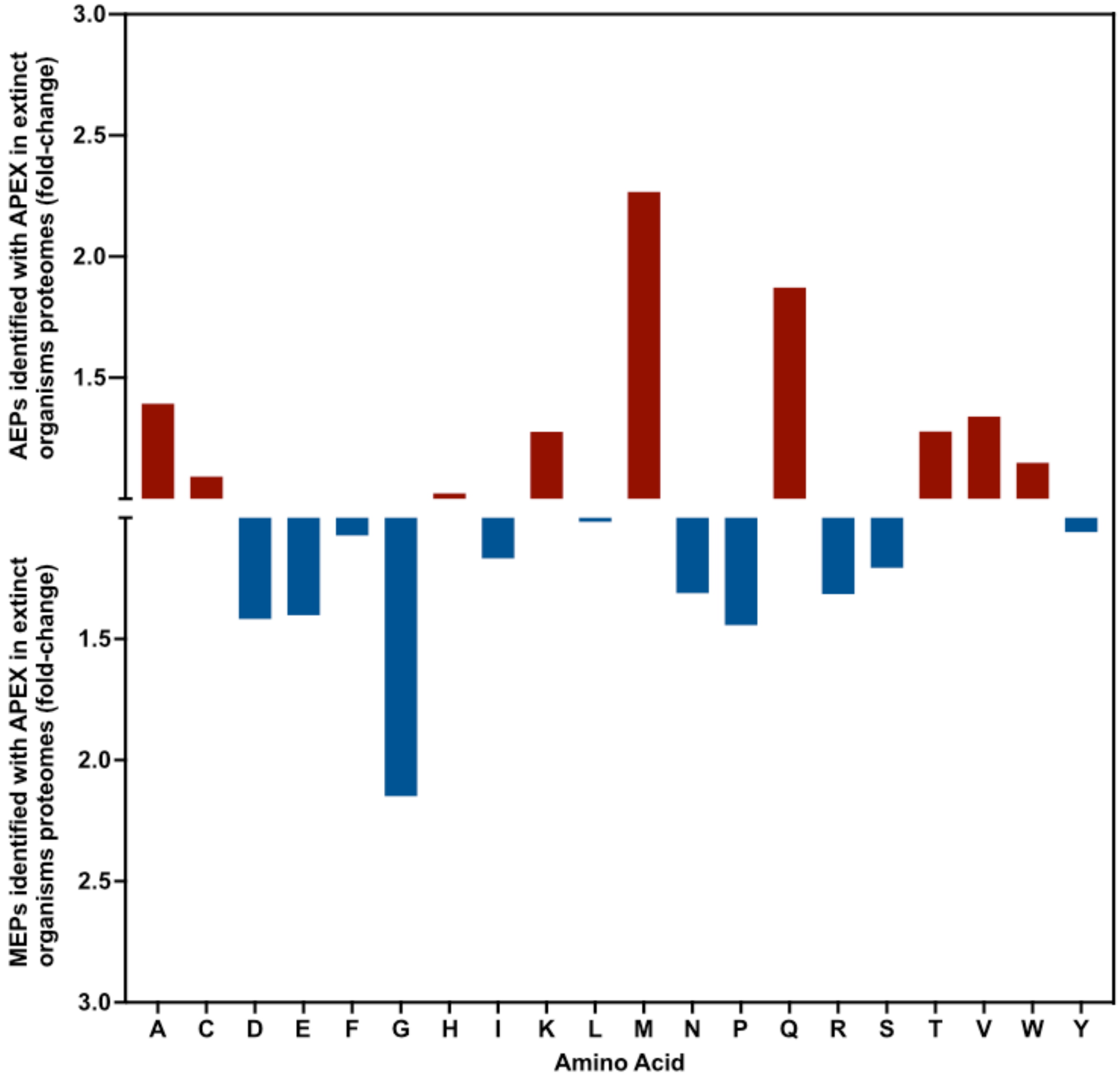

Supplementary Fig. 31 | Relative abundance of the amino acid content of AEPs and MEPs identified by APEX from the proteomes of extinct organisms. The frequency of amino acid was normalized by the total number of amino acid residue counts. The ratio between encrypted peptides from extinct proteins that are not present in modern organisms and from proteins that still exist in modern organisms identified by APEX highlights the relative abundance of polar non-charged (M and Q) residues in sequences from extinct proteins, whereas encrypted peptides from still existing proteins present a higher content of glycine.

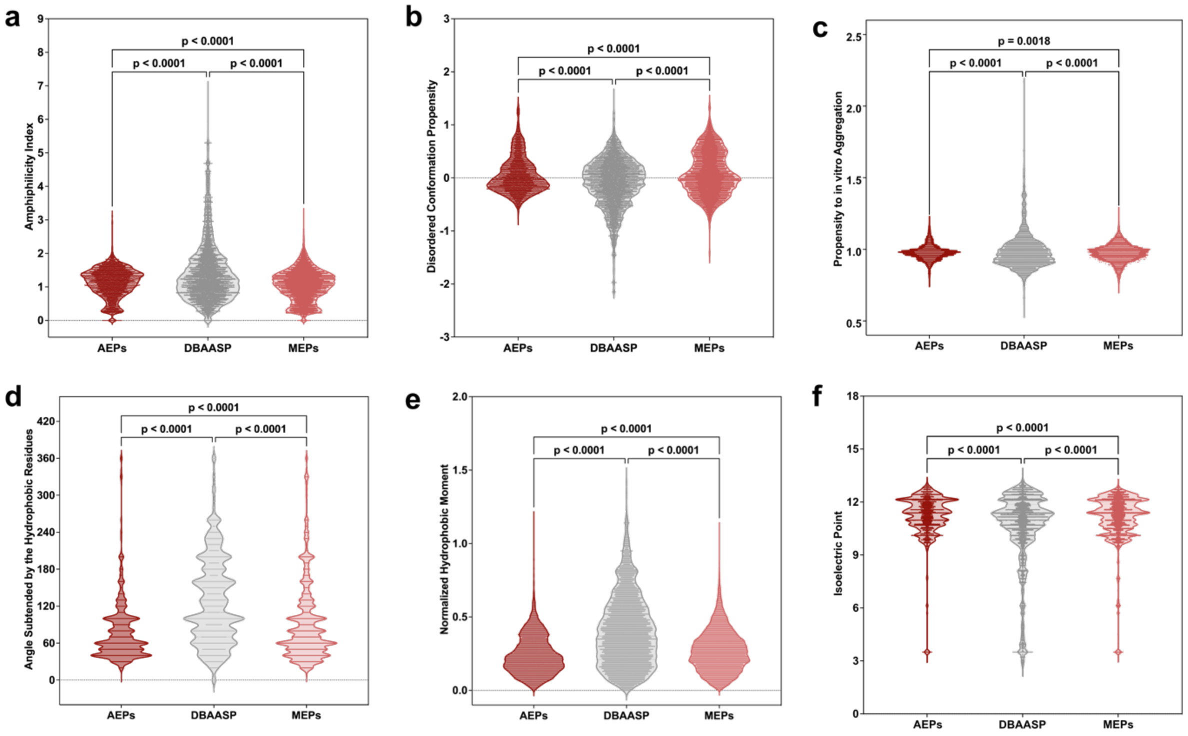

Supplementary Fig. 32 | Physicochemical features of AEPs and MEPs identified by APEX in extinct organisms compared to AMPs from DBAASP. (a) Amphiphilicity index and **(b)** disordered conformation propensity; both properties closely correlated with mechanism of action, *i.e.*, how the peptides interact with membrane lipids to exert antimicrobial activity. **(c)** Propensity to aggregate *in vitro* and **(d)** angle subtended by the hydrophobic residues; both properties are correlated with supramolecular arrangement of the molecules and toxicity. **(e)** Hydrophobic moment normalized by peptide length and **(f)** isoelectric point; both properties are also related to the amphipathicity of the molecules that influence directly on their interactions with bacterial membranes. Statistical significance was obtained using two-tailed t-tests followed by Mann-Whitney test; p values are shown in the graph. The solid line inside each box represents the mean value obtained for each group.

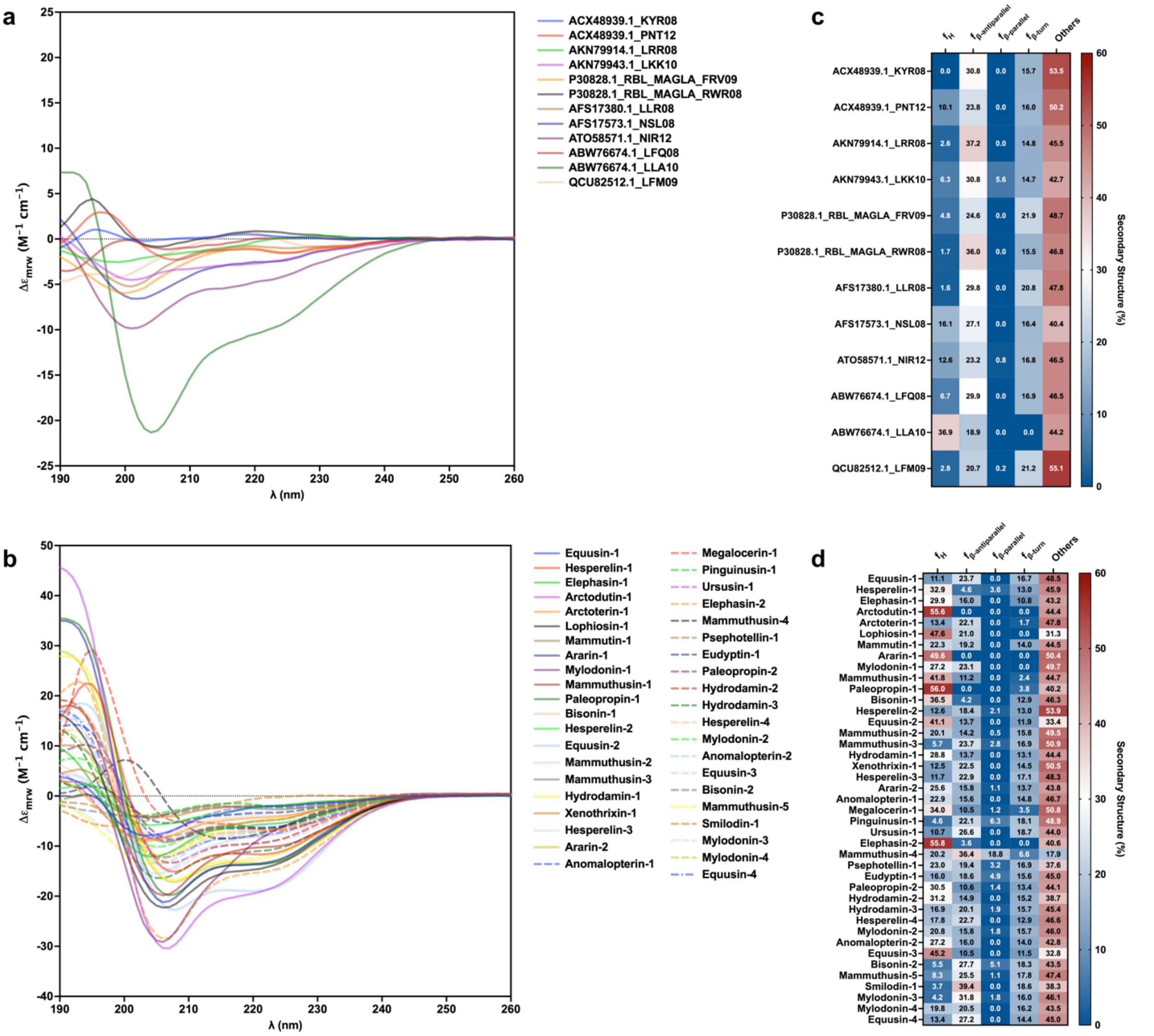

Supplementary Fig. 33 | Secondary structure of active EPs predicted by the scoring function and APEX in helical inducer medium. Circular dichroism experiments with encrypted peptides (EPs) from extinct organisms generated by **(a)** the scoring function based on length, hydrophobicity, and net charge, which predominantly identified unstructured peptides, and **(b)** the deep learning model, which identified EPs having a higher helical content than the EPs predicted by the scoring function. **(c-d)** Heat maps of the secondary structure basis components values obtained using the algorithm BeStSel[2] for EPs identified by the scoring function **(c)** or APEX **(d)**. Assays were performed in a J-1500 (Jasco circular dichroism spectrophotometer), and the circular dichroism spectra were recorded after three accumulations at 25 °C, using a 1 mm path length quartz cell, between 260 and 190 nm at 50 nm min^{-1}, with a bandwidth of 0.5 nm.

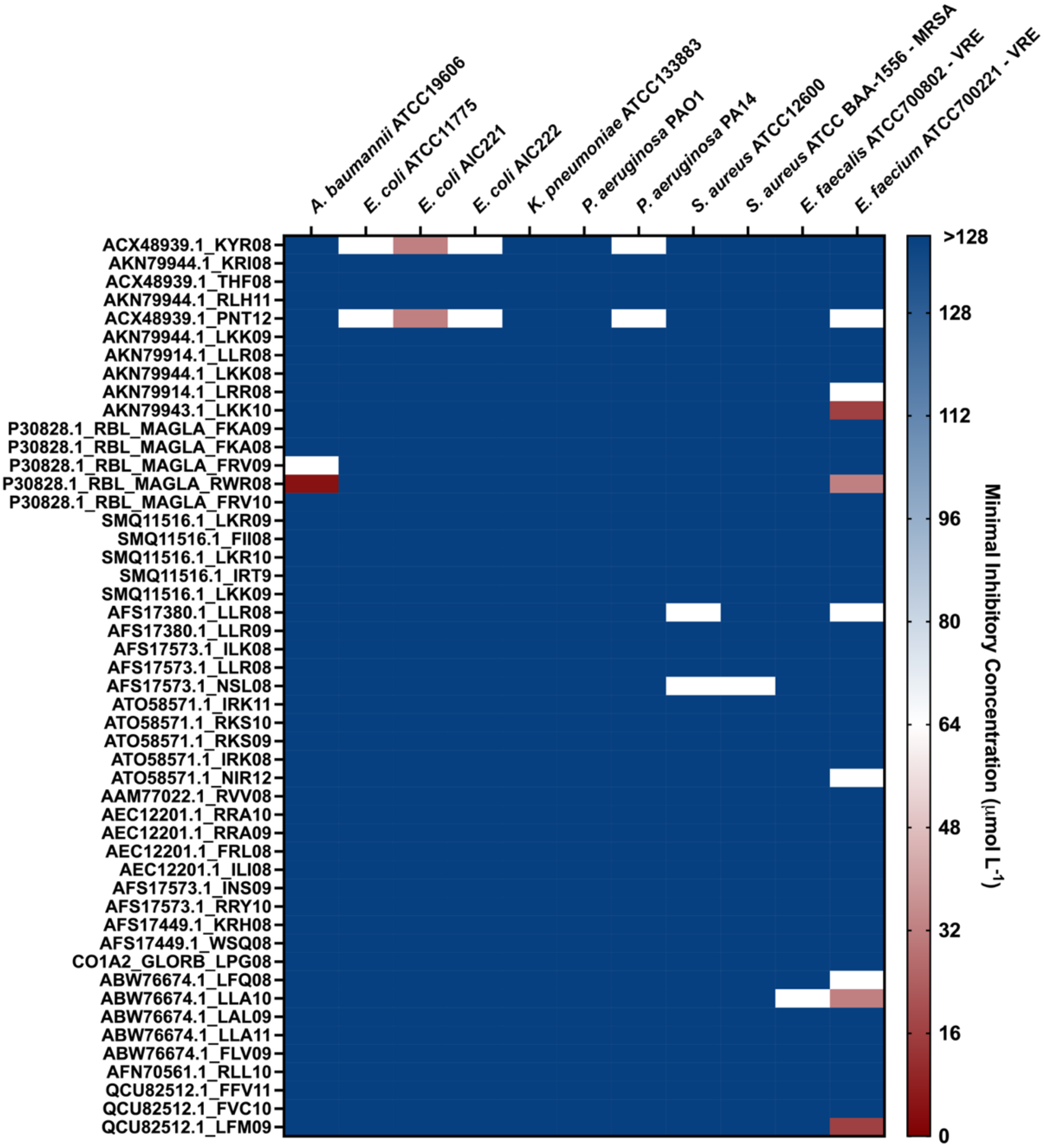

Supplementary Fig. 34 l Antimicrobial activity of encrypted peptides from extinct organisms predicted by the scoring function. Heat map of the antimicrobial activities (µmol L^{-1}) of the encrypted peptides (EPs) from extinct organisms predicted by the scoring function (Torres et al.[1]) based on length, hydrophobicity, and net charge, for 11 pathogens, including four strains resistant to conventional antibiotics. All the EPs identified by the scoring function were modern EPs (MEPs), *i.e.*, EPs identified in extinct organisms but also present in modern proteins. Briefly, 10^6 bacterial cells and serially diluted MEPs (0–128 µmol L^{-1}) were incubated at 37 °C. One day post-treatment, the optical density at 600 nm was measured in a microplate reader to determine whether the EPs from extinct organisms inhibited bacterial growth *in vitro*. Assays were performed in three independent replicates, and MIC values in the heat map are the arithmetic mean of the replicates in each condition.

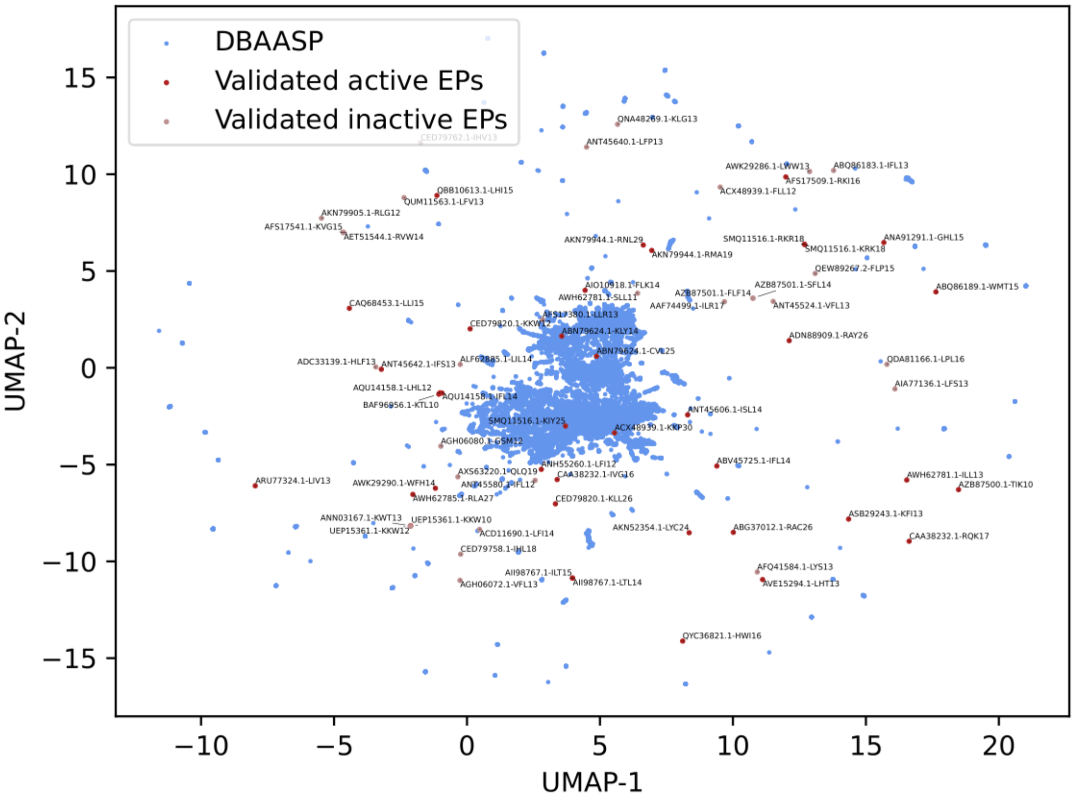

Supplementary Fig. 35 | Sequence space exploration using a similarity matrix containing the 69 encrypted peptides discovered by APEX selected for further experimental validation compared to peptide sequences from DBAASP. We used the same methodology described in **Supplementary Fig. 26** to visualize DBAASP peptide sequences and the 69 EPs discovered by APEX and subsequently selected for synthesis and experimental validation. The 69 EP sequences are highlighted in the scatter plot. We used different colors to show the EPs that were experimentally validated to be active (MIC ≤64 μmol L^{-1}) and inactive antimicrobials against the selected pathogens *in vitro*.

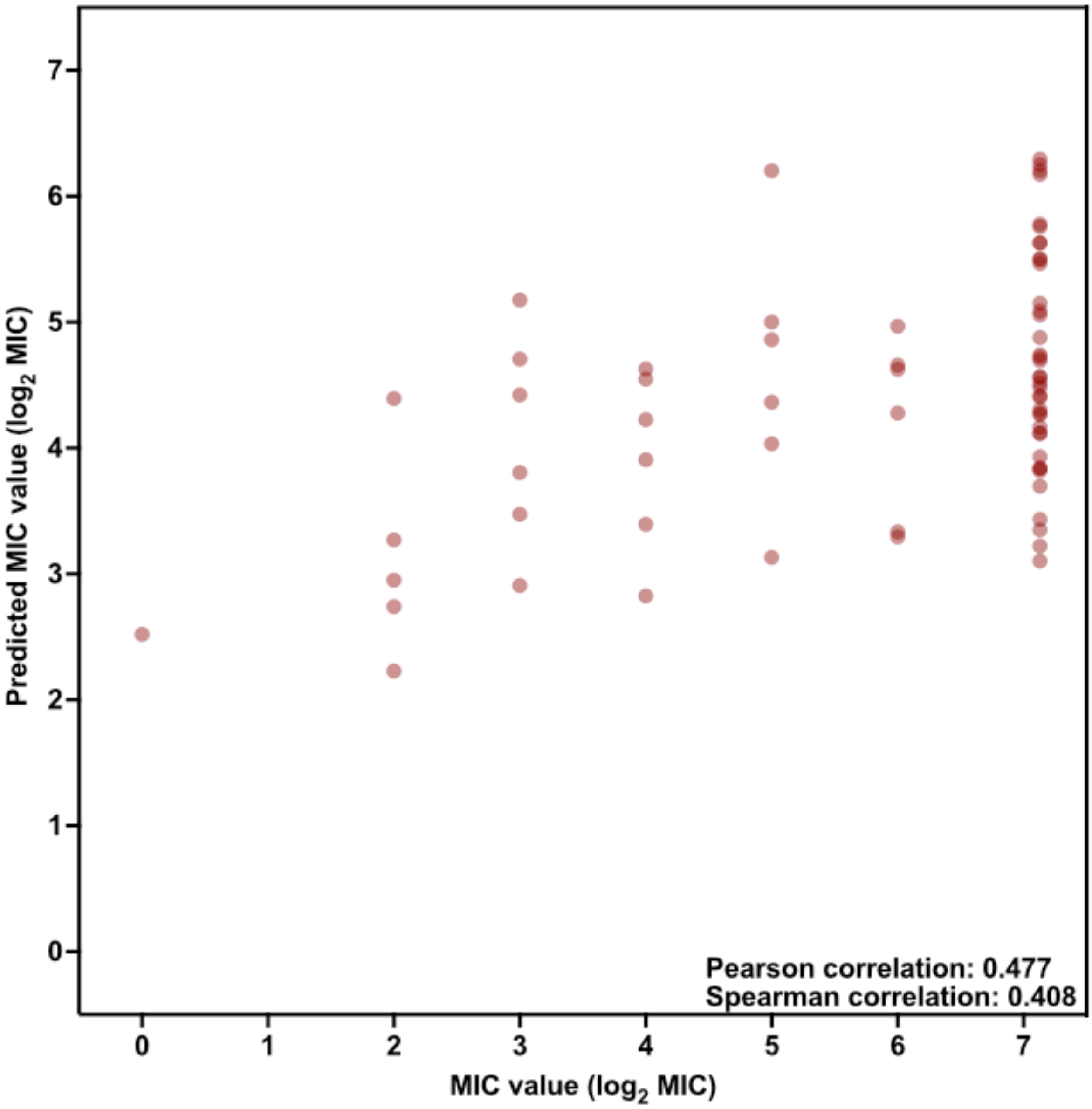

Supplementary Fig. 36 l Predicted vs. experimental MICs for *A. baumannii* ATCC 19606 of the encrypted peptides identified by APEX. Each peptide on the scatter plot is represented by a red circle. Pearson and Spearman correlations values are shown as an inset.

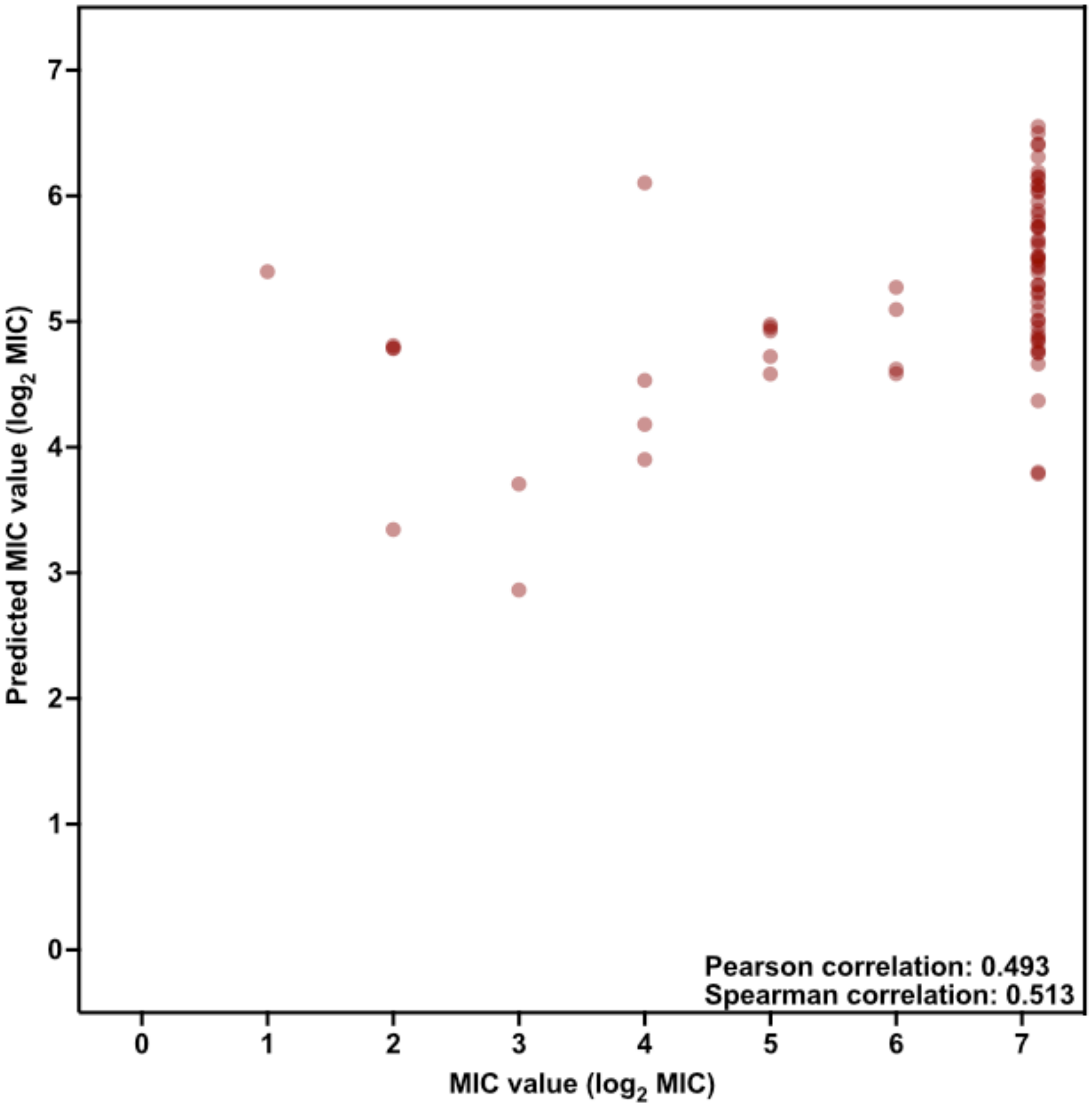

Supplementary Fig. 37 | Predicted vs. experimental MICs for *E. coli* AIC221 of the encrypted peptides identified by APEX. Each peptide on the scatter plot is represented by a red circle. Pearson and Spearman correlations values are shown as an inset.

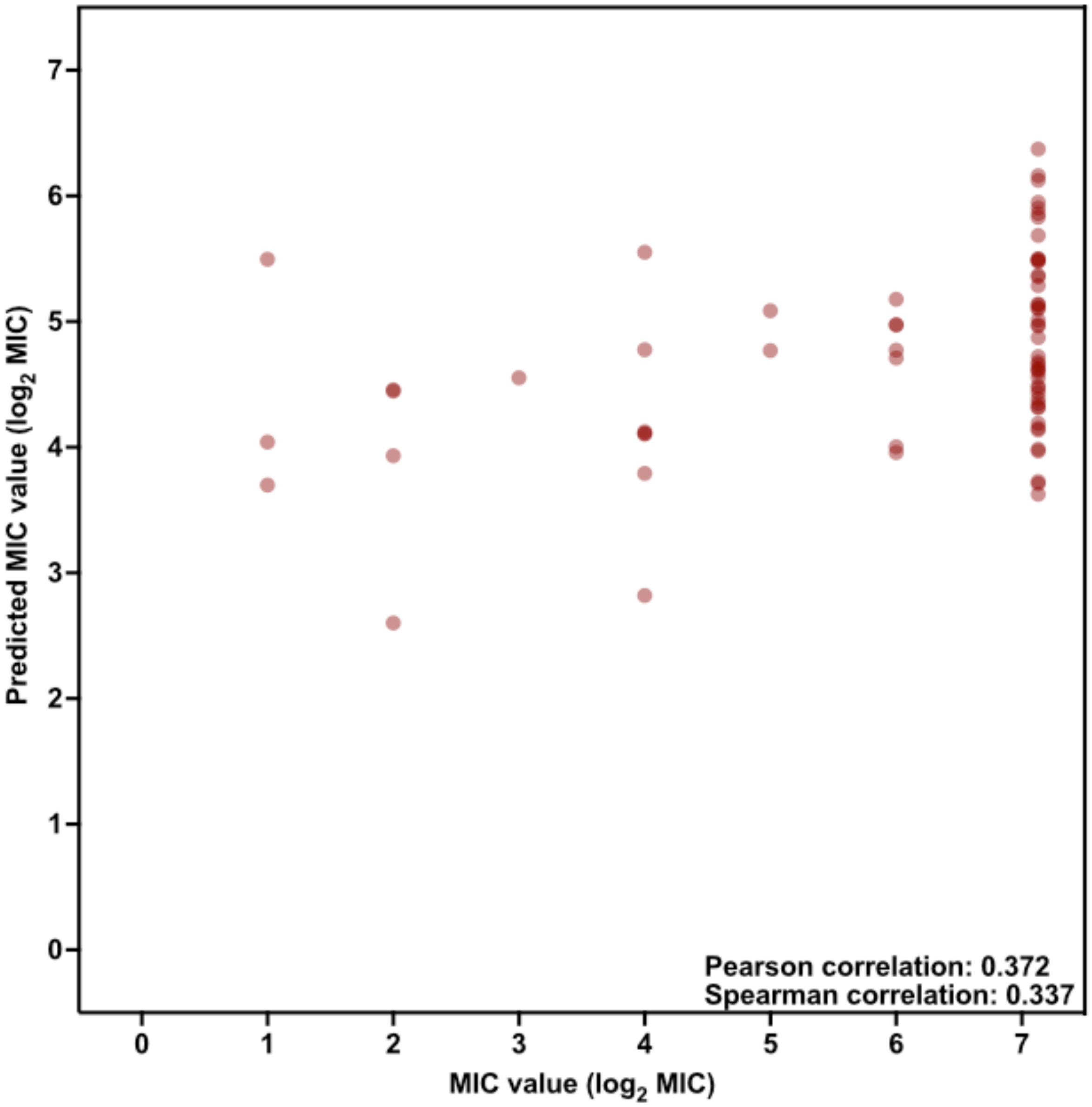

Supplementary Fig. 38 l Predicted *vs* experimental MICs for *E. coli* AIC222 of the encrypted peptides identified by APEX. Each peptide on the scatter plot is represented by a red circle. Pearson and Spearman correlations values are shown as an inset.

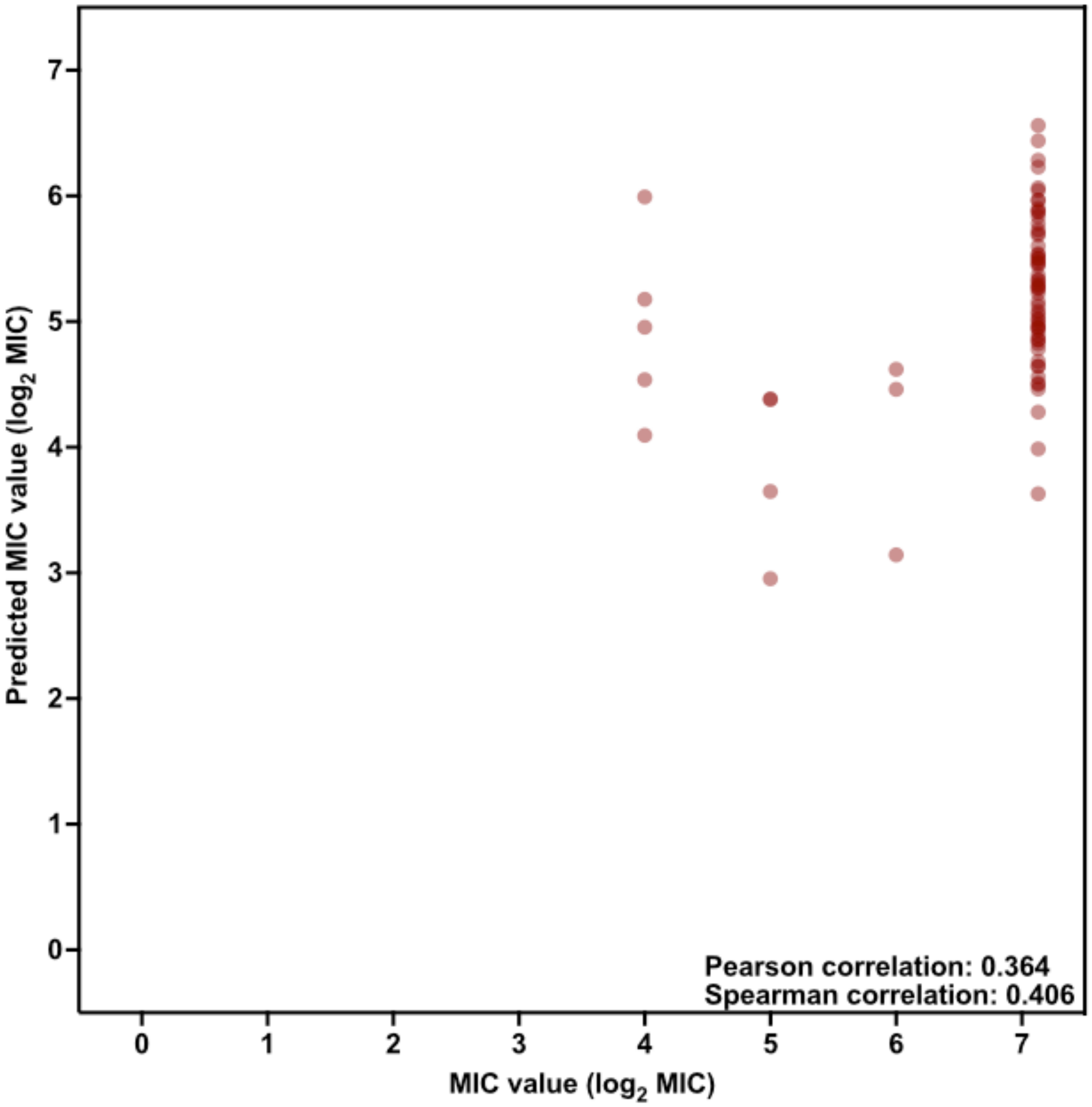

Supplementary Fig. 39 l Predicted vs. experimental MICs for *E. coli* ATCC 11775 of the encrypted peptides identified by APEX. Each peptide on the scatter plot is represented by a red circle. Pearson and Spearman correlations values are shown as an inset.

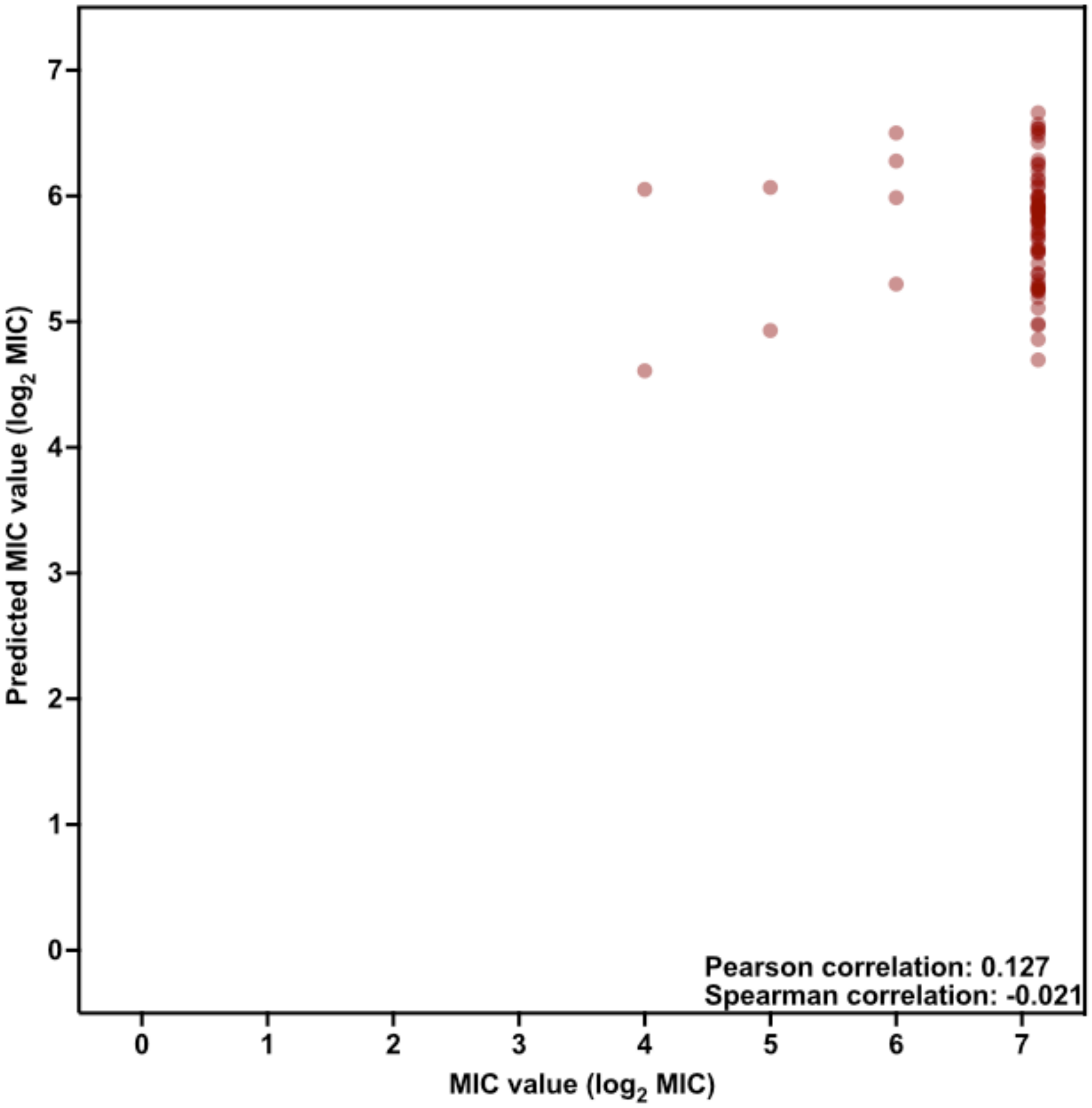

Supplementary Fig. 40 I Predicted vs. experimental MICs for *K. pneumoniae* ATCC 13883 of the encrypted peptides identified by APEX. Each peptide on the scatter plot is represented by a red circle. Pearson and spearman correlations values are shown as an inset.

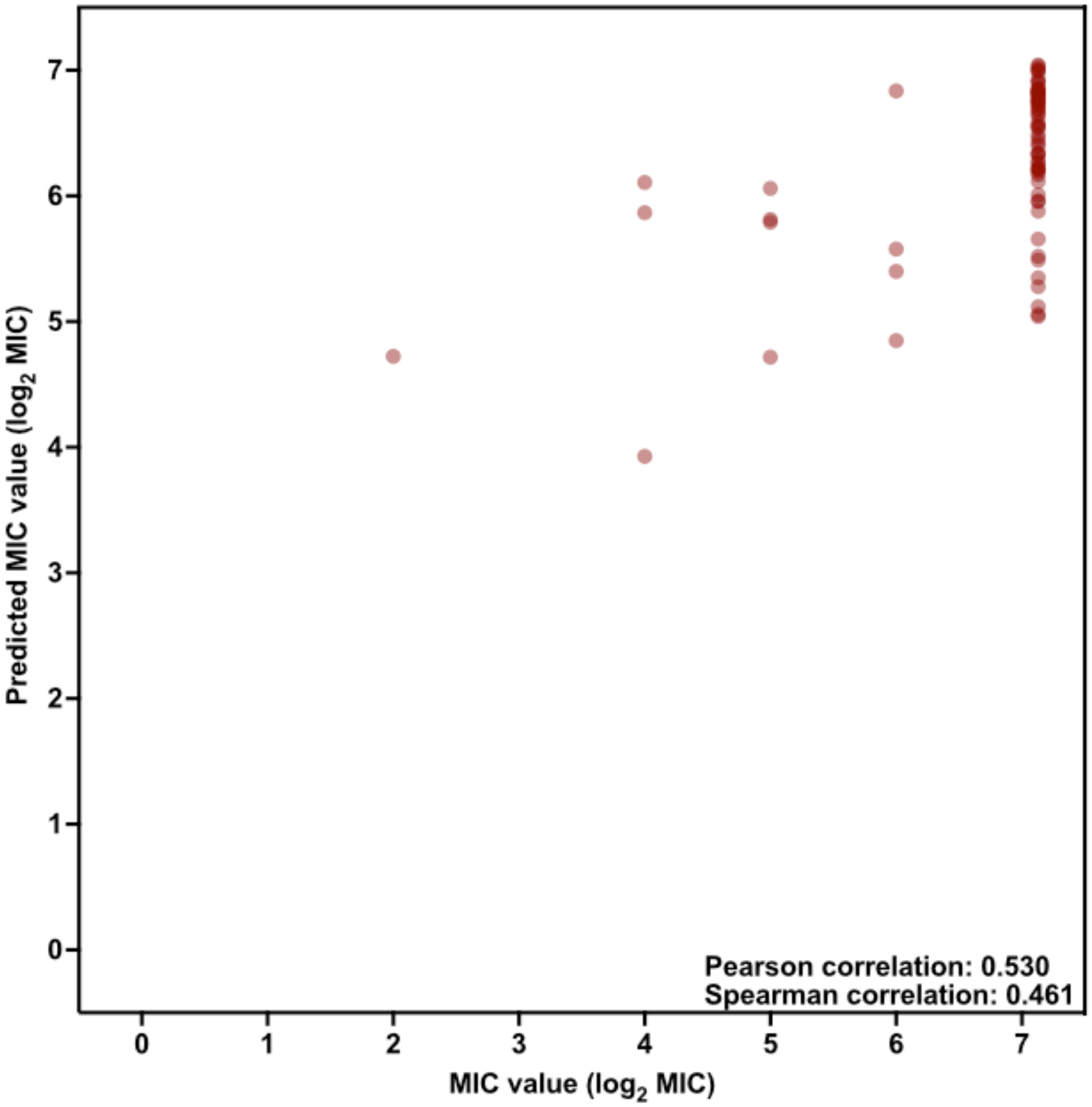

Supplementary Fig. 41 | Predicted vs. experimental MICs for *P. aeruginosa* PA14 of the encrypted peptides identified by APEX. Each peptide on the scatter plot is represented by a red circle. Pearson and spearman correlations values are shown as an inset.

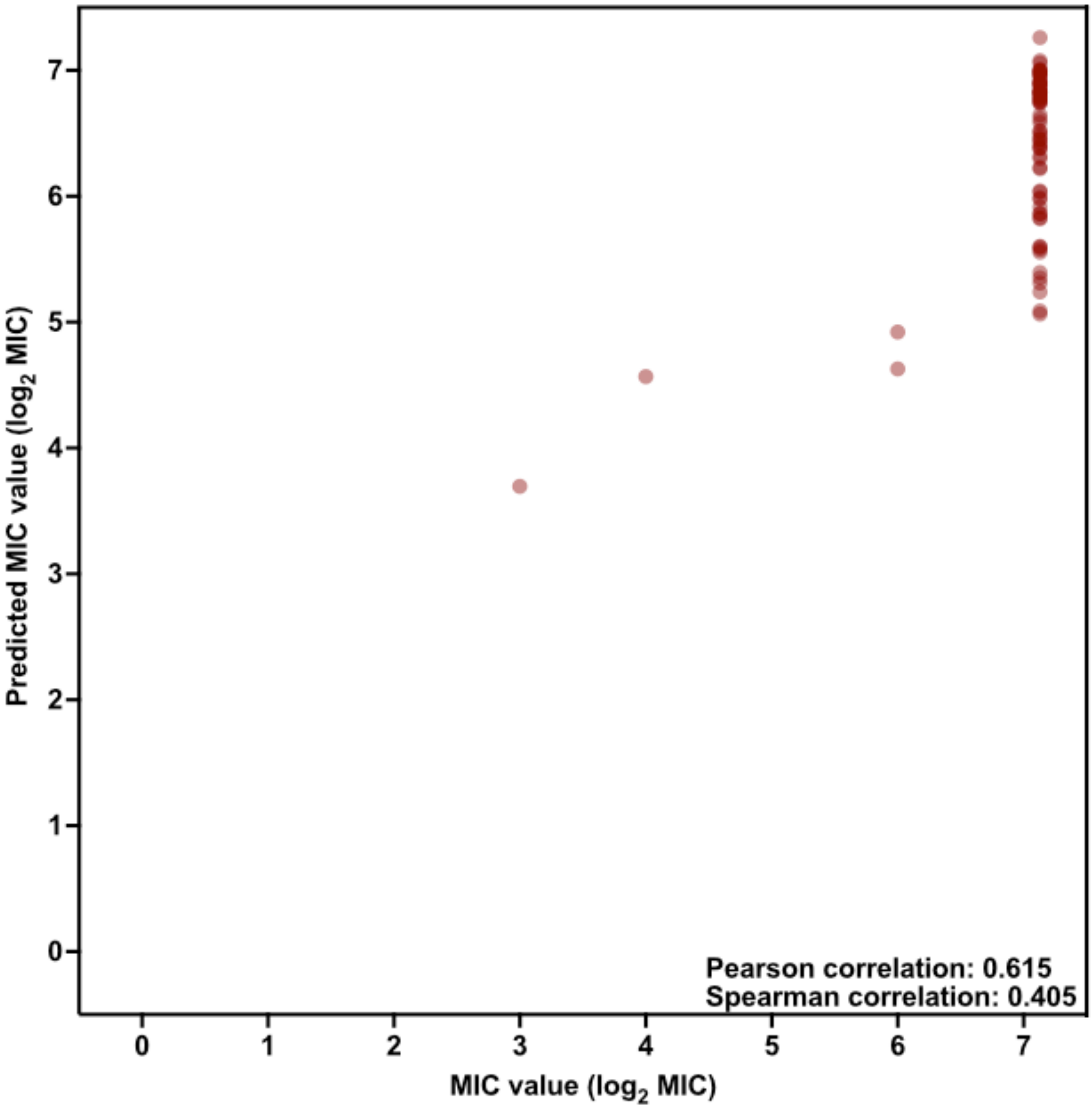

Supplementary Fig. 42 | Predicted vs. experimental MICs for *P. aeruginosa* PAO1 of the encrypted peptides identified by APEX. Each peptide on the scatter plot is represented by a red circle. Pearson and spearman correlations values are shown as an inset.

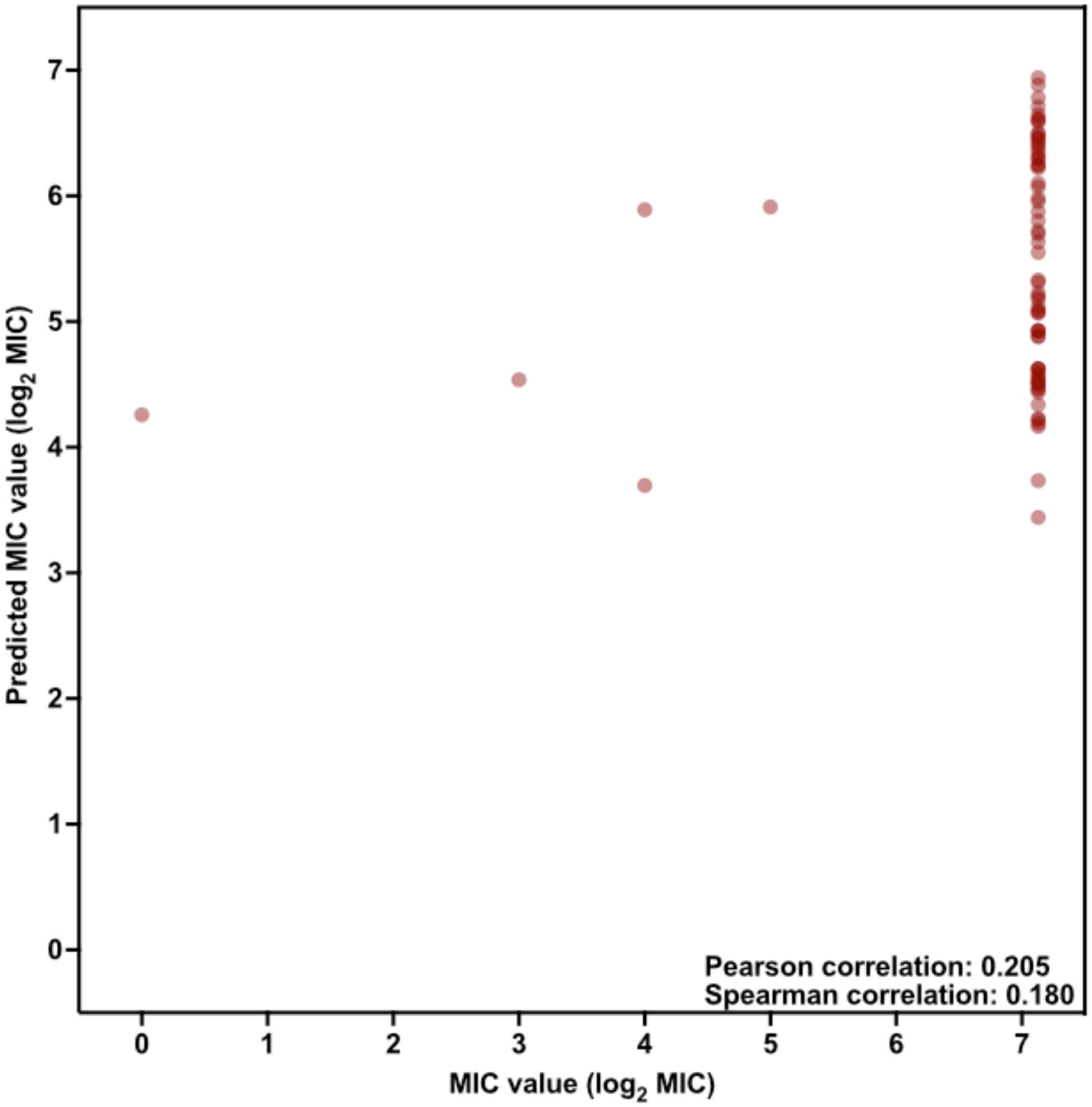

Supplementary Fig. 43 | Predicted vs. experimental MICs for methicillin-resistant *S. aureus* ATCC BAA-1556 of the encrypted peptides identified by APEX. Each peptide on the scatter plot is represented by a red circle. Pearson and spearman correlations values are shown as an inset.

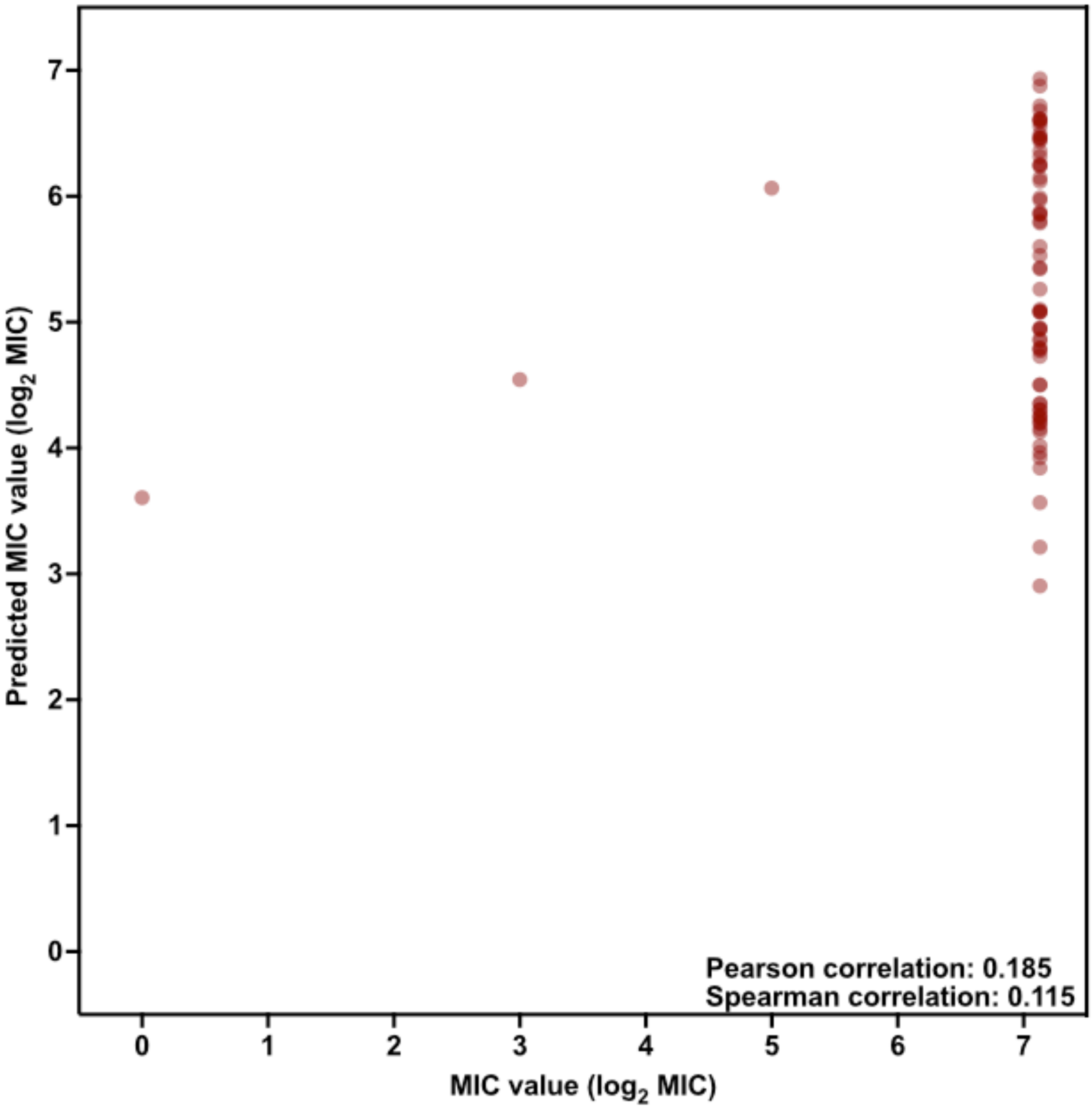

Supplementary Fig. 44 | Predicted vs. experimental MICs for *S. aureus* ATCC 12600 of the encrypted peptides identified by APEX. Each peptide on the scatter plot is represented by a red circle. Pearson and spearman correlations values are shown as an inset.

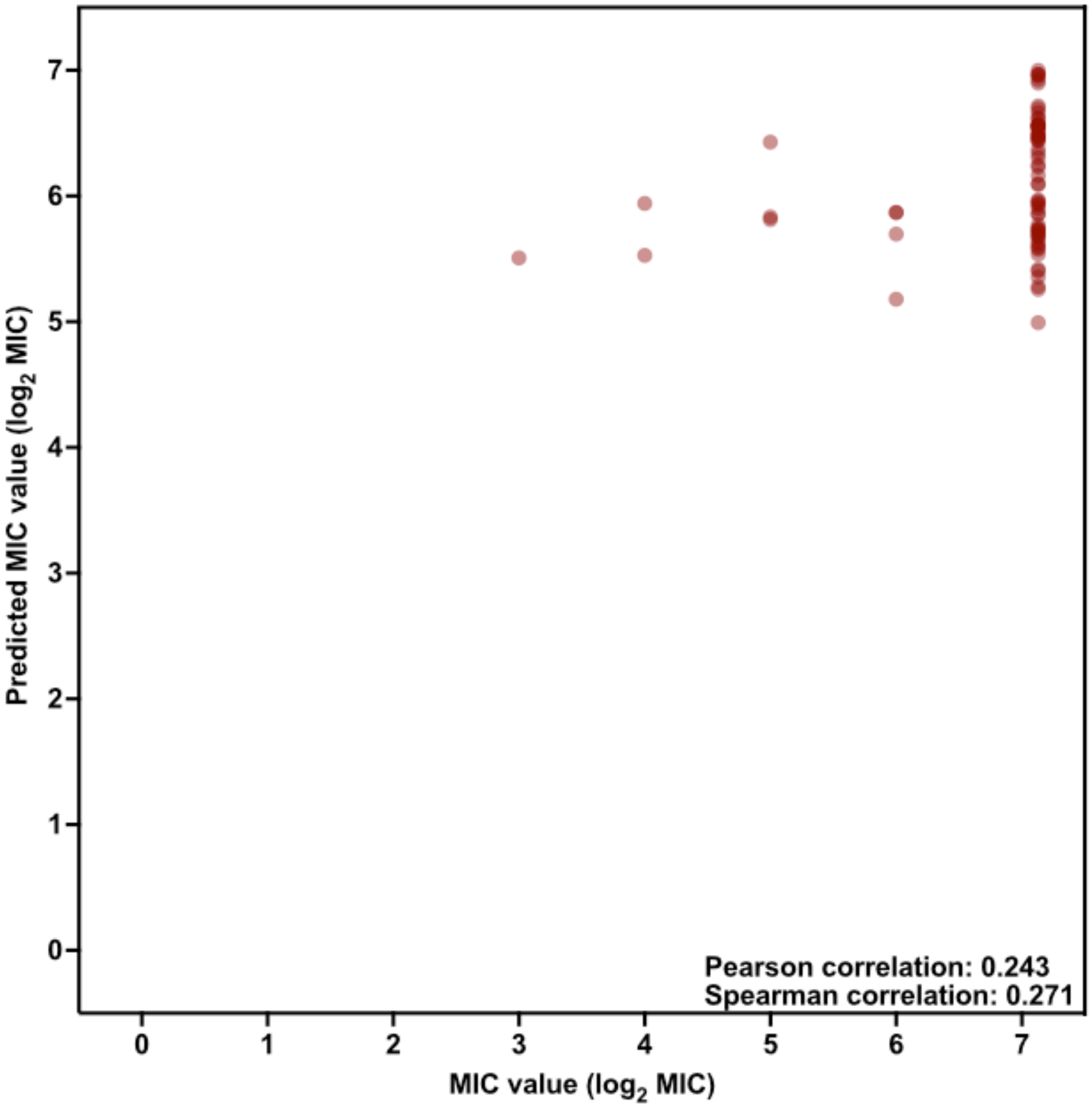

Supplementary Fig. 45 l Predicted vs. experimental MICs for vancomycin-resistant *E. faecalis* ATCC 700802 of the encrypted peptides identified by APEX. Each peptide on the scatter plot is represented by a red circle. Pearson and spearman correlations values are shown as an inset.

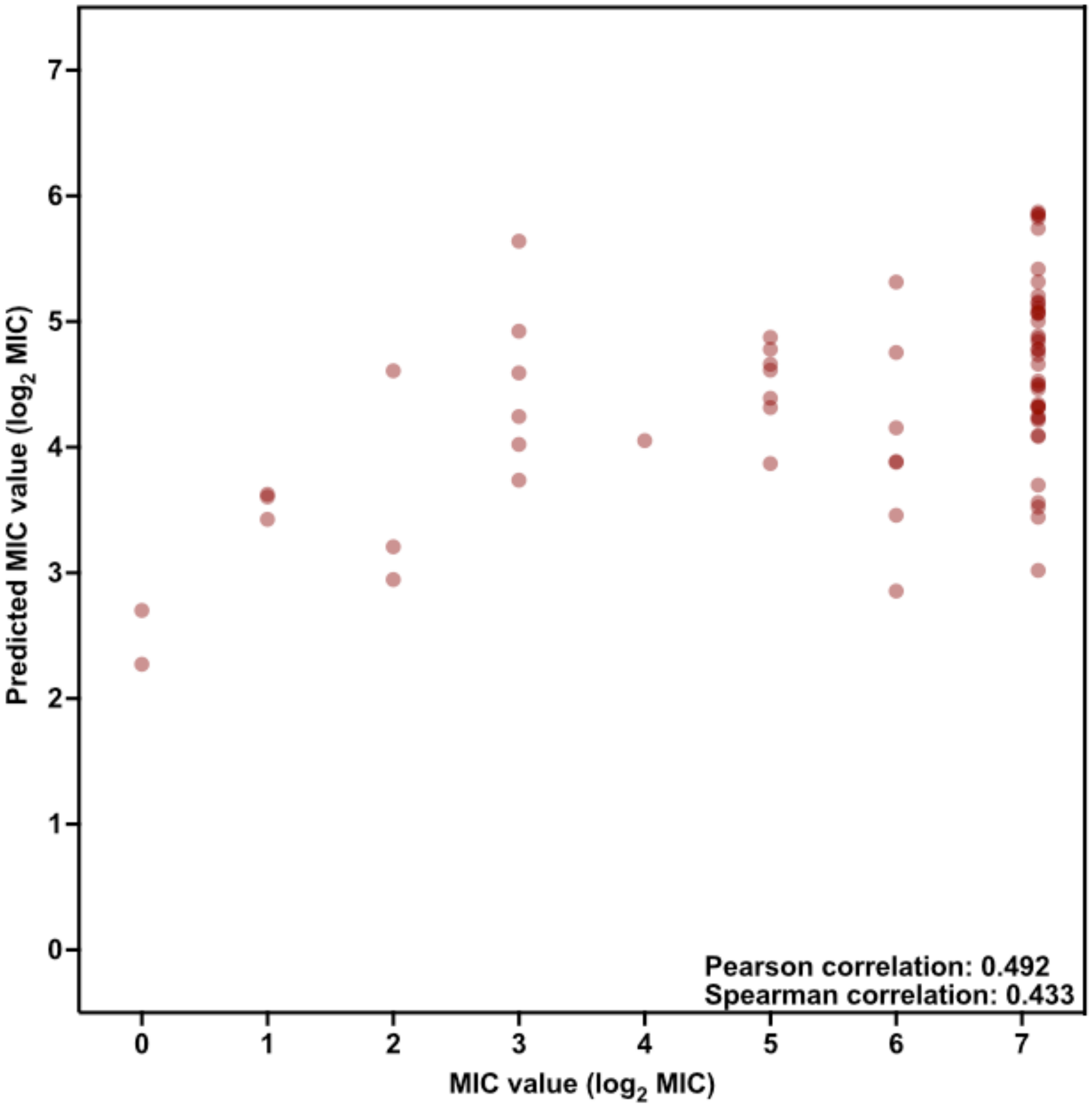

Supplementary Fig. 46 | Predicted vs. experimental MICs for vancomycin-resistant *E. faecium* ATCC 700221 of the encrypted peptides identified by APEX. Each peptide on the scatter plot is represented by a red circle. Pearson and spearman correlations values are shown as an inset.

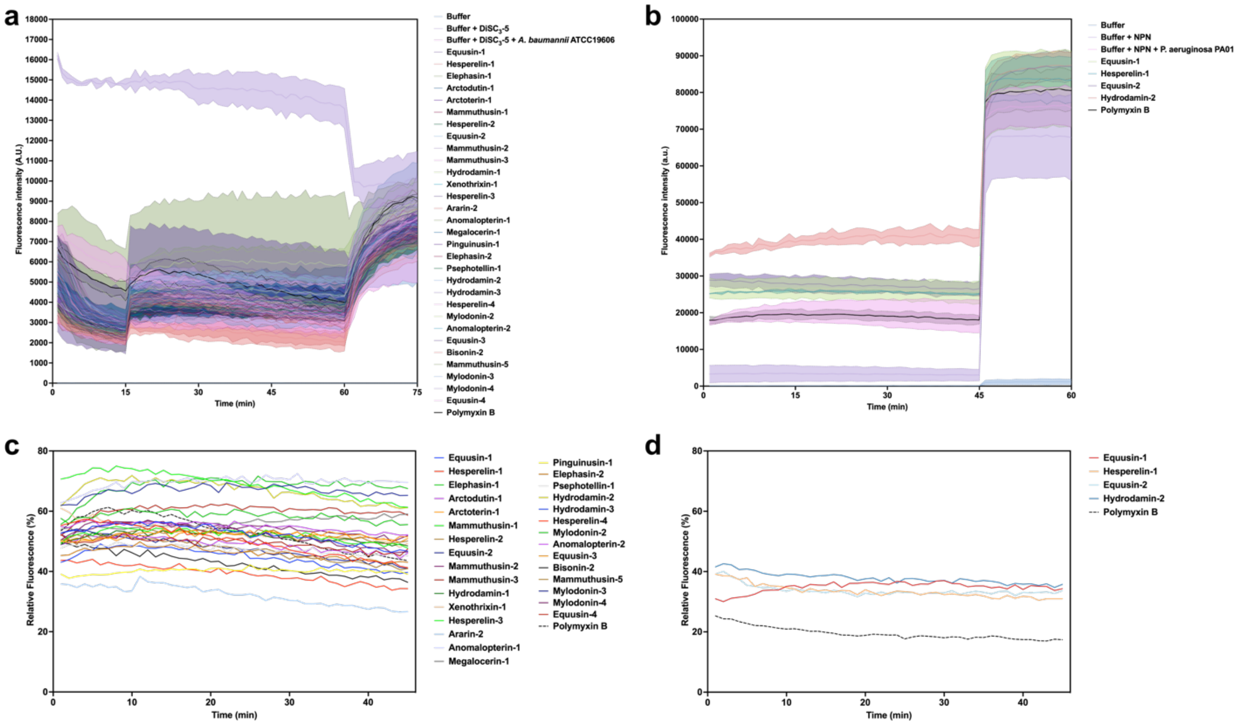

Supplementary Fig. 47 | Cytoplasmic membrane depolarization of *A. baumannii* and *P. aeruginosa* triggered by AEPs and MEPs identified by APEX. (a-b) Depolarization assays with the hydrophobic probe 3,3′-dipropylthiadicarbocyanine iodide [DiSC$_3$-(5)] for all the encrypted peptides (EPs) from extinct organisms that were active against *A. baumannii* ATCC 19606 **(a)** and *P. aeruginosa* PAO1 **(b)**. Polymyxin B was used as positive control, and buffer, buffer with DiSC$_3$-(5), and buffer with DiSC$_3$-(5) and bacteria were used as baseline for fluorescence. The panels show the raw fluorescence intensity data obtained in the experiments. **(c-d)** Relative fluorescence values of the depolarization effect of EPs from extinct organisms compared to the untreated control on the cytoplasmic membranes of **(c)** *A. baumannii* ATCC 19606 and **(d)** *P. aeruginosa* PAO1.

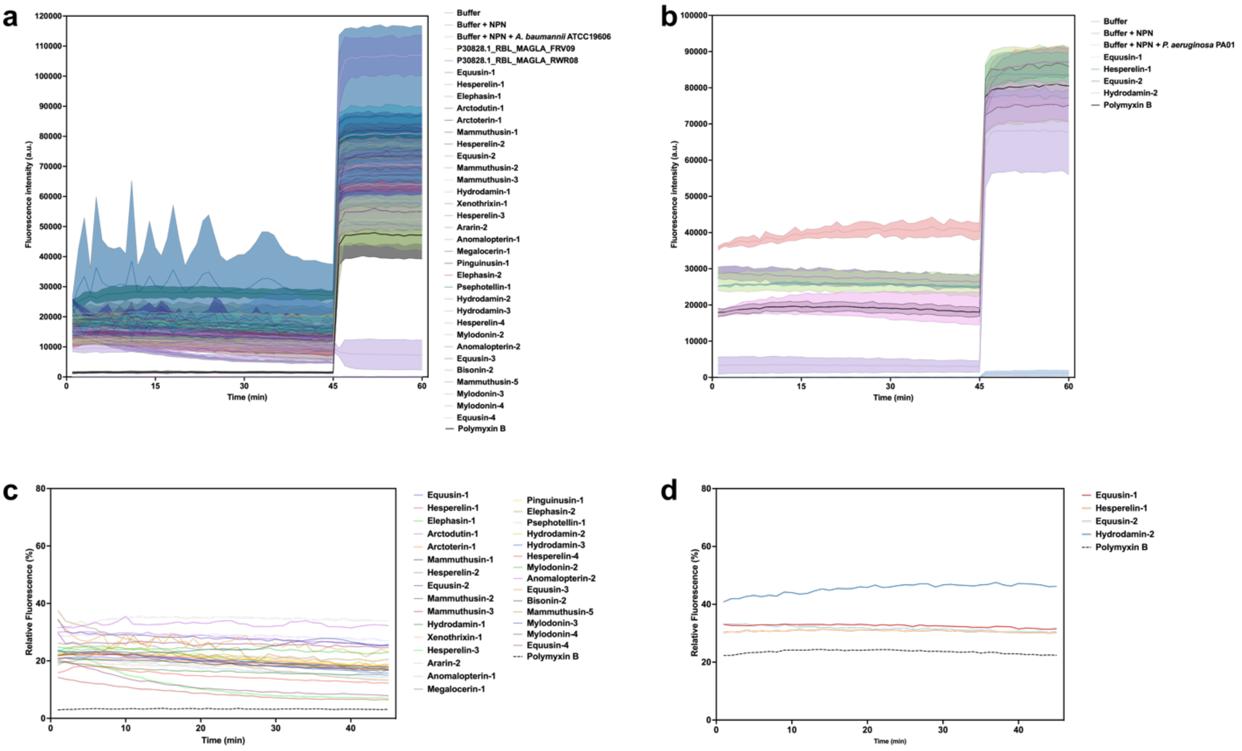

Supplementary Fig. 48 l Outer membrane permeabilization of *A. baumannii* and *P. aeruginosa* cell membranes caused by encrypted peptides from extinct organisms. The probe 1-(N-phenylamino)naphthalene (NPN) was used to detect permeabilization of the outer membrane. **(a-b)** Permeabilization by encrypted peptides (EPs) from extinct organisms active against each one of the strains: on *A. baumannii* ATCC 19606 **(a)** and *P. aeruginosa* PAO1 **(b)**. Polymyxin B was used as positive control. Buffer was used as the baseline for fluorescence, and buffer with NPN, and buffer with NPN and bacteria were used as baseline for fluorescence. The panels show the raw fluorescence intensity data obtained in the experiments. **(c-d)** Relative fluorescence values of the permeabilization effect of encrypted peptides from extinct organisms compared to the untreated control on the outer membranes of **(c)** *A. baumannii* ATCC 19606 and **(d)** *P. aeruginosa* PAO1.

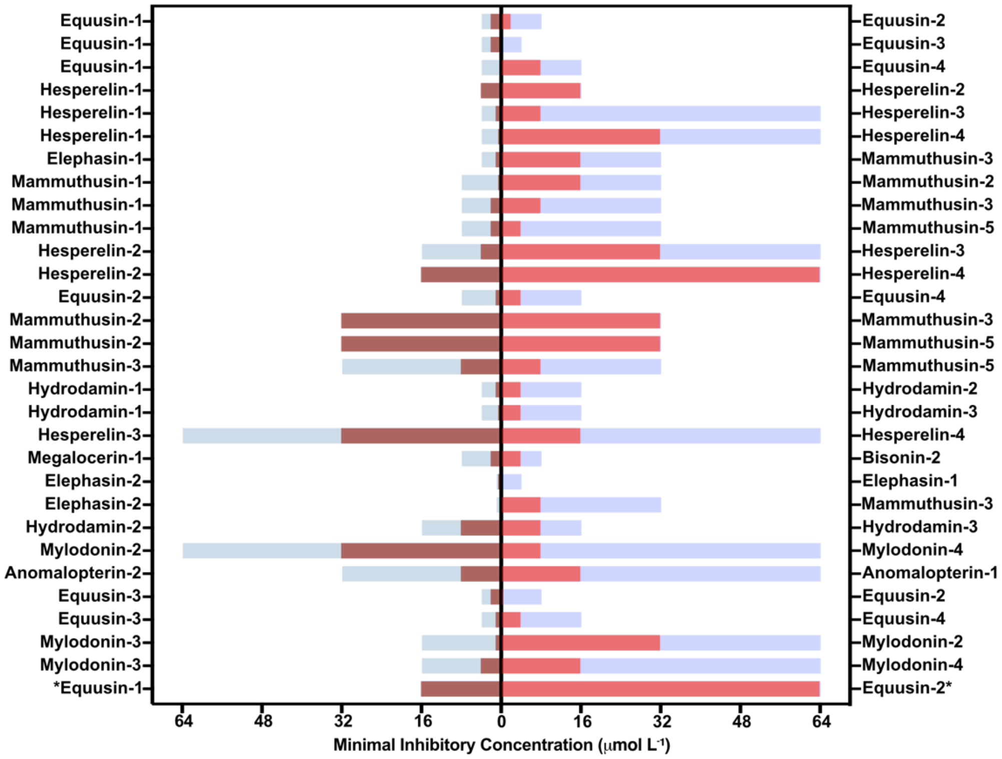

Supplementary Fig. 49 | Synergy between AEPs and MEPs from extinct organisms. Bar plot showing the synergistic interactions between EPs from the same extinct organism against *A. baumannii* and *P. aeruginosa* (*P. aeruginosa* result is indicated with an asterisk). Stacked bars represent the MICs in μmol L^{-1} values in each condition. MICs of the individual peptides are shown in blue and light purple and when in combination, shown in brown and red. Each pair of peptides was placed in the same row for side-by-side comparison of MIC-fold change before and after they were tested in combination.

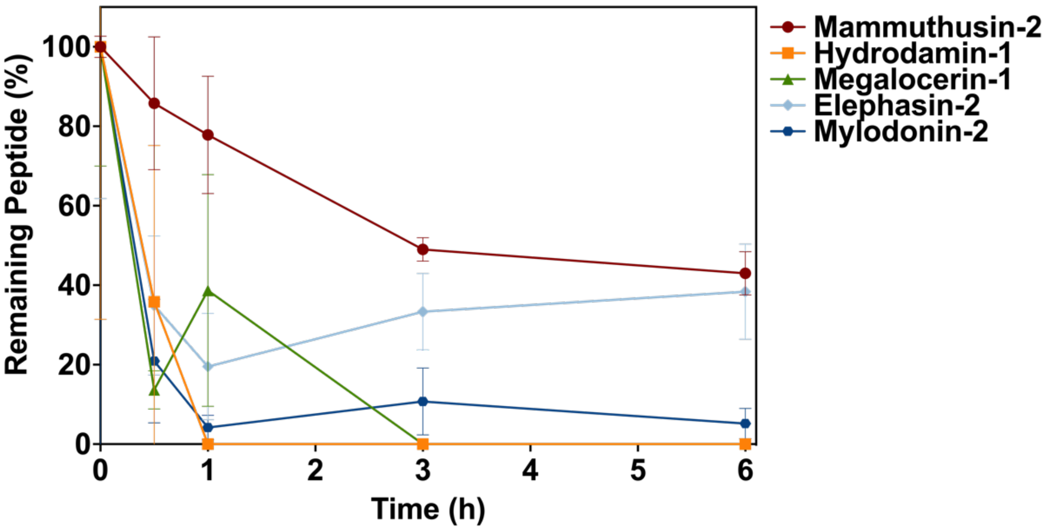

Supplementary Fig. 50 | Resistance to proteolytic degradation assays. The AEPs hydrodamin-1, elephasin-2, and mylodonin-2 and the MEPs mammuthusin-2 and megalocerin-1 were exposed for a total of 6 h to human serum, which contains proteases. Aliquots of the resulting solution were analyzed by liquid chromatography coupled to mass spectrometry. In summary, the AEP elephasin-2 and the MEP mammuthusin-2 exhibited the highest resistance to proteolytic degradation with ~40% peptide remaining after 6 h of exposure. All other AEPs and MEP tested degraded at varying degrees within the duration of the experiment. Experiments were performed in three independent replicates.

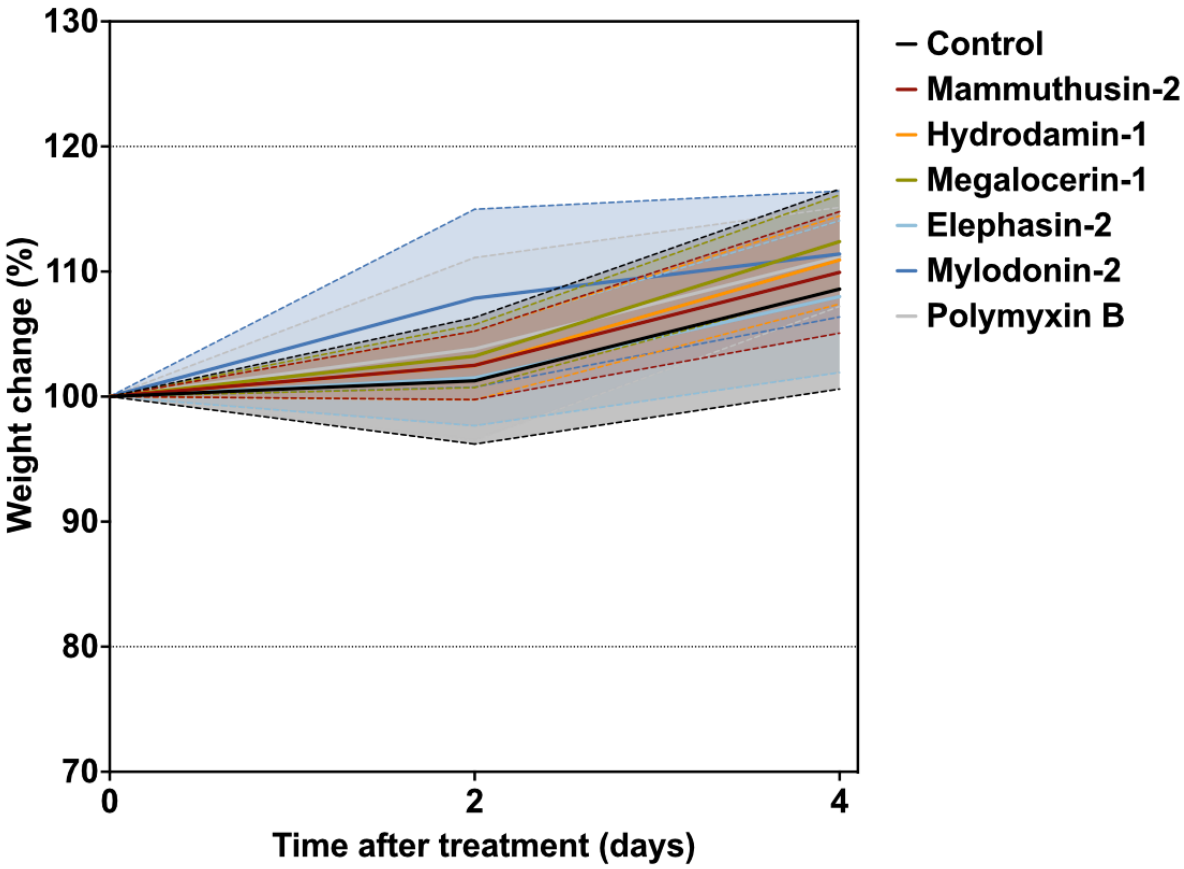

Supplementary Fig. 51 | Weight change monitoring in the skin abscess mouse model infected with *A. baumannii*. Mouse weight was monitored throughout the duration of the skin abscess assay (4 days total) to rule out potential toxic effects of the bacterial load and the encrypted peptides.

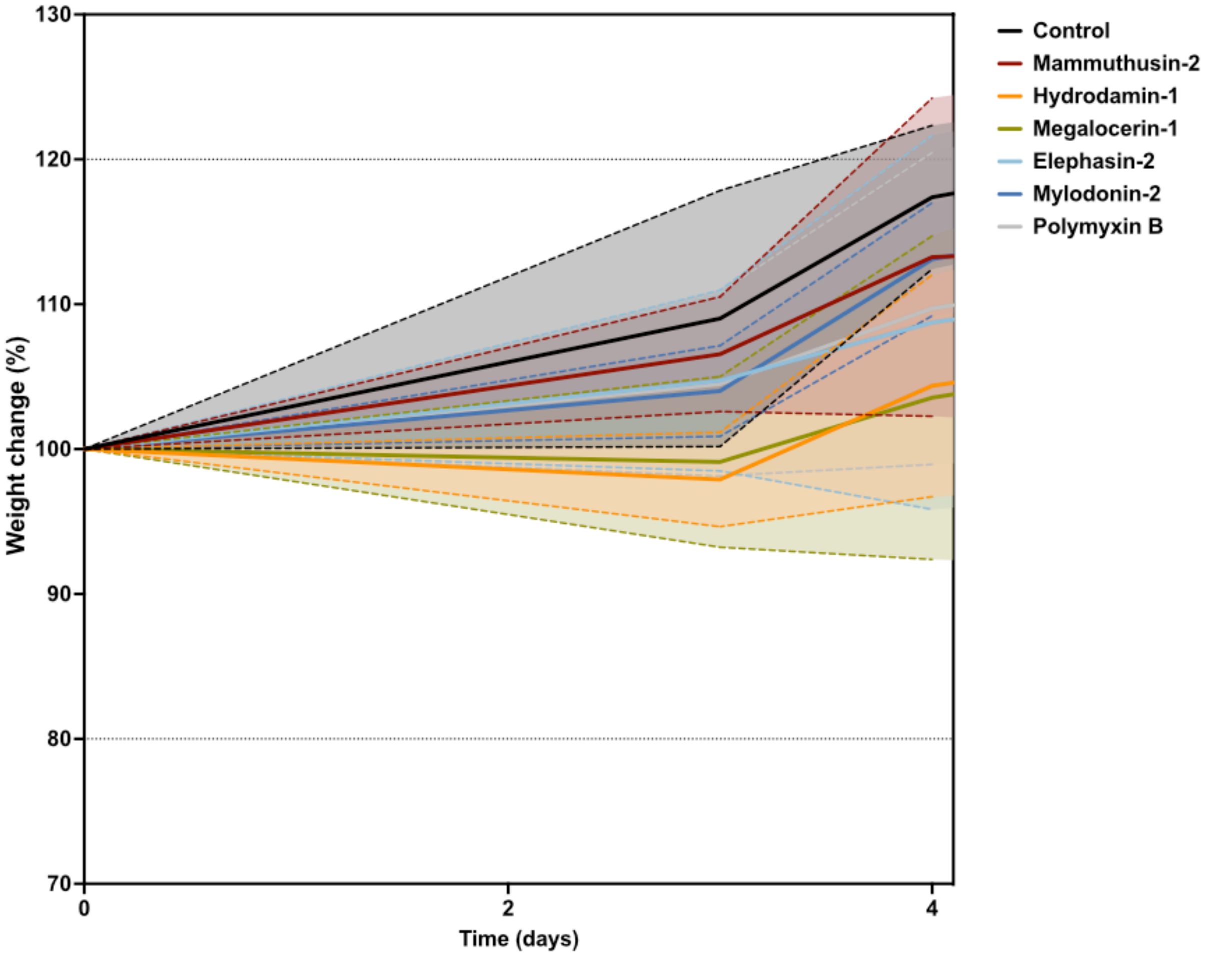

Supplementary Fig. 52 | Weight change monitoring in the thigh mouse model infected with *A. baumannii*. Mouse weight was monitored throughout the duration of the thigh infection (8 days total) to rule out the potentially toxic effects of bacterial load or the encrypted peptides.

Supplementary tables

Supplementary Table 1 I Hyperparameter ranges explored for APEX.

**Hyperparameters**	**number of RNN layers**	**n**	**m**	**λ_l2**	**λ_(multitask_constraint)**	**λ_BCE**
Hyperparameter range	{1, 2, 3}	{128, 256}	{512, 1024, 2048}	{1e-5, 1e-6}	{0.1, 0.01, 0.001, 0.0}	{1.0, 0.1, 0.0}

Supplementary Table 2 I Hyperparameter ranges searched for elastic net.

**Elastic Net hyperparameter**	**alpha**
Hyperparameter range	{1.0, 0.5, 0.1, 0.05, 0.01, 0.005, 0.001, 0.0005, 0.0001}

Supplementary Table 3 I Hyperparameter ranges searched for linear support vector machine.

**Linear support vector regression hyperparameter**	**C**
Hyperparameter range	{1, 10, 20, 30, …, 1000}

Supplementary Table 4 I Hyperparameter ranges searched for tree-based models.

**Random forest, gradient boosting decision tree and, extra-trees regressor hyperparameter hyperparameters**	**Num estimators**	**Max depth**
Hyperparameter range	{128, 256, 512, 1024}	{8, 16, 32, 64}

Supplementary Table 5 I R-squared of various ML models on CV set. We divided our in-house peptide dataset into a CV set for hyperparameter tuning and ML model selection, and an independent dataset to evaluate prediction performance. The Supplementary Table hows the average species-specific prediction performance of 5-fold CV in terms of R-squared for various ML models on the CV set. RF: random forest; GBDT: gradient boosting decision tree; ExtraTree: extra-tree regressor; ElasticNet: elastic net; LinearSVR: linear support vector regression.

Strain	Ensemble APEX v1	Single APEX	RF	GBDT	ExtraTree	ElasticNet	LinearSVR
E. coli ATCC11775	0.588229	0.519899	0.494097	0.446439	0.434624	0.417793	0.128045
P. aeruginosa PAO1	0.520804	0.458351	0.490921	0.401458	0.430824	0.41167	-0.07728
P. aeruginosa PA14	0.461245	0.415884	0.402854	0.327131	0.38079	0.360004	0.02522
S. aureus ATCC12600	0.519007	0.443486	0.388353	0.348654	0.267847	0.271189	0.017144
E. coli AIC221	0.574544	0.527619	0.46737	0.471732	0.428688	0.351852	0.142403
E. coli AIC222	0.638583	0.578155	0.50825	0.497999	0.445026	0.408777	0.159808
K. pneumoniae ATCC13883	0.215734	0.078356	0.093001	0.025148	-0.17556	0.017496	-0.36587
A. baumannii ATCC19606	0.675531	0.597275	0.577164	0.581414	0.559864	0.420903	0.281722
Methicillin-resistant *S. aureus* ATCC BAA-1556	0.539041	0.442817	0.346027	0.355517	0.15017	0.237193	-0.2676
Vancomycin-resistant *E. faecalis* ATCC700802	-0.14859	-0.46429	-0.03412	-0.56905	-0.05681	-0.01311	-0.09197
Vancomycin-resistant *E. faecium* ATCC700221	0.615554	0.460972	0.422509	0.347594	0.345839	0.441332	0.385905
**Average**	**0.472699**	**0.368956**	**0.377857**	**0.294003**	**0.291936**	**0.302282**	**0.030685**

Supplementary Table 6 | Pearson correlation of various ML models on CV set. We divided our in-house peptide dataset into a CV set for hyperparameter tuning and ML model selection, and an independent dataset to evaluate prediction performance. The Supplementary Table hows the average species-specific prediction performance of 5-fold CV in terms of Pearson correlation for various ML models on the CV set. RF: random forest; GBDT: gradient boosting decision tree; ExtraTree: extra-tree regressor; ElasticNet: elastic net; LinearSVR: linear support vector regression.

**Strain**	**Ensemble APEX v1**	**Single APEX**	**RF**	**GBDT**	**ExtraTree**	**ElasticNet**	**LinearSVR**
E. coli ATCC11775	0.767053	0.722816	0.703998	0.691083	0.661328	0.646422	0.556482
P. aeruginosa PAO1	0.72198	0.683082	0.701559	0.673932	0.656995	0.644568	0.450686
P. aeruginosa PA14	0.679904	0.654815	0.635966	0.598879	0.619795	0.606475	0.449355
S. aureus ATCC12600	0.720695	0.672914	0.623975	0.609784	0.548119	0.523365	0.469692
E. coli AIC221	0.758512	0.728434	0.688737	0.6902	0.65761	0.594226	0.490013
E. coli AIC222	0.799682	0.761297	0.715085	0.710545	0.667721	0.640531	0.52739
K. pneumoniae ATCC13883	0.477322	0.401615	0.319796	0.332036	0.155075	0.228322	0.250313
A. baumannii ATCC19606	0.822192	0.774607	0.761093	0.765975	0.749168	0.664209	0.58983
Methicillin-resistant *S. aureus* ATCC BAA-1556	0.734576	0.680613	0.589402	0.620643	0.500931	0.497521	0.424084
Vancomycin-resistant *E. faecalis* ATCC700802	0.096459	0.056318	0.029826	0.026884	0.029424	-0.15356	0.048211
Vancomycin-resistant *E. faecium* ATCC700221	0.784896	0.697111	0.655262	0.617407	0.600566	0.674514	0.622007
**Average**	**0.669388**	**0.621238**	**0.584063**	**0.576124**	**0.531521**	**0.506053**	**0.44346**

Supplementary Table 7 | Spearman correlation of various ML models on CV set. We divided our in-house peptide dataset into a CV set for hyperparameter tuning and ML model selection, and an independent dataset to evaluate prediction performance. The Supplementary Table hows the average species-specific prediction performance of 5-fold CV in terms of Spearman correlation for various ML models on the CV set. RF: random forest; GBDT: gradient boosting decision tree; ExtraTree: extra-tree regressor; ElasticNet: elastic net; LinearSVR: linear support vector regression.

**Strain**	**Ensemble APEX v1**	**Single APEX**	**RF**	**GBDT**	**ExtraTree**	**ElasticNet**	**LinearSVR**
E. coli ATCC11775	0.686485	0.66331	0.622101	0.603803	0.571779	0.572859	0.446956
P. aeruginosa PAO1	0.636473	0.611234	0.606478	0.602546	0.579222	0.54594	0.368697
P. aeruginosa PA14	0.658294	0.625266	0.611538	0.592355	0.596603	0.569181	0.405066
S. aureus ATCC12600	0.546967	0.484758	0.433656	0.468351	0.297889	0.408671	0.260263
E. coli AIC221	0.733874	0.719559	0.673859	0.653163	0.681991	0.617147	0.469486
E. coli AIC222	0.737407	0.720051	0.672798	0.6632	0.626752	0.607942	0.452432
K. pneumoniae ATCC13883	0.527278	0.486858	0.260868	0.430102	0.058973	0.333547	0.159662
A. baumannii ATCC19606	0.726801	0.703749	0.687577	0.676496	0.696205	0.635361	0.510874
Methicillin-resistant *S. aureus* ATCC BAA-1556	0.525053	0.489835	0.462725	0.460328	0.309507	0.428855	0.348216
Vancomycin-resistant *E. faecalis* ATCC700802	0.107576	0.047122	0.141981	0.079331	0.169632	-0.08813	0.200453
Vancomycin-resistant *E. faecium* ATCC700221	0.646947	0.569296	0.484717	0.524636	0.444265	0.531174	0.468919
**Average**	**0.593923**	**0.556458**	**0.514391**	**0.523119**	**0.457529**	**0.469322**	**0.371911**

Supplementary Table 8 | R-squared of single APEX variants on CV set. We divided our in-house peptide dataset into a CV set for hyperparameter tuning and ML model selection, and an independent dataset to evaluate prediction performance. The Supplementary Table hows the average species-specific prediction performance of 5-fold CV in terms of R-squared for various APEX variants, including the original APEX (*i.e.*, single APEX), APEX without multitask constraint, APEX without using public AMP data during training, and APEX without multitask constraint and public AMP data during training.

**Strain**	**Single APEX**	**Single APEX without multitask constraint**	**Single APEX without public AMP data**	**Single APEX without multitask constraint and public AMP data**
E. coli ATCC11775	0.519899	0.540226	0.515468	0.504094
P. aeruginosa PAO1	0.458351	0.411795	0.418864	0.328494
P. aeruginosa PA14	0.415884	0.33248	0.386427	0.253563
S. aureus ATCC12600	0.443486	0.418941	0.378119	0.391915
E. coli AIC221	0.527619	0.509886	0.468906	0.459699
E. coli AIC222	0.578155	0.586917	0.547914	0.559102
K. pneumoniae ATCC13883	0.078356	0.110211	0.12016	0.078548
A. baumannii ATCC19606	0.597275	0.597209	0.554144	0.57716
Methicillin-resistant *S. aureus* ATCC BAA-1556	0.442817	0.500468	0.432583	0.45067
Vancomycin-resistant *E. faecalis* ATCC700802	-0.46429	-0.18286	-0.48424	0.021012
Vancomycin-resistant *E. faecium* ATCC700221	0.460972	0.512912	0.479714	0.52491
**Average**	**0.368956**	**0.39438**	**0.347096**	**0.377197**

Supplementary Table 9 | Pearson correlation of single APEX variants on CV set. We divided our in-house peptide dataset into a CV set for hyperparameter tuning and ML model selection, and an independent dataset to evaluate prediction performance. The Supplementary Table hows the average species-specific prediction performance of 5-fold CV in terms of Pearson correlation for various APEX variants, including the original APEX (i.e., single APEX), APEX without multitask constraint, APEX without using public AMP data during training, and APEX without multitask constraint and public AMP data during training.

**Strain**	**Single APEX**	**Single APEX without multitask constraint**	**Single APEX without public AMP data**	**Single APEX without multitask constraint and public AMP data**
E. coli ATCC11775	0.722816	0.745399	0.72278	0.724131
P. aeruginosa PAO1	0.683082	0.660926	0.657109	0.616645
P. aeruginosa PA14	0.654815	0.603326	0.633283	0.563075
S. aureus ATCC12600	0.672914	0.673138	0.62627	0.650051
E. coli AIC221	0.728434	0.716857	0.691317	0.687469
E. coli AIC222	0.761297	0.76824	0.744002	0.752025
K. pneumoniae ATCC13883	0.401615	0.419686	0.408857	0.390867
A. baumannii ATCC19606	0.774607	0.776853	0.749409	0.767272
Methicillin-resistant *S. aureus* ATCC BAA-1556	0.680613	0.717745	0.661616	0.67801
Vancomycin-resistant *E. faecalis* ATCC700802	0.056318	0.157377	0.010612	0.275715
Vancomycin-resistant *E. faecium* ATCC700221	0.697111	0.721689	0.699877	0.734134
**Average**	**0.621238**	**0.63284**	**0.600467**	**0.621763**

Supplementary Table 10 | Spearman correlation of single APEX variants on CV set. We divided our in-house peptide dataset into a CV set for hyperparameter tuning and ML model selection, and an independent dataset to evaluate prediction performance. The Supplementary Table hows the average species-specific prediction performance of 5-fold CV in terms of Spearman correlation for various APEX variants, including the original APEX (i.e., single APEX), APEX without multitask constraint, APEX without using public AMP data during training, and APEX without multitask constraint and public AMP data during training.

**Strain**	**Single APEX**	**Single APEX without multitask constraint**	**Single APEX without public AMP data**	**Single APEX without multitask constraint and public AMP data**
E. coli ATCC11775	0.66331	0.624219	0.6493	0.640146
P. aeruginosa PAO1	0.611234	0.556474	0.576812	0.575338
P. aeruginosa PA14	0.625266	0.578997	0.609458	0.544803
S. aureus ATCC12600	0.484758	0.478392	0.456472	0.47019
E. coli AIC221	0.719559	0.659801	0.683423	0.661957
E. coli AIC222	0.720051	0.686905	0.691819	0.688436
K. pneumoniae ATCC13883	0.486858	0.370328	0.435828	0.396926
A. baumannii ATCC19606	0.703749	0.687933	0.703927	0.705705
Methicillin-resistant *S. aureus* ATCC BAA-1556	0.489835	0.469302	0.498565	0.508692
Vancomycin-resistant *E. faecalis* ATCC700802	0.047122	0.150755	0.055165	0.242589
Vancomycin-resistant *E. faecium* ATCC700221	0.569296	0.586361	0.577632	0.59948
**Average**	**0.556458**	**0.53177**	**0.539855**	**0.548569**

Supplementary Table 11 | Hyperparameters of the top eight APEX models ranked by R-squared on CV set.

Top eight APEX models	number of RNN layers	n	m	λ_l2	λ_(multitask_constraint)	λ_BCE
1	3	128	2048	1.00E-05	0.1	1
2	3	256	2048	1.00E-06	0.1	1
3	2	128	512	1.00E-05	0.01	1
4	3	128	512	1.00E-05	0.001	1
5	2	128	2048	1.00E-06	0	1
6	3	256	512	1.00E-06	0	1
7	2	128	2048	1.00E-05	0.01	1
8	2	256	2048	1.00E-06	0.1	1

Supplementary Table 12 | R-squared of various ML models on independent set. We divided our in-house peptide dataset into a CV set for hyperparameter tuning and ML model selection, and an independent dataset to evaluate prediction performance. The Supplementary Table hows the species-specific prediction performance in terms of R-squared for various ML models that were trained on the CV set and evaluated on the independent set. RF: random forest; GBDT: gradient boosting decision tree; ExtraTree: extra-tree regressor; ElasticNet: elastic net; LinearSVR: linear support vector regression.

Strain	Ensemble APEX v2	Ensemble APEX v1	Single APEX	RF	GBDT	ExtraTree	ElasticNet	LinearSVR
E. coli ATCC11775	0.718159	0.691395	0.668556	0.63738	0.648213	0.551901	0.520619	0.401562
P. aeruginosa PAO1	0.58922	0.550826	0.542209	0.585178	0.437675	0.583725	0.444805	-0.30991
P. aeruginosa PA14	0.577267	0.544865	0.523449	0.525311	0.469315	0.541576	0.464316	0.082555
S. aureus ATCC12600	0.654147	0.65415	0.613936	0.498575	0.496227	0.286083	0.309557	0.254665
E. coli AIC221	0.454362	0.423644	0.448303	0.380448	0.35911	0.338844	0.330079	-0.07492
E. coli AIC222	0.659494	0.642316	0.636876	0.540003	0.476026	0.456006	0.400548	0.208218
K. pneumoniae ATCC13883	0.388908	0.380317	0.439253	0.134236	0.174184	-0.16017	0.095197	-0.17792
A. baumannii ATCC19606	0.694184	0.697003	0.687141	0.664341	0.625813	0.627206	0.541836	0.250524
Methicillin-resistant *S. aureus* ATCC BAA-1556	0.725662	0.739452	0.60538	0.578394	0.587113	0.221953	0.325214	-0.12777
Vancomycin-resistant *E. faecalis* ATCC700802	0.002173	-0.07186	0.304024	-0.04891	-0.09351	-0.09296	-0.01733	-0.01604
Vancomycin-resistant *E. faecium* ATCC700221	0.538558	0.472891	0.514716	0.448163	0.375496	0.28212	0.34944	0.130954
**Average**	**0.545648**	**0.520455**	**0.543986**	**0.449374**	**0.414151**	**0.330572**	**0.342208**	**0.056538**

Supplementary Table 13 | Pearson correlation of various ML models on independent set. We divided our in-house peptide dataset into a CV set for hyperparameter tuning and ML model selection, and an independent dataset to evaluate prediction performance. The Supplementary Table hows the species-specific prediction performance in terms of Pearson correlation for various ML models that were trained on the CV set and evaluated on the independent set. RF: random forest; GBDT: gradient boosting decision tree; ExtraTree: extra-tree regressor; ElasticNet: elastic net; LinearSVR: linear support vector regression.

**Strain**	**Ensemble APEX v2**	**Ensemble APEX v1**	**Single APEX**	**RF**	**GBDT**	**ExtraTree**	**ElasticNet**	**LinearSVR**
E. coli ATCC11775	0.847805	0.832404	0.824249	0.800211	0.808419	0.744944	0.722128	0.723382
P. aeruginosa PAO1	0.790273	0.769464	0.775476	0.771463	0.722799	0.771821	0.672709	0.58249
P. aeruginosa PA14	0.767182	0.746836	0.739504	0.725186	0.708727	0.736129	0.69213	0.593574
S. aureus ATCC12600	0.810676	0.81136	0.784346	0.70632	0.714723	0.551515	0.55703	0.598799
E. coli AIC221	0.688587	0.671029	0.693506	0.62584	0.654544	0.597245	0.580406	0.466094
E. coli AIC222	0.813293	0.803721	0.802091	0.736241	0.723161	0.676554	0.632948	0.607374
K. pneumoniae ATCC13883	0.633471	0.627873	0.67345	0.37494	0.451051	0.185642	0.325999	0.404414
A. baumannii ATCC19606	0.841729	0.842787	0.84471	0.818252	0.805014	0.796043	0.764364	0.674816
Methicillin-resistant *S. aureus* ATCC BAA-1556	0.859695	0.865534	0.786457	0.772415	0.78984	0.598027	0.578747	0.580319
Vancomycin-resistant *E. faecalis* ATCC700802	0.21569	0.101714	0.576916	0.119566	-0.51239	0	0	0.193324
Vancomycin-resistant *E. faecium* ATCC700221	0.736427	0.690436	0.741292	0.761104	0.642366	0.581336	0.684935	0.387822
Average	0.727712	0.705742	0.749272	0.655595	0.59166	0.567205	0.564672	0.528401

Supplementary Table 14 l Spearman correlation of various ML models on independent set. We divided our in-house peptide dataset into a CV set for hyperparameter tuning and ML model selection, and an independent dataset to evaluate prediction performance. The Supplementary Table hows the species-specific prediction performance in terms of Spearman correlation for various ML models that were trained on the CV set and evaluated on the independent set. RF: random forest; GBDT: gradient boosting decision tree; ExtraTree: extra-tree regressor; ElasticNet: elastic net; LinearSVR: linear support vector regression.

**Strain**	**Ensemble APEX v2**	**Ensemble APEX v1**	**Single APEX**	**RF**	**GBDT**	**ExtraTree**	**ElasticNet**	**LinearSVR**
E. coli ATCC11775	0.742143	0.738599	0.695429	0.70242	0.686571	0.675388	0.618169	0.623012
P. aeruginosa PAO1	0.701432	0.671522	0.620927	0.631221	0.673711	0.592132	0.545197	0.481929
P. aeruginosa PA14	0.725533	0.695124	0.619324	0.665001	0.613896	0.63482	0.604422	0.484608
S. aureus ATCC12600	0.578662	0.528803	0.52903	0.435649	0.497982	0.518397	0.419481	0.407387
E. coli AIC221	0.707444	0.707646	0.651341	0.670901	0.670596	0.676166	0.614667	0.583788
E. coli AIC222	0.721146	0.711075	0.666143	0.723491	0.626716	0.638027	0.609947	0.570258
K. pneumoniae ATCC13883	0.603606	0.581167	0.53605	0.28463	0.483702	0.190172	0.379504	0.31371
A. baumannii ATCC19606	0.683889	0.664927	0.661991	0.673022	0.625251	0.695621	0.624478	0.600003
Methicillin-resistant *S. aureus* ATCC BAA-1556	0.474364	0.456122	0.482894	0.44037	0.418149	0.570131	0.418258	0.447617
Vancomycin-resistant *E. faecalis* ATCC700802	0.238607	0.206435	0.361932	0.146958	-0.4407	0	0	0.184652
Vancomycin-resistant *E. faecium* ATCC700221	0.495449	0.436833	0.3834	0.427797	0.359027	0.333684	0.392771	0.397954
**Average**	**0.60657**	**0.581659**	**0.564406**	**0.527405**	**0.474082**	**0.502231**	**0.475172**	**0.463174**

Supplementary Table 15 l Pearson correlation of various ML models on modern human encrypted peptides. The human encrypted peptides from Torres et al.[1] constitute a subset of our in-house peptide dataset. We treated this subset as the test data and excluded it from ML model training. The Supplementary Table hows species-specific prediction performance in terms of Pearson correlation for various ML models.

Strain	Ensemble APEX v2	Ensemble APEX v1	Single APEX	RF	GBDT	ExtraTree	ElasticNet	LinearSVR	Torres *et al.*
E. coli ATCC11775	0.482757	0.496734	0.494723	0.435897	0.365328	0.407247	0.428736	0.320893	0.50776
P. aeruginosa PAO1	0.710869	0.686719	0.579761	0.731061	0.679557	0.699833	0.569554	0.143789	0.582418
P. aeruginosa PA14	0.734694	0.73291	0.671569	0.749065	0.68225	0.749057	0.725144	0.254412	0.712036
S. aureus ATCC12600	0.126253	0.186218	0.199264	0.076771	0.020007	0.07906	-0.01304	-0.26126	0.42482
E. coli AIC221	0.403527	0.404244	0.389027	0.521815	0.413622	0.483885	0.398567	0.200215	0.311953
E. coli AIC222	0.653576	0.655583	0.741654	0.680734	0.606786	0.762781	0.411687	0.316373	0.433937
K. pneumoniae ATCC13883	0.460277	0.466471	0.507818	0.456984	0.295749	-0.0989	0.22667	0.254919	0.282847
A. baumannii ATCC19606	0.773425	0.773293	0.73518	0.72433	0.760228	0.753661	0.510162	0.601455	0.430196
**Average**	**0.543172**	**0.550272**	**0.539874**	**0.547082**	**0.477941**	**0.479578**	**0.407185**	**0.228849**	**0.460746**

Supplementary Table 16 | Spearman correlation of various ML models on modern human encrypted peptides. The human encrypted peptides from Torres et al.[1] constitute a subset of our in-house peptide dataset. We treated this subset as the test data and excluded it from ML model training. The Supplementary Table hows species-specific prediction performance in terms of Spearman correlation for various ML models.

Strain	Ensemble APEX v2	Ensemble APEX v1	Single APEX	RF	GBDT	ExtraTree	ElasticNet	LinearSVR	Torres et al.
E. coli ATCC11775	0.523727	0.535223	0.506701	0.492004	0.370203	0.473523	0.440344	0.220609	0.497825
P. aeruginosa PAO1	0.719366	0.718463	0.622332	0.673903	0.575407	0.648483	0.626982	0.03871	0.615923
P. aeruginosa PA14	0.76658	0.760304	0.657554	0.657068	0.683707	0.662736	0.688632	0.225784	0.721803
S. aureus ATCC12600	0.254791	0.233065	0.259042	0.244744	0.081066	0.177344	0.025934	-0.20224	0.4654
E. coli AIC221	0.403575	0.397753	0.346453	0.491014	0.378764	0.376929	0.409659	0.137742	0.31409
E. coli AIC222	0.584407	0.582971	0.583851	0.589457	0.487067	0.636713	0.55179	0.17471	0.477801
K. pneumoniae ATCC13883	0.420755	0.417876	0.315581	0.313113	0.332973	-0.10474	0.440967	0.066277	0.293077
A. baumannii ATCC19606	0.634678	0.64788	0.602808	0.6092	0.62634	0.611794	0.513264	0.458603	0.455221
Average	0.538485	0.536692	0.48679	0.508813	0.441941	0.435348	0.462197	0.140025	0.480142

Supplementary Table 17 | Cytotoxicity assays performed using AEPs and MEPs. The cytotoxic activity was expressed in terms of CC_{50} values (μmol L^{-1}), *i.e.*, cytotoxic concentration values needed to damage 50% of the HEK293T cells present in each condition. The values were estimated by non-linear regressions based on the screen of all active AEPs and MEPs at concentrations from 8 to 128 μmol L^{-1}, to ensure coverage of all tested antimicrobial activity concentrations. The experiments were done in three independent biological replicates with two technical replicates within each biological replicate. The therapeutic index (TI) was calculated to show the margin of safety obtained by comparing the lowest MIC values (μmol L^{-1}) obtained in the antimicrobial activity assays to the CC_{50} values of each active AEP or MEP.

**Peptide**	**CC_{50} (μmol L^{-1})**	**MIC (μmol L^{-1})**	**TI**	**Peptide**	**CC_5 (μmol L^{-1})**	**MIC (μmol L^{-1})**	**TI**
Equusin-1	>128	1	>128	Megalocerin-1	>128	8	>16
Hesperelin-1	>128	2	>64	Pinguinusin-1	>128	4	>32
Elephasin-1	>128	4	>32	Ursusin-1	>128	64	>2
Arctodutin-1	>128	2	>64	Elephasin-2	>128	1	>128
Arctoterin-1	>128	64	>2	Mammuthusin-4	>128	2	>64
Lophiosin-1	68.02	16	>8	Psephotellin-1	>128	16	>8
Mammutin-1	>128	2	>64	Eudyptin-1	>128	64	>2
Ararin-1	>128	32	>4	Paleopropin-2	>128	16	>8
Mylodonin-1	>128	8	>16	Hydrodamin-2	>128	8	>16
Mammuthusin-1	>128	4	>32	Hydrodamin-3	>128	16	>8
Paleopropin-1	>128	32	>4	Hesperelin-4	>128	64	>2
Bisonin-1	>128	16	>8	Mylodonin-2	>128	32	>4
Hesperelin-2	>128	8	>16	Anomalopterin-2	>128	32	>4
Equusin-2	>128	8	>16	Equusin-3	>128	4	>32
Mammuthusin-2	>128	32	>4	Bisonin-2	>128	8	>16
Mammuthusin-3	>128	16	>8	Mammuthusin-5	>128	32	>4
Hydrodamin-1	>128	4	>32	Smilodin-1	>128	64	>2
Xenothrixin-1	70.77	8	8.84	Mylodonin-3	>128	16	>8
Hesperelin-3	>128	64	>2	Mylodonin-4	>128	32	>4
Ararin-2	>128	16	>8	Equusin-4	>128	16	>8
Anomalopterin-1	>128	64	>2				

Supplementary Table 18 l Method for the chromatography coupled to mass spectrometry experiments. The solvent gradient used is standard for small molecules and peptides and solvent used in the experiments are Fisher optima grades.

Time (min)	A (%)	B (%)	Flow rate (mL min^{-1})
0	95	5	0.5
0.5	95	5	0.5
2.5	5	95	0.5 (linear gradient)
3	5	95	0.5
3.25	5	95	0.5

References

1. Torres, M.D.T., Melo, M.C.R., Flowers, L., Crescenzi, O., Notomista, E., and de la Fuente-Nunez, C. (2022). Mining for encrypted peptide antibiotics in the human proteome. Nat Biomed Eng *6*, 67–75. 10.1038/s41551-021-00801-1.
2. Micsonai, A., Moussong, É., Wien, F., Boros, E., Vadászi, H., Murvai, N., Lee, Y.-H., Molnár, T., Réfrégiers, M., Goto, Y., et al. (2022). BeStSel: webserver for secondary structure and fold prediction for protein CD spectroscopy. Nucleic Acids Res *50*, W90–W98. 10.1093/nar/gkac345.

Description of Supplementary Datasets 1 to 3

Dataset 1 | List of EPs predicted by APEX to have a median MIC ≤ 80 μmol L^{-1}.

Dataset 2 | Information of EPs identified by APEX and validated experimentally. A total of 69 EPs predicted by APEX and selected based on our criteria were synthesized and experimentally validated. Their sequences, antimicrobial activities, and annotations are provided.

Dataset 3 | Information of EPs identified by a scoring function and validated experimentally. A total of 49 EPs predicted by a scoring function[1] were synthesized and experimentally validated. Their sequences and antimicrobial activities are provided.
